# Gracilosulfates A–G, Monosulfated Polyoxygenated Steroids from the Marine Sponge *Haliclona gracilis*

**DOI:** 10.3390/md18090454

**Published:** 2020-08-30

**Authors:** Larisa K. Shubina, Tatyana N. Makarieva, Vladimir A. Denisenko, Roman S. Popov, Sergey A. Dyshlovoy, Boris B. Grebnev, Pavel S. Dmitrenok, Gunhild von Amsberg, Valentin A. Stonik

**Affiliations:** 1 G.B. Elyakov Pacific Institute of Bioorganic Chemistry, Far Eastern Branch of the Russian Academy of Sciences, Pr. 100-let Vladivostoku 159, 690022 Vladivostok, Russia; shubina@piboc.dvo.ru (L.K.S.); vladenis@piboc.dvo.ru (V.A.D.); prs_90@mail.ru (R.S.P.); dyshlovoy@gmail.com (S.A.D.); grebnev_bor@mail.ru (B.B.G.); paveldmt@piboc.dvo.ru (P.S.D.); stonik@piboc.dvo.ru (V.A.S.); 2 Department of Oncology, Hematology and Bone Marrow Transplantation with Section Pneumology, Hubertus Wald-Tumorzentrum, University Medical Center Hamburg-Eppendorf, 20251 Hamburg, Germany; g.von-amsberg@uke.de; 3 Martini-Klinik, Prostate Cancer Center, University Hospital Hamburg-Eppendorf, 20251 Hamburg, Germany

**Keywords:** polyoxygenated steroids, sponge, *Haliclona gracilis*, anticancer activity

## Abstract

Seven new polyoxygenated steroids belonging to a new structural group of sponge steroids, gracilosulfates A–G (**1**–**7**), possessing 3*β*-*O*-sulfonato, 5*β,*6*β* epoxy (or 5(6)-dehydro), and 4*β*,23-dihydroxy substitution patterns as a common structural motif, were isolated from the marine sponge *Haliclona gracilis.* Their structures were determined by NMR and MS methods. The compounds **1, 2, 4, 6**, and **7** inhibited the expression of prostate-specific antigen (PSA) in 22Rv1 tumor cells.

## 1. Introduction

Marine organisms are known as a rich source of unique bioactive sulfate-containing metabolites [1]. Sulfated derivatives of different chemical classes (aliphatic compounds, steroids, terpenoids, carotenoids, aromatic compounds, alkaloids, carbohydrates, etc.) have been identified from them [1,2,3,4]. Marine invertebrates such as starfishes, ophiuroids, and ascidians contain mainly mono- and disulfated polyoxygenated steroids [1], which are almost exclusively marine secondary metabolites [1], while terrestrial sulfated polyoxygenated steroids are relatively rare. In fact, there is only one report concerning isolation of sulfated polyoxygenated steroid from plants [5]. 

Sulfated steroids represent one of the most numerous classes of sponge metabolites [1,2,3] Marine sponges provide a great structural diversity of bioactive sulfated polyoxygenated steroids, including nitrogen-containing [6], halogenated [7,8], monosulfated [9,10,11,12,13,14,15,16,17], disulfated [18], and trisulfated [7,8] steroids, as well as tetra- [19] or pentasulfated dimeric steroid derivatives [20]. The monosulfated polyoxygenated steroids account for only a part of these metabolites. Some of them show antimicrobial [13] and/or antifungal [10,13,15] and cytotoxic [10] activities or enhance glucose uptake via the AMPK signaling pathway [9].

During the search for bioactive compounds from the Northwestern Pacific deep-water marine invertebrates [21,22], we collected the pale orange sponge *Haliclona gracilis* near Shikotan Island, Russia, whose extract exhibited hemolytic and antifungal activities. 

The genus *Haliclona* (order Haplosclerida, family Halinidae) is represented by more than 600 species [23]. Marine sponges of *Haliclona* genus have been extensively examined, and more than 200 various bioactive metabolites including steroids, alkaloids, macrolides, polyketides, cyclic peptides, long-chain sphingoid bases, merohexaprenoids, and cyclic bis-1,3-dialkylpyridinium salts have been isolated, and different activities, including cytotoxic and antitumor effects, have been reported [1]. 

Sponges of *Haliclona* genus have provided very few sulfated steroids [1]. Thus, only two trisulfated steroids have been isolated in one Indo-Pacific *Haliclona* sponge [17], while monosulfated polyoxygenated steroids have never been isolated from this genus. Moreover, thus far, the sponge *H*. *gracilis* has not been chemically investigated. 

The ^1^H NMR analysis of the fractions obtained after diverse chromatographic separations suggested the presence of polar metabolites, inspiring our extensive investigation. Here, we report the details of the isolation and structure determination of compounds **1**–**7,** belonging to a new group of naturally occurring monosulfated polyoxygenated steroids with a 3*β*-*O*-sulfonato, 5*β*,6*β*-epoxy (or 5(6)-dehydro), or 4*β*,23-dihydroxy substitution pattern as a common structural motif. Additionally, anticancer activities of **1**, **2**, **4**, **6**, and **7** were evaluated.

## 2. Results and Discussion

**Figure 1:**
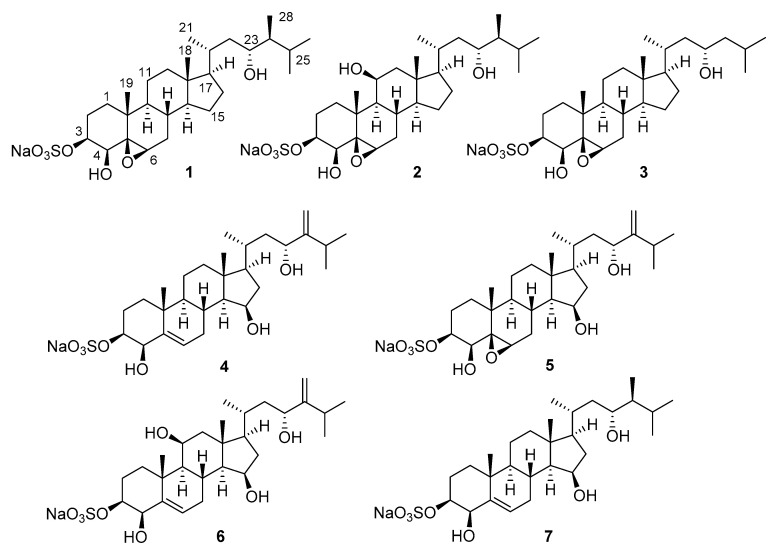
The structures of **1**–**7**.

The concentrated EtOH extract of the sponge was partitioned between *n*-BuOH and H_2_O. The organic extract was concentrated and the obtained residue was fractionated by flash chromatography on a YMC gel column. Further separation using reversed-phase HPLC resulted in the isolation of seven new steroids, gracilosulfates A–G (**1**–**7,** Figure 1).

Compound **1** was isolated as a white, amorphous solid. The molecular formula of **1** was determined to be C_28_H_47_NaO_7_S from the [M − Na]^−^ ion peak at *m/z* 527.3045 in the (−)HRESIMS spectrum. The fragment ion peak at *m/z* 97.9606 in the (−)HRESIMS/MS spectrum and absorption band at 1213 cm^−1^ in the IR spectrum revealed the presence of a sulfate group in **1**.

**Figure 2:**
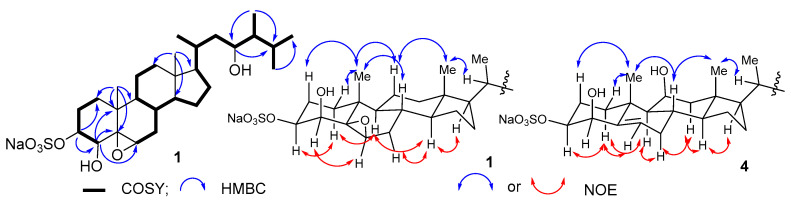
Key COSY and HMBC correlations for **1**, and NOESY correlations for **1** and **4**.

The ^1^H NMR spectrum of **1** (Table 1) showed signals attributable to six methyl groups at δ_H_ 1.16 (s), 0.94 (d), 0.91 (d), 0.82 (d), 0.74 (d), and 0.70 (s); four oxygen-bearing methine protons at δ_H_ 4.27 (d), 3.55 (br.d), 3.53 (br.d), and 3.17 (br.d); and a series of other methine and methylene multipletes. The ^13^C NMR (Table 2) and DEPT spectra of **1** revealed the presence of 28 signals, corresponding to 6 methyls, 8 methylenes, 11 methines, and 3 nonprotonated carbons (one bearing oxygen atom). These data evidenced a C-28 steroidal skeleton. Structure determination of **1** began with HMBC correlations from CH_3_-19 to C-1, C-5, C-9, and C-10. The COSY correlations (Figure 2) delineated the spin system H_2_-1 to H-4, which included protons of oxygenated methines at C-3 and C-4 based on their characteristic chemical shifts. The sequences of protons from H-6 to H-8, H-8 to H_2_-12, H-8 to H_3_-27, and H-24 to H_3_-28 were also established from COSY correlations and indicated the third oxymethine group at C-23. The cross peaks H-4/OH and H-23/OH in the COSY spectrum recorded in DMSO−*d*_6_ (Figure S11) and the ^13^C chemical shifts for C-4 (δ_C_ 77.7) and C-23 (δ_C_ 71.7) implied OH substitution, while the chemical shift for C-3 at δ_C_ 80.1 was more consistent with a sulfate half-ester O(SO_3_)Na [14]. The ^13^C NMR signals at δ_C_ 64.2 (CH) and 66.4 (C) and ^1^H NMR signal at δ_H_ 3.17 indicated the presence of trisubstituted epoxy ring [24]. The epoxy group was placed at C-5 and C-6 on the basis of HMBC correlations from H_2_-1, H-4, and H-19 to C-5 and from H-6 to C-4, C-8, and COSY correlations H-6/H_2_-7.

The large coupling constant of H-3 with H-2ax (*J* = 11.5 Hz) and small coupling constant of H-4 with H-3 (*J* = 3.0 Hz) pointed to *β* orientations for the 3-O(SO_3_)Na and 4-OH groups. The configuration of the 5*β*,6*β*-epoxy group was established by the NOESY correlations H-6/H-4. The same evidence was earlier used for β-orientation of epoxide group in a steroid from the soft coral *Dendronephthya gigantea* [25]. The key NOESY correlations H_3_-19/H-1*β,* H-2*β*, H-8, H-11*β*; H_3_-18/H-20, H-8, H-11*β*; H-9/H-1*α*, H-14; H-1*α* H-3*α;* H-4*α*/H-6; and H-17/H-14*α*, H_3_-21 confirmed the 3*β*,4*β*,5*β*,6*β* configurations of the oxygenated carbons and H-8*β*, H-9*α*, H-14*α*, and H-17*α* configurations of the ring portion in **1**. The 20*R* configuration was demonstrated by the NOESY cross-peak H_3_-18/H-20 and chemical shift value of CH_3_-21 at δ_H_ 0.94 [26].

**Figure 3:**
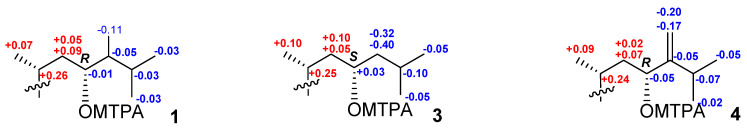
Δ*δ* values (*δ_S_* – *δ_R_*) for 23(*S*)- and 23(*R*)-MTPA esters of compounds **1**, **3,** and **4**.

**Figure 4:**
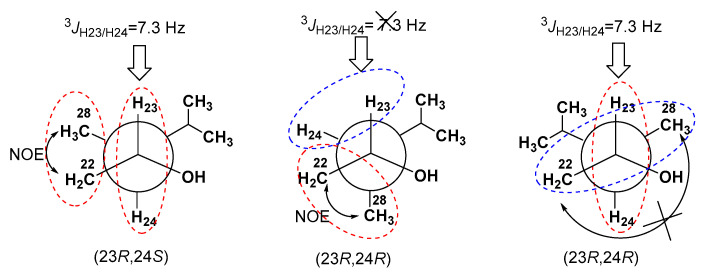
*J*-based configuration analysis and NOESY data of compound **1.**

The absolute configuration at C-23 was assigned by application of the Mosher’s method. Esterification of **1** with (*R*)- and (*S*)-α-methoxy-α-(trifluoromethyl)-phenylacetyl chlorides (MTPACl) yielded the 23-MTPA adducts **1S** and **1R**, respectively, while C-4 hydroxy group was not modified. Interpretation of ^1^H NMR chemical shift differences Δδ between **1S** and **1R** (Figure 3) revealed that the absolute configuration of C-23 is *R*. The *J*_H23/H24_ coupling constant was 7.3 Hz, which indicated *anti* relationship of the H-23 and H-24 protons [27] (Figure 4). The NOESY cross peak for H_2_-22/H_3_-28 suggested the *gauche* relationship between the C-22 methylene and C-28 methyl groups, as shown in Figure 3. These data allowed us to determine the 24*S* absolute configuration. 

Thus, the structure of **1** was defined as (20*R*,23*R*,24*S*)-4*β*,23-dihydroxy-5*β*,6*β*-epoxy-24- methylcholest-3*β* yl sulfate and was named gracilosulfate A.

The molecular formula of the second isolated compound, gracilosulfate B (**2**), was determined as C_28_H_47_NaO_8_S (*m/z* 543.3002 [M − Na]^−^) on the basis of the negative ion HRESIMS analysis. The ^1^H NMR data of **2** resembled those of **1**, except for the presence of an oxymethine group in **2** (δ_H_ 4.14) instead of a methylene group for **1**. Analysis of 1D and 2D NMR (COSY, HSQC, and HMBC) spectra allowed us to assign all the observed ^1^H and ^13^C signals for **2** (Table 1 and Table 2). The localization of the additional hydroxy group at C-11 followed from the HMBC correlations between H-11, C-8, and C-13 (Figure S16) and COSY data. The equatorial disposition of H-11 was evident from the small ^3^*J*_HH_ vicinal coupling to H-9 and H_2_-12 (Table 1) and confirmed by the relatively low field shift of H-8 (Table 1) caused by the 1,3-diaxial relationship of this proton to the hydroxy group at C-11. The configurations of other stereogenic centers of the ring portion were assigned using similar principles used for **1**. The similarity of the NMR data of the side chains of steroids **2** and **1** suggested the same (20*R*,23*R*,24*S*) configuration. Thus, gracilosulfate B was defined as (20*R*,23*R*,24*S*)-4*β*,11*β*,23-trihydroxy-5*β*,6*β*-epoxy-24-methylcholest-3*β* yl sulfate. 

The molecular formula of gracilosulfate C (**3**), determined as C_27_H_45_NaO_7_S from HRESIMS data (*m/z* 513.2891[M–Na]^−^), was one methylene unit less than that of **1**. The spectroscopic properties of **3** were similar to those of **1** and differed only by the signals of steroid side chain (Table 1 and Table 2). A combination of 2D NMR data showed the lack of a C-28 methyl group, while the remaining portion of the molecule was intact in **1**. The configuration of the ring moiety of **3** was assumed to be the same as that of **1** on the basis of the complete overlapping of proton and carbon resonances in NMR spectra. The configuration of the stereogenic center at C-23 was determined by the MTPA method as 23*S* (Figure 3). Thus, the gracilosulfate C was defined to be 24-demethyl derivative of gracilosulfate A (**1**), namely, (20*R*,23*S*)-4*β*,23-dihydroxy-5*β*,6*β*-epoxycholest-3*β* yl sulfate.

Gracilosulfate D (**4**) with a molecular formula C_28_H_45_NaO_7_S, confirmed by HRESIMS, was isolated as an optically active white amorphous solid. In addition to the signals relative to 3*β*-*O*-sulfonato-4*β*,23-dihydroxy structure, the ^1^H and ^13^C NMR spectra of **4** (Table 1 and Table 2) revealed signals of trisubstituted double bond (δ_H_ 5.70 and δ_C_ 144, 130.0), oxygenated methine group (δ_H_ 4.15 and δ_C_ 70.9), and terminal methylene group (δ_H_ 4.84, 5.03, and δ_C_ 106.8). The HMBC correlations between H_3_-19 and C-5 (δ_C_ 130.0), and between olefinic proton at δ_H_ 5.70 and C-4 (δ_C_ 77.6), were consistent with a double bond at C-5/C-6 position. The HMBC correlations from the oxymethine proton at δ_H_ 4.15 to C-13 and C-17 (Figure S32), in addition to COSY data (Figure S30), allowed placement of a hydroxy group at C-15 position, whereas the HMBC correlations between H_2_-28 and C-23, C-24, and C-25 confirmed the position of terminal methylene group at C-24. The coupling pattern associated with H-15 (ddd, *J* = 7.9, 5.8, 2.2 Hz) indicated that the hydroxy group at C-15 is *β*-positioned [28]. The configurations of other stereocenters of the steroid nucleus were assigned by NOESY (Figure 2) and coupling constants data (Table 1).

The absolute configuration at C-23 was deduced by theMTPA method. Treatment of **4** with (*R*)- and (*S*)- MTPACl yielded the corresponding 4,23-bis-MTPA adducts **4S** and **4R**, respectively. The Δ*δ* values around the C-23 stereocenter between the adducts **4S** and **4R** (Figure 3) indicated the 23*S* configuration and, therefore, the structure of **4** was assigned as (20*R*, 23*S*)-4*β*,15*β*,23-trihydroxy-24-methylenecholest-5(6)-en-3*β*yl sulfate. 

The molecular formula of C_28_H_45_NaO_8_S was assigned by HRESIMS to gracilosulfate E (**5**). The 1D (Table 1 and Table 2) and 2D NMR analysis showed that gracilosulfate E (**5**) differs from **4** in the 5,6-epoxy group, replacing trisubstituted double bond. The configurations of the ring moiety were assigned on the basis of the analyses of proton–proton coupling constants (Table 1) and NOESY data. The absolute configuration of the side chain of **5** was determined to be the same as in **4** by comparison of ^1^H and ^13^C chemical shifts. Thus, gracilosulfate E (**5)** was determined to be (20*R*,23*R*)-4*β*,15*β*,23-trihydroxy-5*β*,6*β*-epoxy-24-methylenecholest-3*β* yl sulfate. 

Gracilosulfate F (**6**) of molecular formula C_28_H_45_NaO_8_S was a close analogue of gracilosulfate D (**4**) showing only an additional oxygen atom. Inspection of 1D (Table 1 and Table 2) and 2D NMR data allowed placement of an additional hydroxy group at C-11. The configuration at C-11 was deduced from NOESY correlation of H-11 to axial proton H-1 and small vicinal coupling constant of H-11 (Table 1), which is consistent with an equatorial disposition for this proton, thereby placing the hydroxy group in an axial position. The configurations of remaining stereogenic centers of the ring portion were the same as those of **4**, as established on the basis of analyses of proton–proton coupling constants (Table 1 and Table 2) and NOESY data. The absolute configuration of the side chain was determined to be the same as that of 4 by comparison of ^1^H and ^13^C chemical shifts, and finally the structure of **6** was established as (20*R*, 23*R*)-4*β*,11*β*,15*β*,23-tetrahydroxy-24-methylenecholest-5(6)-en-3*β*yl sulfate.

Gracilosulfate G (**7**) showed the molecular formula C_28_H_47_NaO_7_S as determined by HRESIMS. On the basis of the results of the 1D NMR spectra, we were able to assign a trisubstituted double bond and four oxygen-bearing methine groups. The same steroid core constitution and configurations as in gracilosulfate D (**4**) were inferred from 1D (Table 1 and Table 2) and 2D NMR analysis. The proton and carbon resonances attributable to the side chain of **7** were coincident with those of **1** and **2** (Table 1 and Table 2). Thus, gracilosulfate G was defined as (20*R*, 23*R*, 24*S*)-4*β*,15*β*,23-trihydroxy-24-methylcholest-5(6)-en-3*β*yl sulfate. 

**Figure 5:**
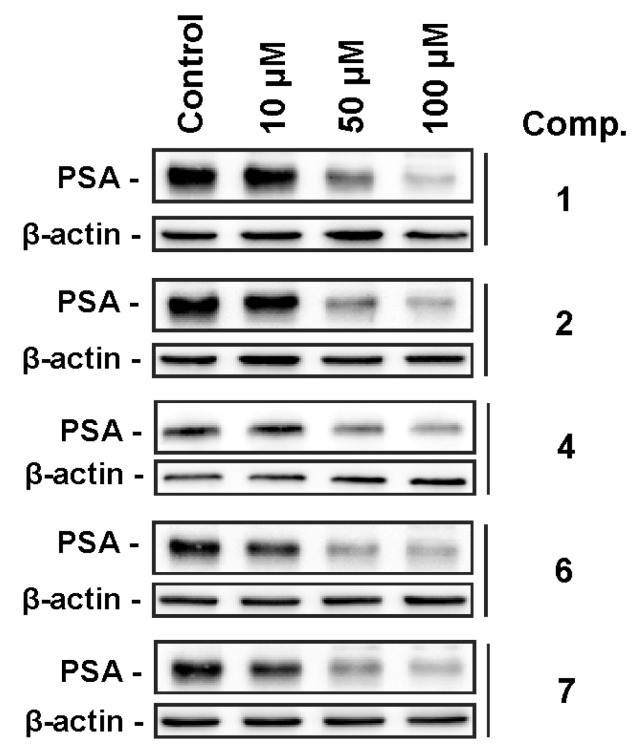
Effects of the compounds on PSA expression in 22Rv1 cells. The cells were treated with the compounds for 24 h, then the proteins were extracted and examined using Western blotting. β-actin was used as a loading control.

Next, antitumor activity of compounds **1**, **2**, **4**, **6**, and ***7*** were determined in human prostate cancer cells 22Rv1. Of note, this cell line reveals resistance to androgen receptor (AR)-targeted therapy due to the expression of AR-V7 (AR transcript variant V7), which lacks the androgen-binding site [29,30]. The compounds exhibited moderate cytotoxic activity in the cancer cells after 48 h of treatment. Thus, compound **7** exhibited IC_50_ = 64.4 ± 14.9 µM, while the other tested compounds had IC_50_ > 100 µM (docetaxel was used as a positive control and exhibited IC_50_ = 17.3 ± 6.3 nM). However, all compounds were able to effectively inhibit the expression of PSA (prostate-specific antigen) in 22Rv1 cells (Figure 5). Earlier, only two monosulfated polyoxygenated steroids have been shown to exert cytotoxic activity on human cancer cell lines [10]. On the other hand, non-sulfated polyoxygenated steroid aragusterol with potent antitumor activities was isolated from a sponge of the genus *Xestospongia* [31]. Interestingly, for compounds **6** and **7**, this effect was already detected at a concentration of 10 µM. PSA is a well-known downstream target of AR signaling. Thus, suppression of PSA expression may indicate an inhibition of this pathway. AR signaling is essential for the growth and survival of prostate cancer cells, with its targeting playing a central role in the modern therapy of advanced prostate cancer. The ability of the isolated compounds to suppress AR signaling can be explained by the similarity of their structures to androgen ligands, which may result in a binding to androgen receptors and therefore blocking of AR-mediated signaling in prostate cancer cells. 

## 3. Materials and Methods

### 3.1. General Procedures

Optical rotations were measured using a PerkinElmer 343 polarimeter (Waltham, MA, USA). IR spectra were recorded using spectrophotometer Equinox 55 (Bruker, Ettlingen, Germany). The ^1^H and ^13^C NMR spectra were obtained using Bruker Avance III-700 and Bruker Avance III HD-500 spectrometers (Bruker, Ettlingen, Germany). Chemical shifts were referenced with Me_4_Si as an internal standard. ESI mass spectra (including HRESIMS) were measured using Bruker maXis Impact II mass spectrometer (Bruker Daltonics, Bremen, Germany). Low-pressure column liquid chromatography was performed using YMC Gel ODS-A (YMC Co., Ltd., Kyoto, Japan). HPLC was performed using Shimadzu Instrument equipped with RID-10A refractive index detector (Shimadzu Corporation, Kyoto, Japan) and YMC-Pack ODS-A (250 × 10 mm) column (YMC Co., Ltd., Kyoto, Japan). 

### 3.2. Animal Material

Specimens of *Haliclona gracilis* were collected off the coast of Shikotan Island (43°28′0 N; 146°48′9 E) by dredging at 145 m depth on June 2017, and identified by Grebnev B. B. using the morphology of skeleton and spicules. Comparison the data of #050-078 with the corresponding characteristics of *Haliclona gracilis* and their complete coincidence supported the sponge identification as *Haliclona gracilis* [32]. A voucher specimen is deposited under registration number 050-078 in the collection of marine invertebrates of the Pacific Institute of Bioorganic Chemistry (Vladivostok, Russia).

### 3.3. Extraction and Isolation

The freshly collected specimens were immediately frozen and stored at −18 °C until use. Animal material (dry weight 20 g) were crushed and extracted with EtOH (2 × 1 L). The EtOH extract after evaporation in vacuo was partitioned between H_2_O and *n*-BuOH. The *n*-BuOH-soluble materials were partitioned with aqueous EtOH and *n*-hexane. The EtOH-soluble layer was fractioned by flash column chromatography on YMC gel ODS-A (75 μm), eluting with a step gradient of H_2_O – EtOH (100:0 − 20:80) with monitoring by HPLC. The fractions that eluted with 40% EtOH were further purified by repeated reversed-phase HPLC (YMC ODS-A column (250 × 10 mm), 1.5 mL/min, H_2_O-EtOH, 40:60 +1% AcONH_4_) to afford, in order of elution, compounds **6** (2 mg), **2** (3 mg), **4** (6 mg), **5** (1 mg), **3 (**1 mg), **7** (4 mg), and **1** (8 mg) with retention times (*t*_R_) of 14.0, 17.5, 18.4, 22.5, 26.2, 32.5, and 36.1 min, respectively.

### 3.4. Compound Characterization Data

*Gracilosulfate A (**1**)*: white, amorphous solid; [α]${}_{D}^{20}$ +6 (*c* 0.2, EtOH); IR (KBr) ν_max_ 3467, 2957, 1457, 1242, 1002, 939 cm^−1^; ^1^H, ^13^C NMR, Table 1 and Table 2; HRESIMS *m/z* 527.3045 [M−Na]^−^ (calcd for C_28_H_47_O_7_S, 527.3048). 

*Gracilosulfate B (**2**)*: white, amorphous solid; [α]${}_{D}^{20}$ +22 (*c* 0.2, EtOH); IR (KBr) ν_max_ 3446, 2947, 1457, 1242, 937 cm^−1^; ^1^H, ^13^C NMR, Table 1 and Table 2; HRESIMS *m/z* 543.3002 [M−Na]^−^ (calcd for C_28_H_47_O_8_S, 543.2997). 

*Gracilosulfate C (**3**)*: white, amorphous solid; [α]${}_{D}^{20}$ ≈0 (*c* 0.1, EtOH); IR (KBr) ν_max_ 3465, 2960, 1450, 1240 cm^−1^; ^1^H, ^13^C NMR, Table 1 and Table 2; HRESIMS *m/z* 513.2891 [M−Na]^−^ (calcd for C_27_H_45_O_7_S, 513.2891).

*Gracilosulfate D (**4**)*: white, amorphous solid; [α]${}_{D}^{20}$ −40 (*c* 0.2, EtOH); IR (KBr) ν_max_ 3436, 2956, 1457, 1242, 1065, 998 cm^−1^; ^1^H, ^13^C NMR, Table 1 and Table 2; HRESIMS *m/z* 525.2890 [M−Na]^−^ (calcd for C_28_H_45_O_7_S, 525.2891).

*Gracilosulfate E (**5**)*: white, amorphous solid; [α]${}_{D}^{20}$ ~0 (*c* 0.1, EtOH); IR (KBr) ν_max_ 3440, 2938, 1457, 1241, 936 cm^−1^; ^1^H, ^13^C NMR, Table 1 and Table 2; HRESIMS *m/z* 541.2845 [M−Na]^−^ (calcd for C_28_H_45_O_8_S, 541.2841).

*Gracilosulfate F (**6**)*: white, amorphous solid; [α]${}_{D}^{20}$ −17 (*c* 0.2, EtOH); IR (KBr) ν_max_ 3456, 2942, 1457, 1242, 998 cm^−1^; ^1^H, ^13^C NMR, Table 1 and Table 2; HRESIMS *m/z* 541.2840 [M−Na]^−^ (calcd for C_28_H_45_O_8_S, 541.2841).

*Gracilosulfate G (**7**)*: white, amorphous solid; [α]${}_{D}^{20}$ −32 (*c* 0.1, EtOH); IR (KBr) ν_max_ 3440, 2932, 1653, 1457, 1240 cm^−1^; ^1^H, ^13^C NMR, Table 1 and Table 2; HRESIMS *m/z* 527.3057 [M − Na]^−^ (calcd for C_28_H_47_O_7_S, 527.3048).

#### Preparation of MTPA esters of compounds **1**, **3**, and **4**

To duplicate solutions of compound **1** (2 mg each) in 100 µL of anhydrous pyridine, we added (*R*)- or (*S*)-MTPACl (10 μL). After stirring for 30 min at rt, the reaction mixtures were concentrated under reduced pressure and separated by HPLC (YMC ODS-A column (250 × 10 mm), H_2_O-EtOH, 24:76 + 1% AcONH_4_) to afford the (*S*)- or (*R*)-MTPA esters of **1**. The (*S*)- or (*R*)-MTPA derivatives of **3** and **4** were also prepared in a similar manner.

*(S)-MTPA ester of **1** (**1S**):* white, amorphous solid; ^1^H NMR (CD_3_OD, 500 MHz) δ_H_ 5.35 (1H, dd, *J* = 11.2, 4.7 Hz, H-23), 1.76 (1H, m, H-22), 1.52 (1H, m, H-24), 1.47 (1H, m, H-25), 1.39 (1H, m, H-20), 1.13 (1H, m, H-22), 0.99 (3H, d, *J* = 6.7 Hz, H-21), 0.94 (3H, d, *J* = 6.6 Hz, H-27), 0.86 (3H, d, *J* = 6.6 Hz, H-26), 0.76 (3H, d, *J* = 6.7 Hz, H-28), 0.62 (3H, s, H-18). HRESIMS *m/z* 779.3210 [M + Cl]^−^ (calcd for C_38_H_55_ClF_3_O_9_S, 779.3213).

*(R)-MTPA ester of **1*** (***1R***)*:* white, amorphous solid; ^1^H NMR (CD_3_OD, 500 MHz) δ_H_ 5.36 (1H, dd, *J* = 11.2, 4.7 Hz, H-23), 1.69 (1H, m, H-22), 1.57 (1H, m, H-24), 1.50 (1H, m, H-25), 1.13 (1H, m, H-20), 1.04 (1H, m, H-22), 0.97 (3H, d, *J* = 6.6 Hz, H-27), 0.92 (3H, d, *J* = 6.7 Hz, H-21), 0.89 (3H, d, *J* = 6.6 Hz, H-26), 0.87 (3H, d, *J* = 6.7 Hz, H-28), 0.45 (3H, s, H-18). HRESIMS *m/z* 779.3210 [M + Cl]^−^ (calcd for C_38_H_55_ClF_3_O_9_S, 779.3213).

*(S)-MTPA ester of***3** (**3*S***)*:* white, amorphous solid; ^1^H NMR (CD_3_OD, 500 MHz) δ_H_ 5.33 (1H, m, H-23), 1.78 (1H, m, H-22), 1.49 (1H, septet, *J* = 6.6 Hz,, H-25), 1.41 (1H, m, H-20), 1.30 (1H, m, H-24), 1.19 (1H, m, H-22), 0.99 (3H, d, *J* = 6.5 Hz, H-21), 0.98 (1H, m, H-24), 0.90 (3H, d, *J* = 6.6 Hz, H-27), 0.87 (3H, d, *J* = 6.6 Hz, H-26), 0.63 (3H, s, H-18). HRESIMS *m/z* 765.3060 [M + Cl]^−^ (calcd for C_37_H_53_ClF_3_O_9_S, 765.3056).

*(R)-MTPA ester of **3** (**3R**):* white, amorphous solid; ^1^H NMR (CD_3_OD, 500 MHz) δ_H_ 5.30 (1H, m, H-23), 1.62 (1H, m, H-24), 1.68 (1H, m, H-22), 1.38 (1H, m, H-24), 1.59 (1H, septet, *J* = 6.6 Hz,, H-25), 1.16 (1H, m, H-20), 1.14 (1H, m, H-22), 0.89 (3H, d, *J* = 6.5 Hz, H-21), 0.95 (3H, d, *J* = 6.6 Hz, H-27), 0.92 (3H, d, *J* = 6.6 Hz, H-26), 0.42 (3H, s, H-18). HRESIMS *m/z* 765.3060 [M + Cl]^−^ (calcd for C_37_H_53_ClF_3_O_9_S, 765.3056).

*Bis(S)-MTPA ester of **4** (**4S**):* white, amorphous solid; ^1^H NMR (CD_3_OD, 500 MHz) δ_H_ 6.02 (1H, dd, *J* = 3.3, 1.1 Hz, H-4), 5.47 (1H, brd, *J* = 11.1 Hz, H-23), 4.87 (1H, t, *J* = 1.2 Hz, H-28), 4.84 (1H, brs, H-28), 1.91 (1H, m, H-22), 2.25 (1H, septet, *J* = 6.6 Hz, H-25), 1.67 (1H, m, H-20), 1.28 (1H, m, H-22), 1.10 (3H, d, *J* = 6.6 Hz, H-27), 1.03 (6H, d, *J* = 6.6 Hz, H-21, 26), 0.93 (3H, s, H-18). HRESIMS *m/z* 957.3675 [M − Na]^−^ (calcd for C_48_H_59_F_6_O_11_S, 957.3688).

*Bis(R)-MTPA ester of **4** (**4R**):* white, amorphous solid; ^1^H NMR (CD_3_OD, 500 MHz) δ_H_ 5.91 (1H, dd, *J* = 3.3, 1.1 Hz, H-4), 5.52 (1H, brd, *J* = 11.1 Hz, H-23), 5.07 (1H, t, *J* = 1.2 Hz, H-28), 5.01 (1H, brs, H-28), 2.32 (1H, septet, *J* = 6.6 Hz, H-25), 1.89 (1H, m, H-22), 1.43 (1H, m, H-20), 1.21 (1H, m, H-22), 0.94 (3H, d, *J* = 6.5 Hz, H-21), 1.12 (3H, d, *J* = 6.6 Hz, H-27), 1.08 (3H, d, *J* = 6.6 Hz, H-26), 0.55 (3H, s, H-18). HRESIMS *m/z* 957.3675 [M − Na]^−^ (calcd for C_48_H_59_F_6_O_11_S, 957.3688).

### 3.5. Bioactivity Assay

#### 3.5.1. Reagents

The MTT reagent (thiazolyl blue tetrazolium bromide) was purchased from Sigma (Taufkirchen, Germany).

#### 3.5.2. Cell Lines and Culture Conditions

The human prostate cancer cell line 22Rv1 was purchased from ATCC (Manassas, VA, USA). Cells were cultured according to the manufacturer’s instructions in RPMI media containing 10% FBS (Invitrogen, Carlsbad, USA). Cells were continuously kept in culture for a maximum of 3 months, and were routinely examined for stable phenotype and mycoplasma contamination.

#### 3.5.3. In Vitro MTT-Based Drug Sensitivity Assay

The in vitro cytotoxicity of individual substances was evaluated using a MTT-based assay, which was performed as previously described [33]. Treatment time was 48 h.

#### 3.5.4. Western Blotting

Preparation of protein extracts and Western blotting were performed as described previously [34]. For the detection of PSA, expression the anti-PSA/KLK3 antibodies was used (Cell Signaling, #5365, 1:1000). Treatment time was of 24 h.

## 4. Conclusions

In summary, we isolated gracilosulfates A-G, new steroids from the marine sponge *H. gracilis*, possessing a rare 3β-O-sulfonato, 4β-hydroxy moiety [1]. To date, only one pregnane steroid [35] and two polyhydroxy steroids [36] with such a fragment have been isolated from the sponge *Stylopus australis* and the starfish *Coscinasterias tenuispina,* respectively. In addition, the 5*β*,6*β* epoxy fragment is unprecedented in sulfated steroids [1]. Finally, the combination of 3*β*-*O*-sulfonato, 5*β*,6*β*-epoxy (or 5(6)-dehydro), and 4*β*,23-dihydroxy moieties is unprecedented, taking into account structures of all previously known natural sulfated steroids. Interestingly, these compounds are able to inhibit PSA expression in human hormone-independent prostate cancer cells, suggesting inhibition of AR signaling, a central target for the treatment of advanced prostate cancer.

## Figures and Tables

**Table 1:** ^1^H NMR data for compounds **1**–**7** in CD_3_OD.

Position	1	2	3	4	5	6	7
	δ_H_ (*J* in Hz)	δ_H_ (*J* in Hz)	δ_H_ (*J* in Hz)	δ_H_ (*J* in Hz)	δ_H_ (*J* in Hz)	δ_H_ (*J* in Hz)	δ_H_ (*J* in Hz)
1α	1.39, m	1.37, m	1.37, m	1.15, m	1.37, m	1.31, m	1.15, m
1β	1.05, m	2.10, m	2.06, m	1.90, m	2.07, m	2.12, dt (13.3, 3.3)	1.89, dt (13.3, 3.3)
2α	1.82, m	1.84, m	1.82, m	1.81, m	1.83, m	1.84, m	1.82, m
2β	2.10, m	2.12, m	2.10, m	2.11, m	2.11, m	2.21, m	2.12, m
3	4.27, ddd (11.5, 4.1, 3.0)	4.29, ddd (11.5, 4.1, 3.0)	4.27, ddd (11.5, 4.1, 3.0)	4.18, ddd (12.2, 4.3, 3.3)	4.28, ddd (11.5, 4.1, 3.0)	4.19, ddd (12.0, 3.9, 3.1)	4.18, ddd (11.7, 4.0, 3.1)
4	3.55, br d (3.0)	3.58, br d (3.0)	3.56, br d (3.0)	4.42, dd (3.3, 1.3)	3.57, br d (3.0)	4.40, dd (3.3, 1.3)	4.42, dd (3.3, 1.3)
5							
6	3.17, br d (2.7)	3.12, br d (2.7)	3.17, br d (2.5)	5.70, dd (5.0, 2.4)	3.19, br d (2.5)	5.59, dd (4.2, 3.0)	5.70, dd (5.0, 2.4)
7α 7β	1.29, m 2.08, m	1.35, m 2.19, m	1.27, m 2.08, m	1.68, ddd (18.2, 10.3, 2.3) 2.40, m	1.32, m 2.39, m	1.80, ddd (18.0, 9.8, 2.6) 2.53, ddd (18.8, 6.6, 4.3)	1.68, ddd (18.0, 10.3, 2.3) 2.39, m
8	1.43, m	1.79, m	1.42, m	1.99, dd (10.8, 5.7)	1.85, m	2.42, m	1.99, m
9	0.68, dd (12.0, 4.5)	0.78 dd, (11.5, 3.0)	0.68, dd, (11.6, 4.7)	0.98, m	0.74, m	1.01, m	0.98, m
11α 11 β	1.40, m 1.43, m	4.14, br q, (3.0)	1.40, m 1.44, m	1.50, m 1.52, m	1.38, m 1.43, m	4.29, br q, (3.4)	1.49, m 1.51, m
12α	1.14, m	1.30, m	1.12, m	1.18, m	1.10, m	1.36, m	1.18, m
12 β	2.02, m	2.23, dd, (13.3, 3.0)	2.02, m	2.03, dt (12.6, 3.6)	1.97, dt (12.3, 3.7)	2.22, m	2.03, dt (12.5, 3.3)
13							
14	0.95, m	0.94, m	0.94, m	0.90, m	0.78, dd (11.3, 5.7)	0.89, m	0.90, m
15	1.64, m 1.05, m	1.65, m 1.15, m	1.64, m 1.06, m	4.15, ddd (7.9, 5.8, 2.2)	4.15, ddd (8.1, 5.7, 2.3)	4.18, ddd (7.8, 5.7, 2.2)	4.15, ddd (7.7, 5.6, 2.0)
16	1.86, m 1.36, m	1.83, m 1.39, m	1.86, m 1.35, m	2.40, m 1.41, ddd (14.3, 10.4, 2.3)	2.36, m 1.37, m	2.37, m 1.41, m	2.40, m 1.39, m
17	1.08, m	1.03, m	1.08, m	1.05, m	1.02, m	1.00, m	1.07, m
18	0.70, s	0.93, s	0.69, s	1.00, s	0.94, s	1.20, s	0.99, s
19	1.16, s	1.43, s	1.16, s	1.24, s	1.19, s	1.49, s	1.24, s
20	1.73, m	1.72, m	1.72, m	1.88, m	1.86, m	1.88, m	1.87, m
21	0.94, d (6.7)	0.96, d (6.7)	0.95, d (6.7)	1.02, d (6.7)	0.99, d (6.7)	1.04, d (6.7)	0.97, d (6.7)
22	1.41, m 1.04, m	1.39, m 1.03, m	1.48, m 0.98, m	1.59, ddd (13.7, 10.3, 2.3) 1.11, m	1.57, ddd (14.1, 10.5, 2.7) 1.10, m	1.58, ddd (14.1, 10.5, 2.5) 1.11, m	1.43, 1.07, m
23	3.53, ddd (9.1, 7.3, 2.0)	3.53, ddd (9.4, 7.3, 2.0)	3.70, m	4.13, br d (10.5)	4.11, br d (10.5)	4.13, br d (10.5)	3.55, ddd (9.3, 7.1, 2.0)
24	1.29, m	1.28, m	1.38, m 1.14, m				1.31, m
25	1.91, m	1.91, m	1.75, m	2.26, septet (6,7)	2.24, septet (6,7)	2.25, septet (6,7)	1.93, m
26	0.82, d (6.6)	0.82, d (6.6)	0.90, d (6.8)	1.06, d (6.9)	1.05, d (6.8)	1.06, d (6.8)	0.83, d (6.9)
27	0.91, d (6.6)	0.91, d (6.6)	0.91, d (6.8)	1.08, d (6.9)	1.07, d (6.8)	1.08, d (6.8)	0.92, d (6.9)
28	0.74, d (6.8)	0.74, d (6.8)		5.03, t (1.2) 4.84, br s	5.03, t (1.2) 4.84, br s	5.03, t (1.2) 4.84, br s	0.75, d

**Table 2:** ^13^C NMR data^a^ for compounds **1–7** in CD_3_OD.

Position	1	2	3	4	5	6	7
	δ_C_, Type	δ_C_, Type	δ_C_, Type	δ_C_, Type	δ_C_, Type	δ_C_, Type	δ_C_, Type
1	39.2, CH_2_	40.2, CH_2_	39.2, CH_2_	39.2, CH_2_	39.2, CH_2_	38.2, CH_2_	39.4, CH_2_
2	23.7, CH_2_	24.1, CH_2_	23.7, CH_2_	24.6, CH_2_	23.7, CH_2_	24.0, CH_2_	24.4, CH_2_
3	80.1, CH	79.9, CH	80.1, CH	82.5, CH	80.1, CH	82.0, CH	82.1, CH
4	77.7, CH	78.0, CH	77.7, CH	77.6, CH	77.7, CH	76.9, CH	77.6, CH
5	66.4, C	66.2, C	66.4, C	144.1, C	66.3, C	145.4, C	144.1, C
6	64.2, CH	63.3, CH	64.2, CH	130.0, CH	64.2, CH	129.5, CH	130.0, CH
7	34.2, CH_2_	33.6, CH_2_	34.2, CH_2_	33.0, CH_2_	33.6, CH_2_	33.2, CH_2_	33.0, CH_2_
8	31.7, CH	28.8, CH	31.7, CH	29.4, CH	27.4, CH	26.4, CH	29.5, CH
9	53.9, CH	57.8, CH	53.9, CH	52.9, CH	54.3, CH	56.2, CH	59.2, CH
10	36.9, C	37.9, C	36.9, C	38.0, C	36.9, C	38.6, C	38.1, C
11	23.1, CH_2_	69.5, CH	23.1, CH_2_	22.1, CH	23.1, CH_2_	69.5, CH	22.2, CH
12	41.8, CH_2_	51.1, CH_2_	41.8, CH_2_	43.0, CH_2_	43.1, CH_2_	52.3, CH_2_	43.0, CH_2_
13	44.1, C	43.7, C	44.1, C	44.0, C	43.9, C	43.4, C	44.0, C
14	58.1, CH	61.2, CH	58.1, CH	63.5, CH	62.8, CH	65.6, CH	63.6, CH
15	25.9, CH_2_	25.7, CH_2_	25.9, CH_2_	71.3, CH	70.9, CH	71.3, CH	71.2, CH
16	29.8, CH_2_	29.7, CH_2_	29.8, CH_2_	42.7, CH_2_	42.6, CH_2_	42.3, CH_2_	42.6, CH_2_
17	59.0, CH	59.8, CH	58.9, CH	59.1, CH	59.0, CH	59.9, CH	59.4, CH
18	12.8, CH_3_	16.1, CH_3_	12.8, CH_3_	15.7, CH_3_	15.4, CH_3_	18.1, CH_3_	15.6, CH_3_
19	19.1, CH_3_	21.8, CH_3_	19.1, CH_3_	22.0, CH_3_	19.0, CH_3_	25.3, CH_3_	21.9, CH_3_
20	34.1, CH	34.2, CH	34.1, CH	34.2, CH	34.2, CH	34.3, CH	33.9, CH
21	19.5, CH_3_	19.5, CH_3_	19.7, CH_3_	19.6, CH_3_	19.5, CH_3_	19.5, CH_3_	19.7, CH_3_
22	41.7, CH_2_	41.8, CH_2_	46.2, CH_2_	45.0, CH_2_	45.1, CH_2_	45.1, CH_2_	41.9, CH_2_
23	71.7, CH	71.8, CH	68.1, CH	72.5, CH	72.5, CH	72.6, CH	71.8, CH
24	47.4, CH	47.4, CH	49.5, CH	162.4, C	162.3, C	162.4, C	47.4, CH
25	29.6, CH	29.6, CH	26.4, CH	32.2, CH	32.1, CH	32.2, CH	29.6, CH
26	22.5, CH_3_	22.5, CH_3_	24.4, CH_3_	24.4, CH_3_	24.4, CH_3_	24.4, CH_3_	22.5, CH_3_
27	18.3, CH_3_	18.4, CH_3_	23.3, CH_3_	23.6, CH_3_	23.7, CH_3_	23.7, CH_3_	18.4, CH_3_
28	11.3, CH_3_	11.3, CH_3_		106.8, CH_2_	106.8, CH_2_	106.8, CH_2_	11.4, CH_3_

^a^ Assignments were confirmed by HSQC and HMBC (8Hz) data.

## Supplementary Materials

The following are available online at https://www.mdpi.com/1660-3397/18/9/454/s1, Copies of HRESIMS, and 1D- and 2D-NMR spectra of **1**–**7**, and photo of the marine sponge *Haliclona gracilis* (#050-078).
