# Supplementary material for: Gracilosulfates A–G, Monosulfated Polyoxygenated Steroids from the Marine Sponge Haliclona gracilis
Source: Mar Drugs. 2020 Aug 30;18(9):454. doi: 10.3390/md18090454 (PMC7551063; doi:10.3390/md18090454)

Supporting Information

Gracilosulfates A-G, Monosulfated Polyoxygenated Steroids from the Marine Sponge *Haliclona gracilis*

Larisa K. Shubina[1], Tatyana N. Makarieva[1,*], Vladimir A. Denisenko[1], Roman S. Popov[1], Sergey A. Dyshlovoy[1,2,3], Boris B. Grebnev[1], Pavel S. Dmitrenok[1], Gunhild von Amsberg[2,3], and Valentin A. Stonik[1]

[1] G. B. Elyakov Pacific Institute of Bioorganic Chemistry, Far-Eastern Branch of the Russian Academy of Sciences, Prospect 100-let Vladivostoku 159, Vladivostok 690022, Russian Federation

[2] Laboratory of Experimental Oncology, Department of Oncology, Hematology and Bone Marrow Transplantation with Section Pneumology, Hubertus Wald-Tumorzentrum, University Medical Center Hamburg-Eppendorf, Hamburg, Germany

[3] Martini-Klinik, Prostate Cancer Center, University Hospital Hamburg-Eppendorf, Hamburg, Germany

[*] To whom correspondence should be addressed. Tel: 7 (4232) 31-11`-68. Fax: 7 (4232) 31-40-50. E-mail: makarieva@piboc.dvo.ru

Contents

Figure S1. The ^{1}H NMR spectrum of compound **1** in CD_3OD S4

Figure S2. The ^{13}C NMR spectrum of compound **1** in CD_3OD S5

Figure S3. The COSY spectrum of compound **1** in CD_3OD S6

Figure S4. The HSQC spectrum of compound **1** in CD_3OD S7

Figure S5. The HMBC spectrum of compound **1** in DMSO-d_6 S8

Figure S6. The NOESY spectrum of compound **1** in CD_3OD S9

Figure S7. The HRESIMS spectrum of compound **1** S10

Figure S8. The ^{1}H NMR chemical shift differences between **1** (*S*)- and **1** (*R*)-MTPA esters S11

Figure S9. The HRESIMS spectrum of compound **1S** (**1R**) S12

Figure S10. The ^{1}H NMR spectrum of compound **1** in DMSO-d_6 S13
Figure S11. The COSY spectrum of compound **1** in DMSO-d_6 S14
Figure S12. The ^{1}H NMR spectrum of compound **2** in CD_3OD S15
Figure S13. The ^{13}C NMR spectrum of compound **2** in CD_3OD S16
Figure S14. The COSY spectrum of compound **2** in CD_3OD S17
Figure S15. The HSQC spectrum of compound **2** in CD_3OD S18
Figure S16. The HMBC spectrum of compound **2** in CD_3OD S19
Figure S17. The NOESY spectrum of compound **2** in CD_3OD S20
Figure S18. The HRESIMS spectrum of compound **2** S21
Figure S19. The ^{1}H NMR spectrum of compound **3** in CD_3OD S22
Figure S20. The ^{13}C NMR spectrum of compound **3** in CD_3OD S23
Figure S21. The COSY spectrum of compound **3** in CD_3OD S24
Figure S22. The HSQC spectrum of compound **3** in CD_3OD S25
Figure S23. The HMBC spectrum of compound **3** in CD_3OD S26
Figure S24. The NOESY spectrum of compound **3** in CD_3OD S27
Figure S25. The HRESIMS spectrum of compound **3** S28
Figure S26. The ^{1}H NMR chemical shift differences between **3 (***S*)- and **3 (***R***)**-MTPA esters S29
Figure S27. The HRESIMS spectrum of compound **3S** (**3R**) S30
Figure S28. The ^{1}H NMR spectrum of compound **4** in CD_3OD S31
Figure S29. The ^{13}C NMR spectrum of compound **4** in CD_3OD S32
Figure S30. The COSY spectrum of compound **4** in CD_3OD S33
Figure S31. The HSQC spectrum of compound **4** in CD_3OD S34
Figure S32. The HMBC spectrum of compound **4** in CD_3OD S35
Figure S33. The NOESY spectrum of compound **4** in CD_3OD S36
Figure S34. The HRESIMS spectrum of compound **4** S37
Figure S35. The ^{1}H NMR chemical shift differences between **4 (***S*)- and **4 (***R***)**-MTPA esters S38
Figure S36. The HRESIMS spectrum of compound **4S** (**4R**) S39
Figure S37. The ^{1}H NMR spectrum of compound **5** in CD_3OD S40
Figure S38. The ^{13}C NMR spectrum of compound **5** in CD_3OD S41
Figure S39. The COSY spectrum of compound **5** in CD_3OD S42
Figure S40. The HSQC spectrum of compound **5** in CD_3OD S43
Figure S41. The HMBC spectrum of compound **5** in CD_3OD S44
Figure S42. The NOESY spectrum of compound **5** in CD_3OD S45
Figure S43. The HRESIMS spectrum of compound **5** S46
Figure S44. The ^{1}H NMR spectrum of compound **6** in CD_3OD S47
Figure S45. The ^{13}C NMR spectrum of compound **6** in CD_3OD S48
Figure S46. The COSY spectrum of compound **6** in CD_3OD S49
Figure S47. The HSQC spectrum of compound **6** in CD_3OD S50

Figure S48. The HMBC spectrum of compound **6** in CD_3OD S51
Figure S49. The NOESY spectrum of compound **6** in CD_3OD S52
Figure S50. The HRESIMS spectrum of compound **6** S53
Figure S51. The ^1H NMR spectrum of compound **7** in CD_3OD S54
Figure S52. The ^{13}C NMR spectrum of compound **7** in CD_3OD S55
Figure S53. The COSY spectrum of compound **7** in CD_3OD S56
Figure S54. The HSQC spectrum of compound **7** in CD_3OD S57
Figure S55. The HMBC spectrum of compound **7** in CD_3OD S58
Figure S56. The NOESY spectrum of compound **7** in CD_3OD S59
Figure S57. The HRESIMS spectrum of compound **7** S60
Figure S58. Photo of the sponge *Haliclona gracilis* S61

Figure S1. The ^{1}H NMR (700 MHz, CD_3OD) spectrum of compound **1**

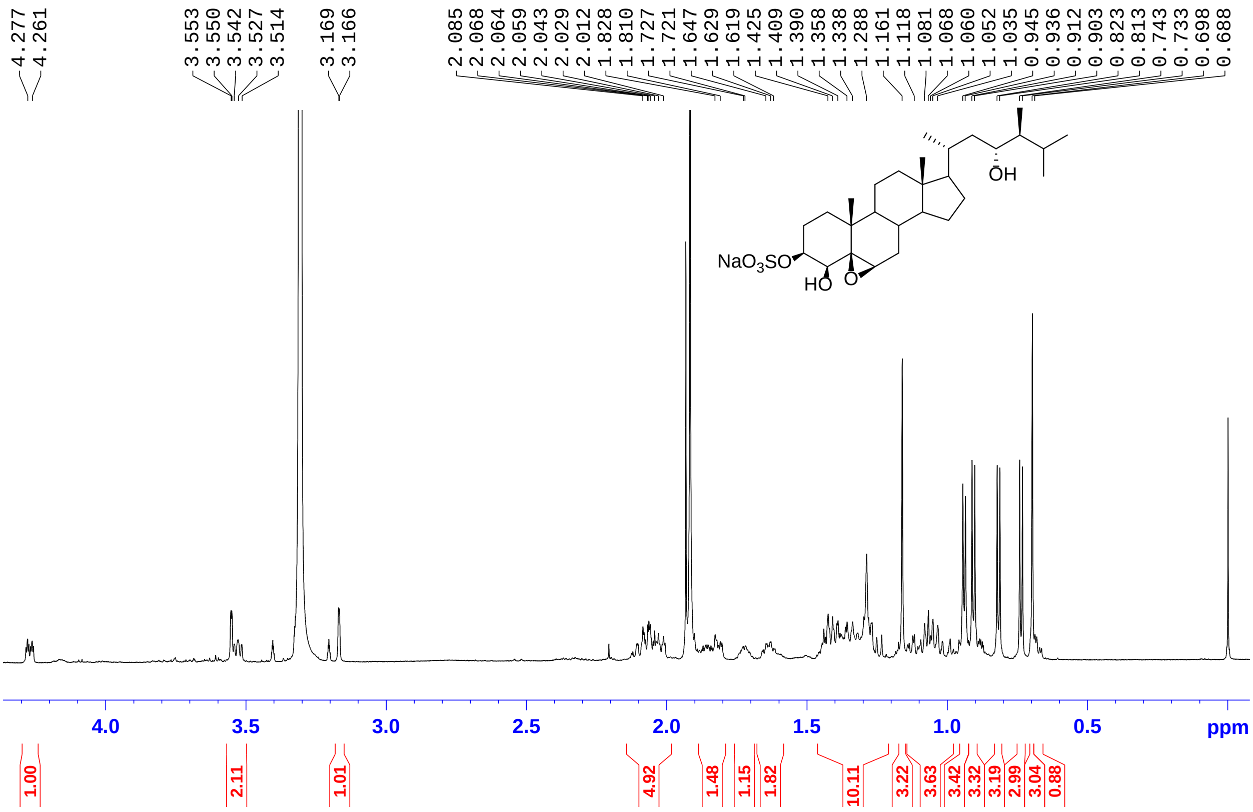

Figure S2. The ^{13}C NMR(125 MHz, CD_3OD) spectrum of compound **1**

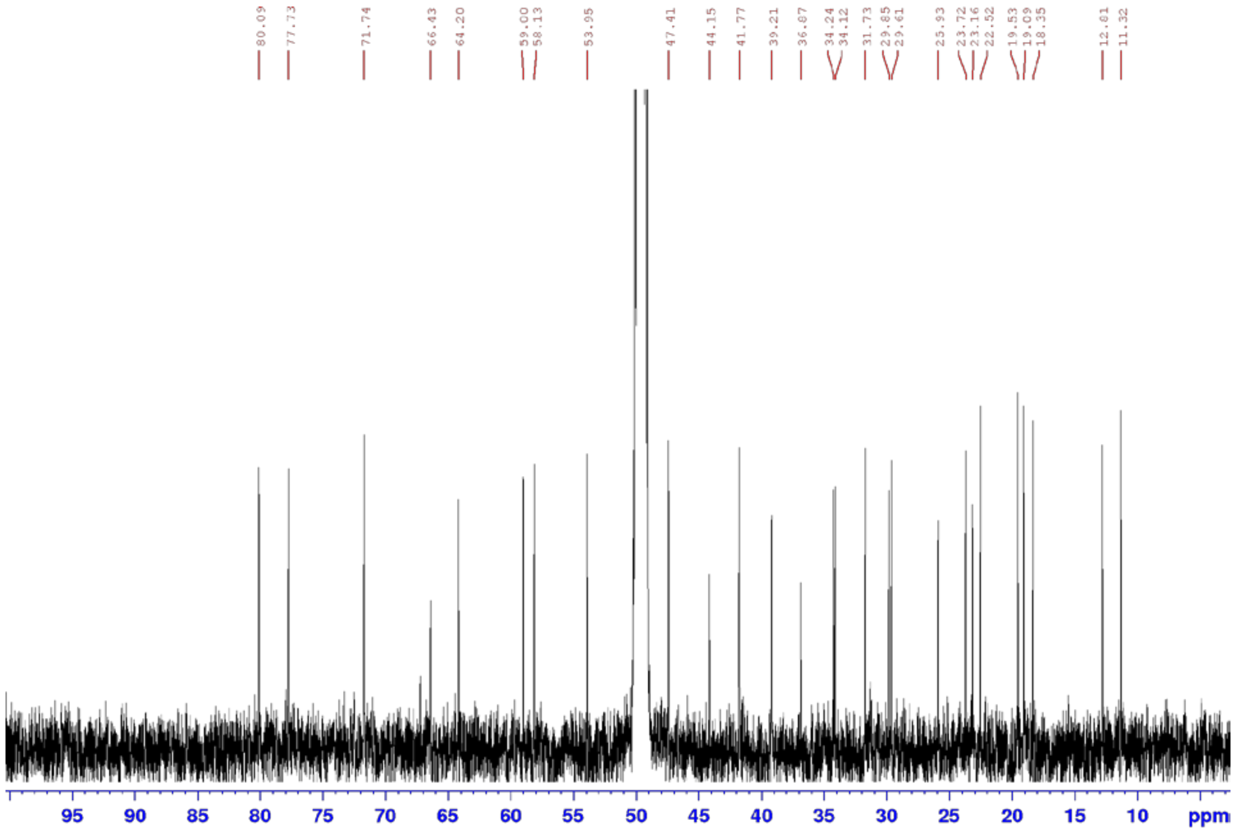

Figure S3. The COSY (700 MHz, CD_3OD) spectrum of compound **1**

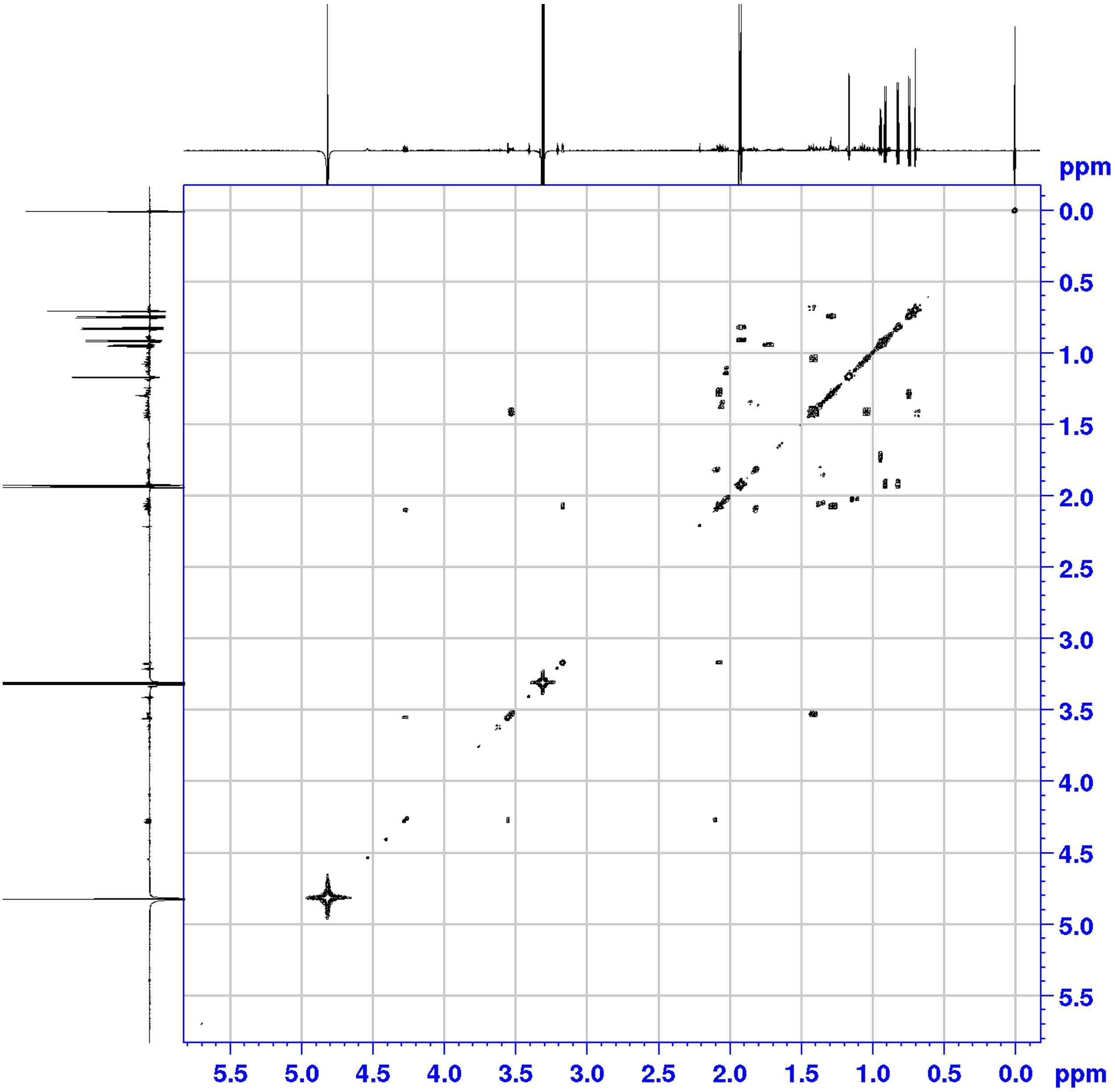

Figure S4. The HSQC (500/125 MHz, CD_3OD) spectrum of compound **1**

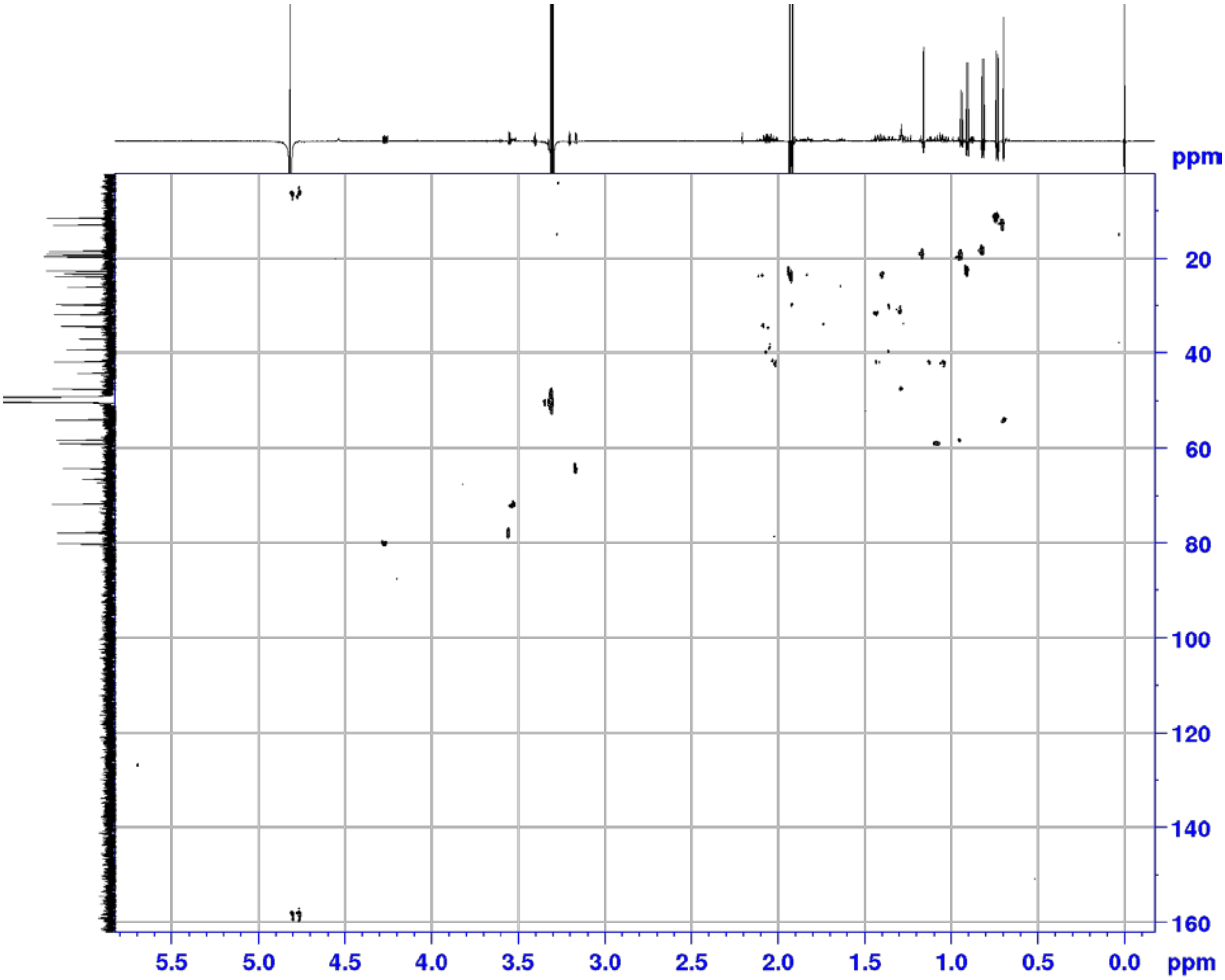

Figure S5. The HMBC (500/125 MHz, DMSO-d_6) spectrum of compound **1**

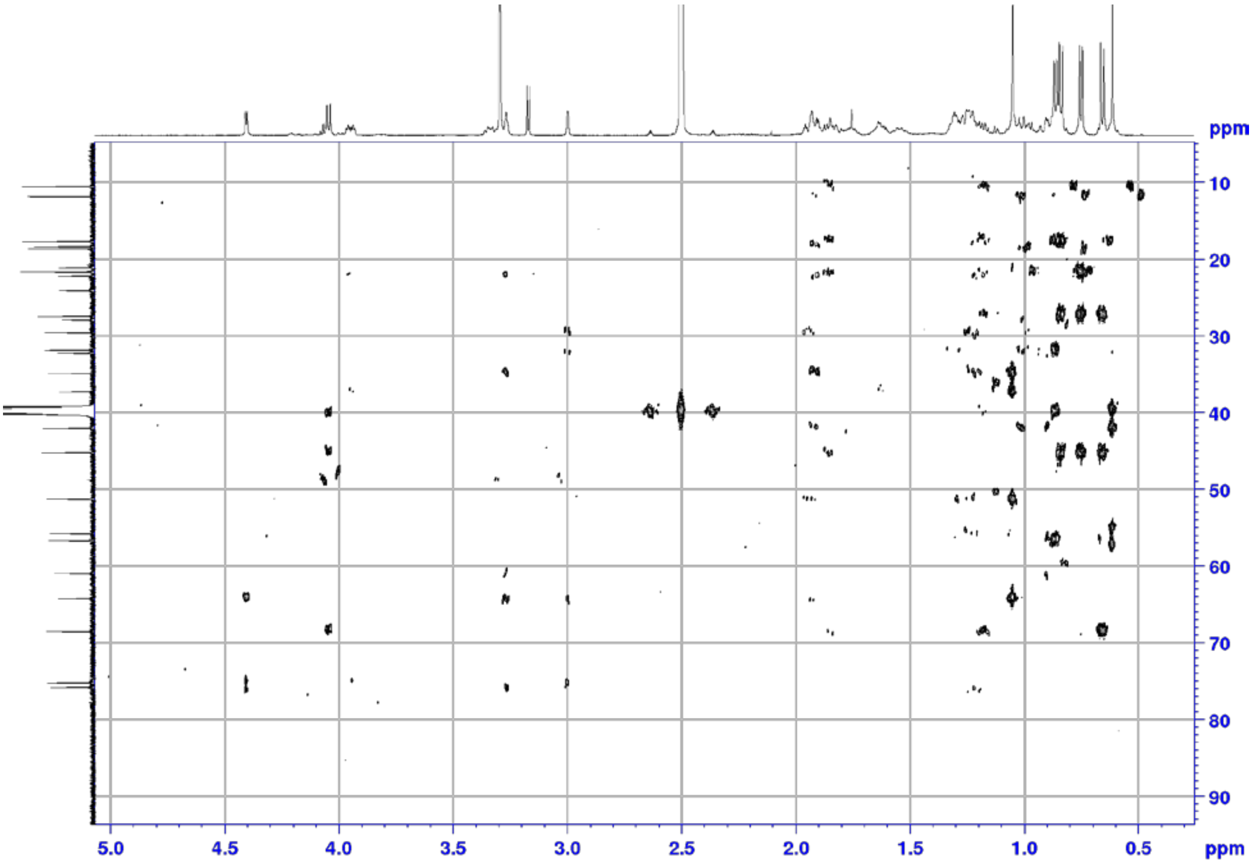

Figure S6. The NOESY (700 MHz, CD_3OD) spectrum of compound **1**

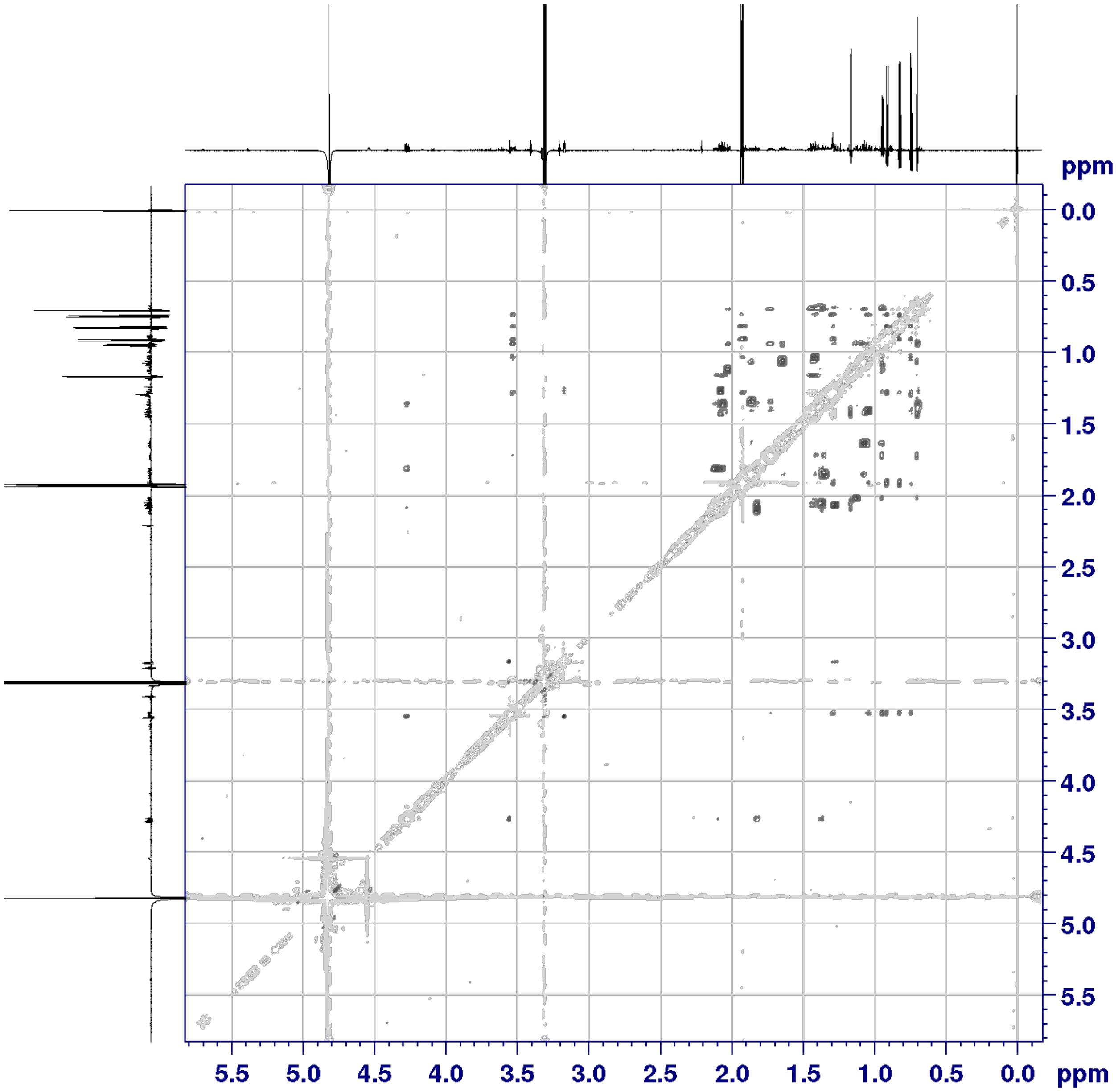

Figure S7. The HRESIMS and MS/MS spectra of compound **1**

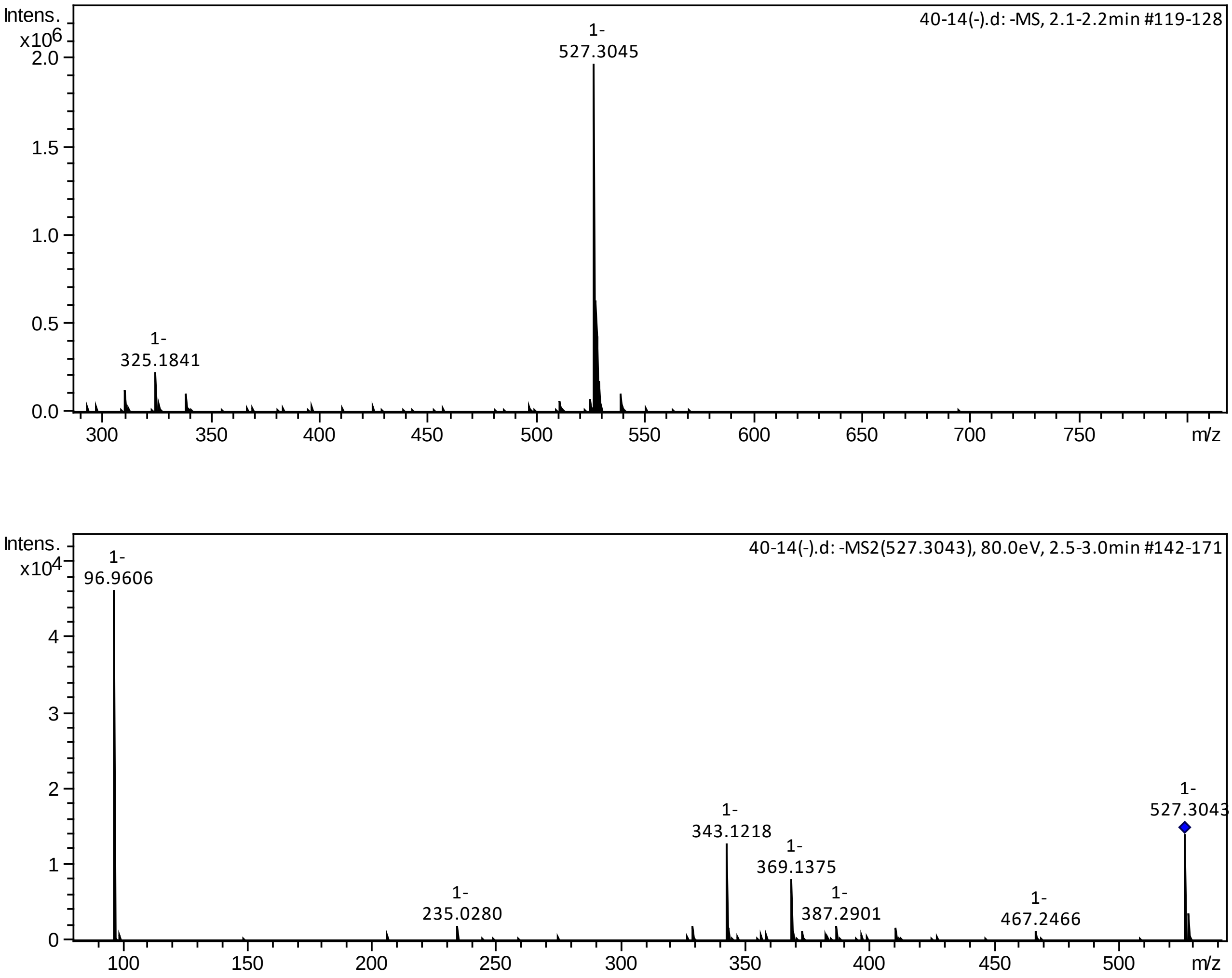

Figure S8. The ^{1}H NMR (700 MHz, CD_3OD) chemical shift differences between **1S** (red) and **1R** (blue)

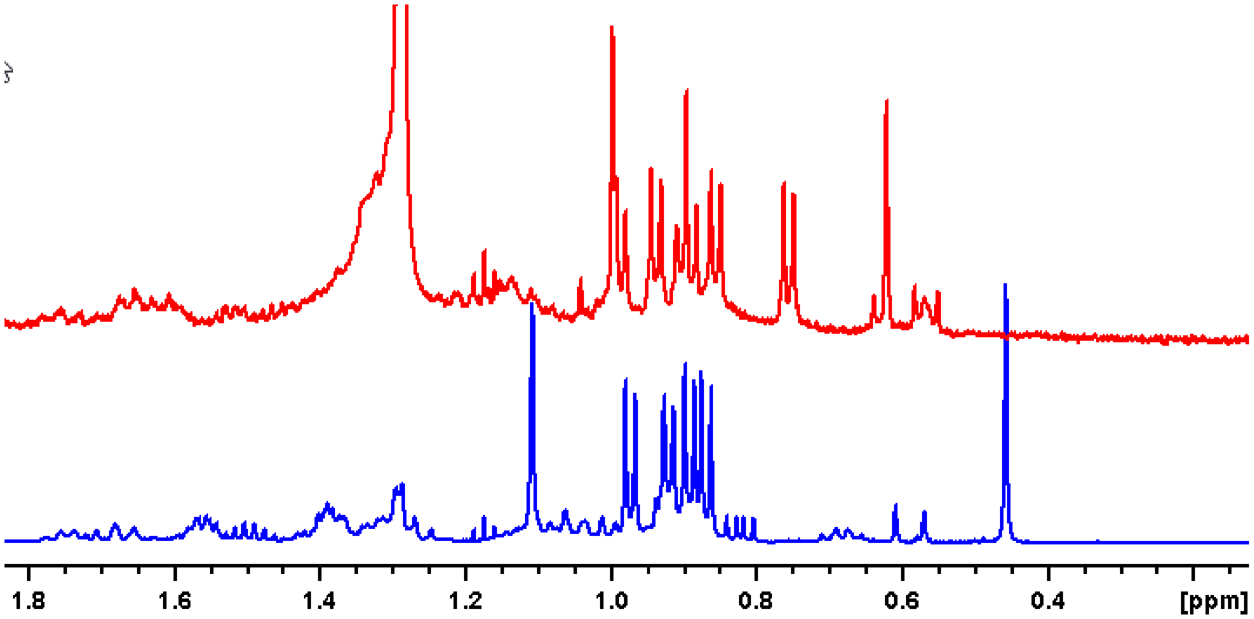

Figure S9. The HRESIMS spectrum of compound **1S** (**1R**)

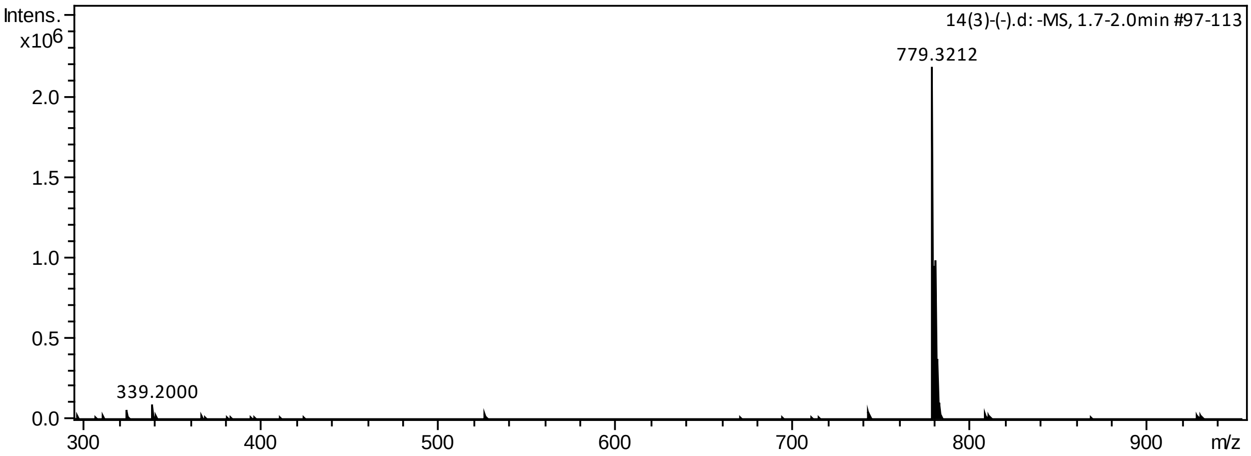

Figure S10. The ^{1}H NMR (700 MHz, DMSO-d_6) spectrum of compound **1**

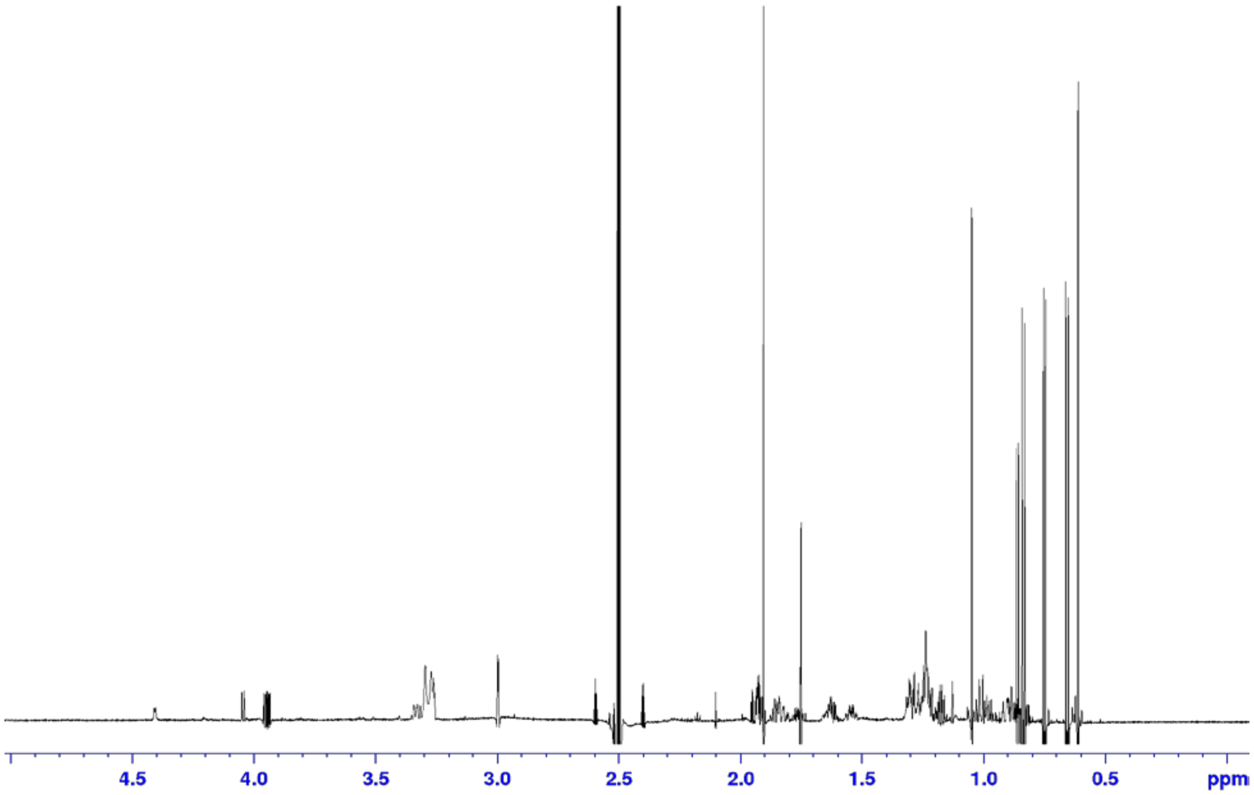

Figure S11. The COSY (700 MHz, DMSO-d_6) spectrum of compound **1**

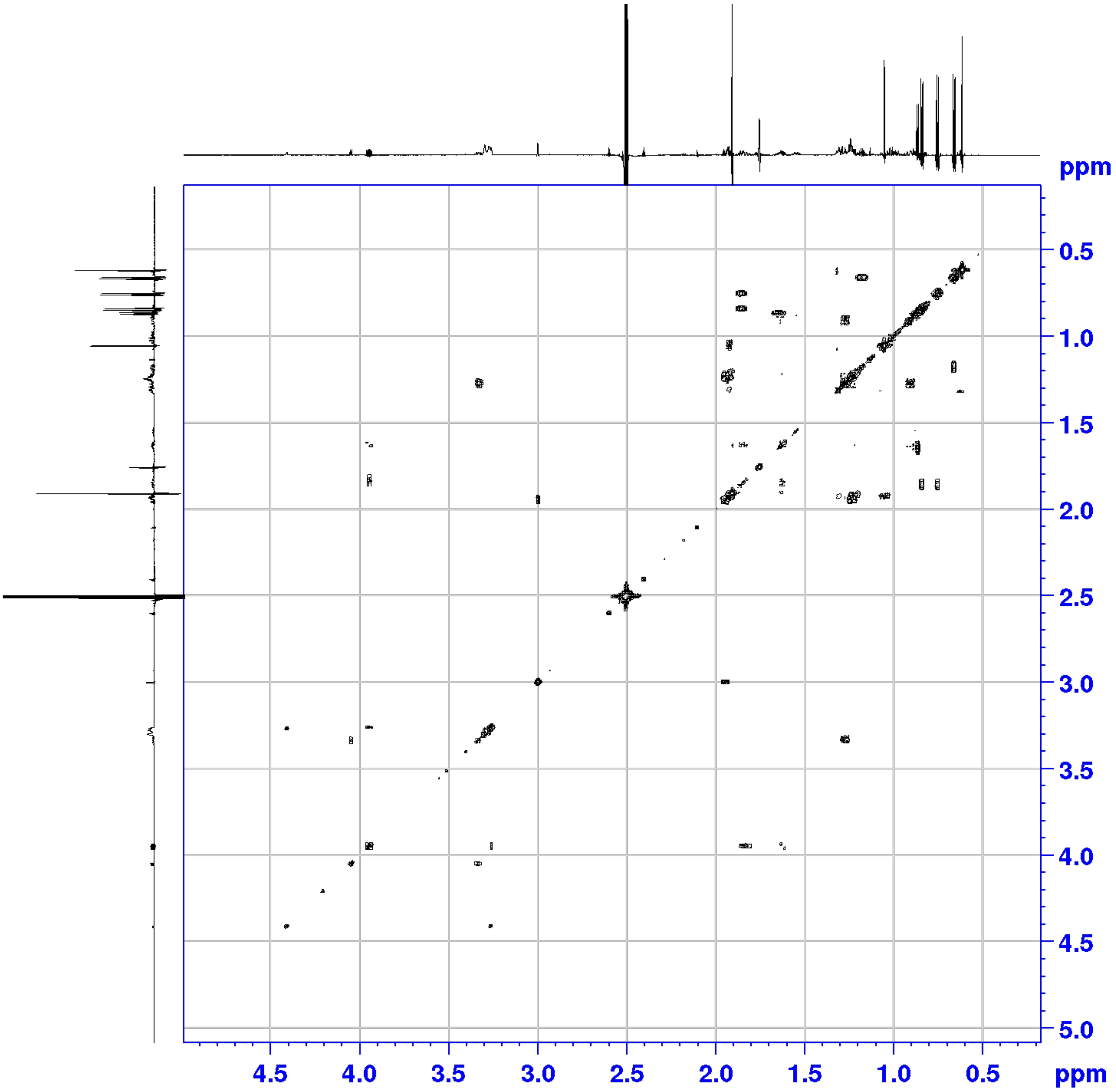

Figure S12. The ^{1}H NMR (500 MHz, CD_3OD) spectrum of compound **2**

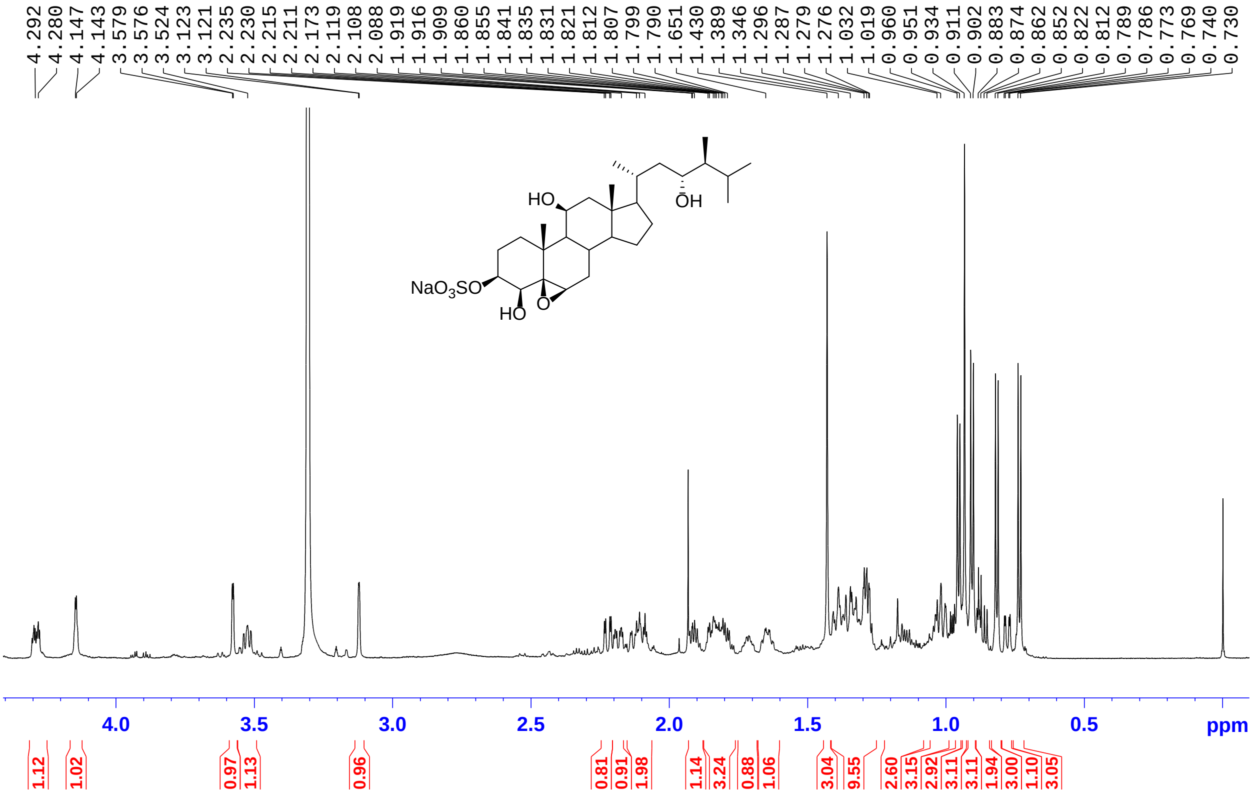

Figure S13. The ^{13}C NMR (125 MHz, CD_3OD) spectrum of compound **2**

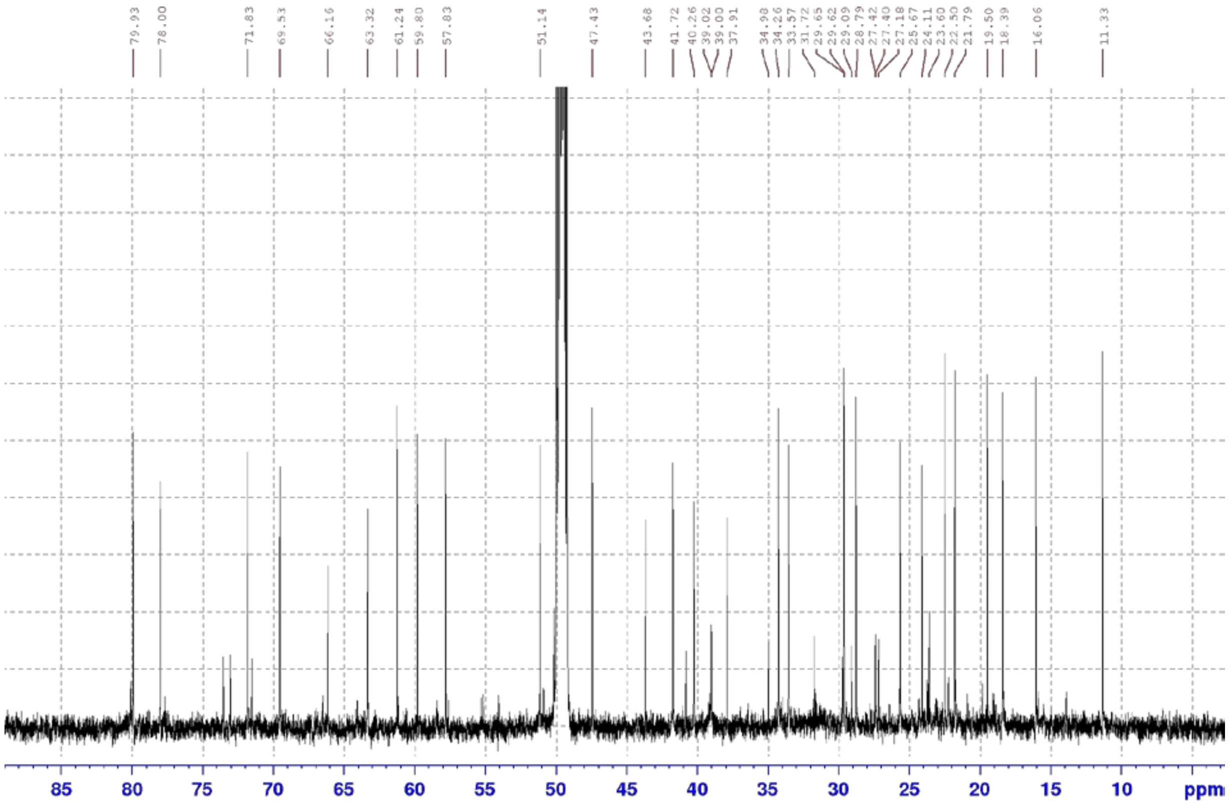

Figure S14. The COSY (500 MHz, CD_3OD) spectrum of compound **2**

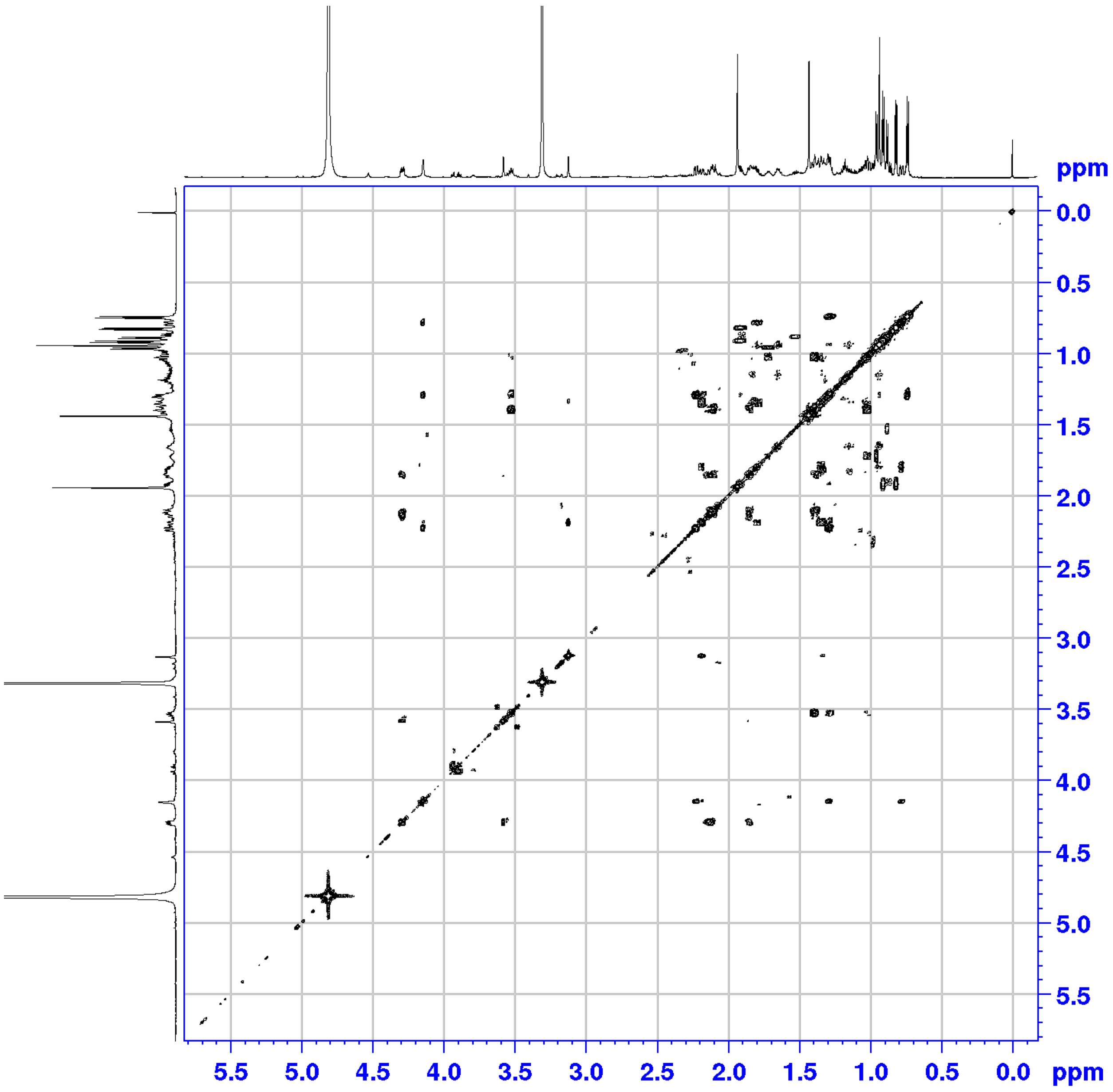

Figure S15. The HSQC (500/125 MHz, CD_3OD) spectrum of compound **2**

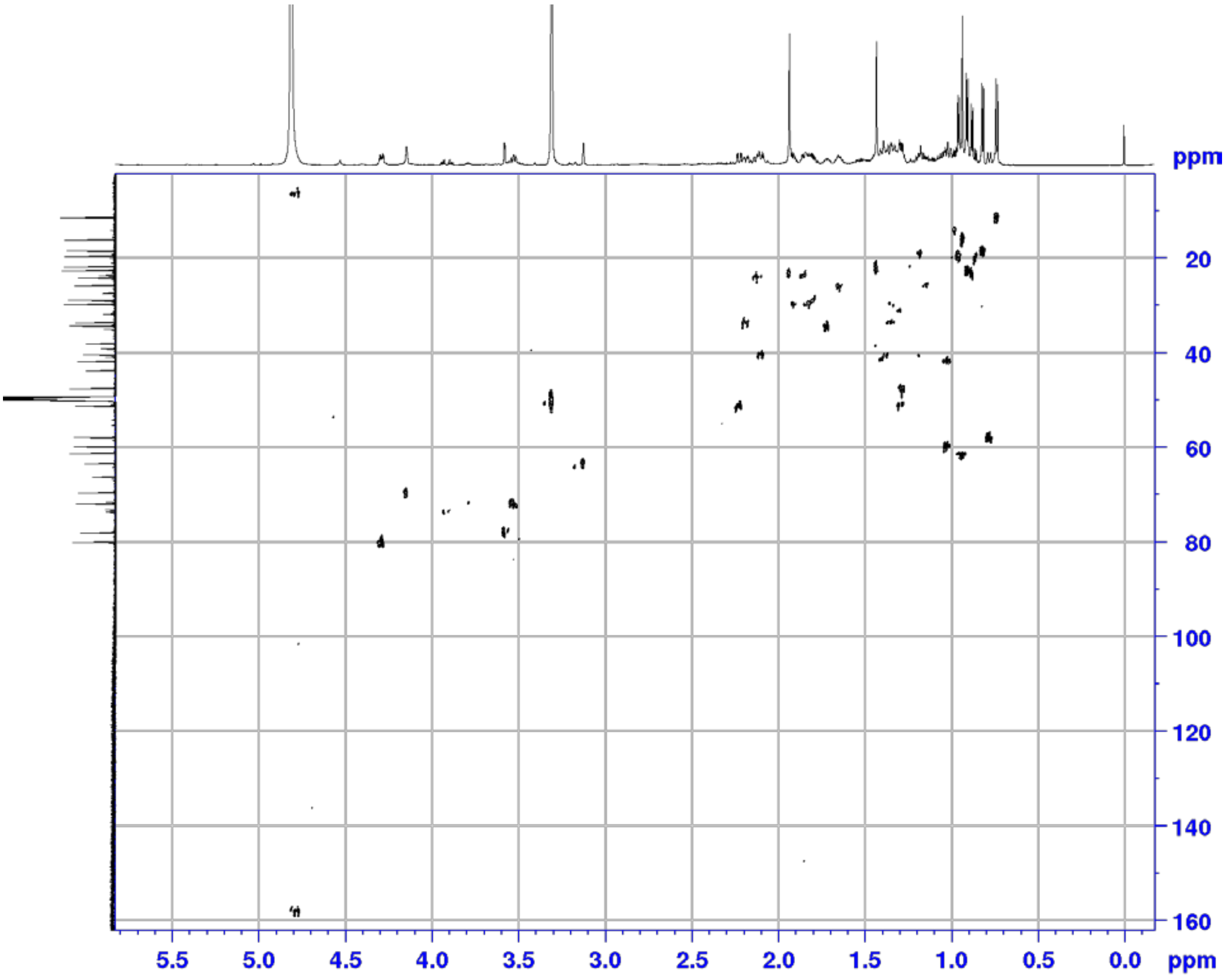

Figure S16. The HMBC (500/125 MHz, CD_3OD) spectrum of compound **2**

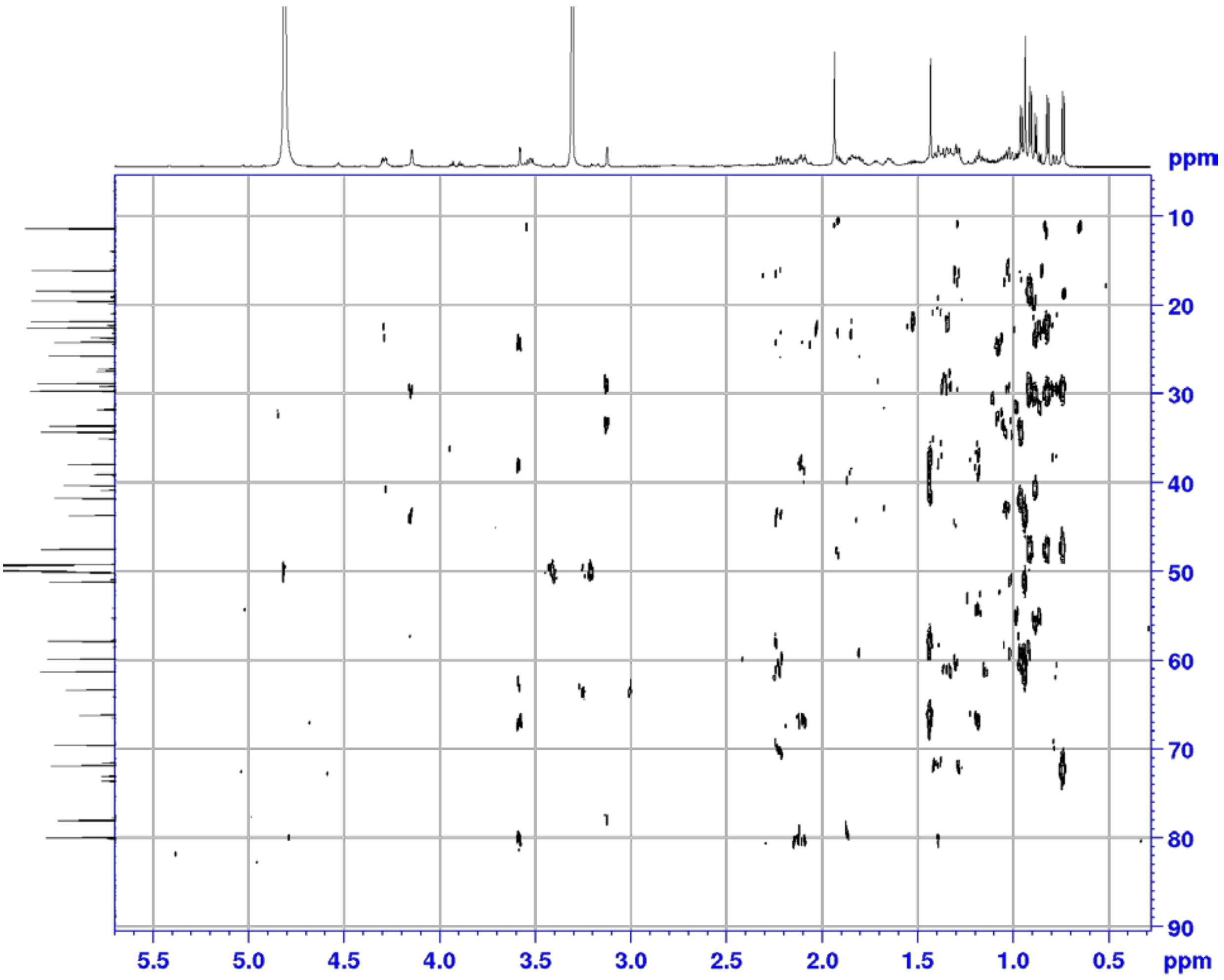

Figure S17. The NOESY (700 MHz, CD_3OD) spectrum of compound **2**

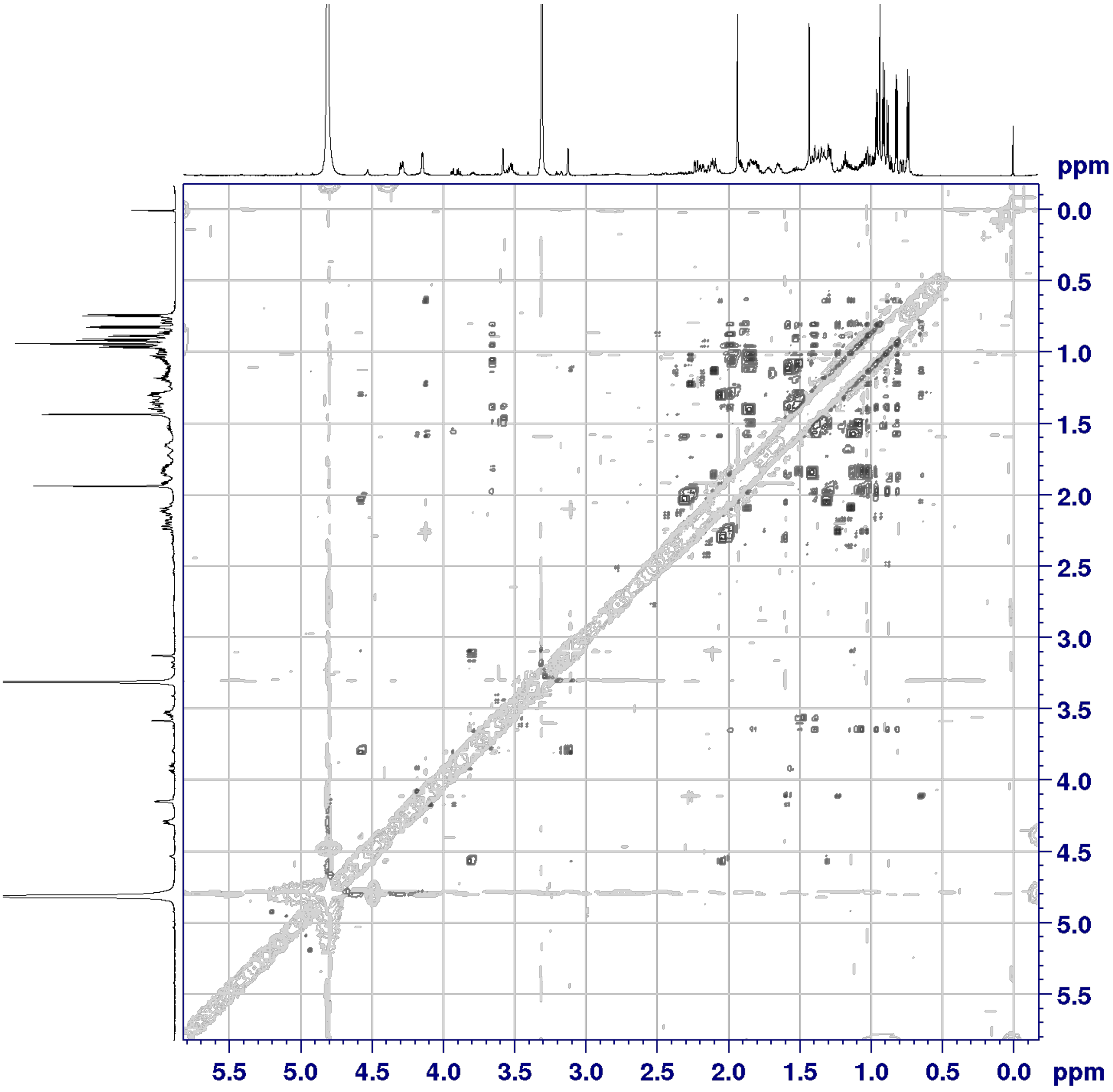

Figure S18. The HRESIMS spectrum of compound **2**

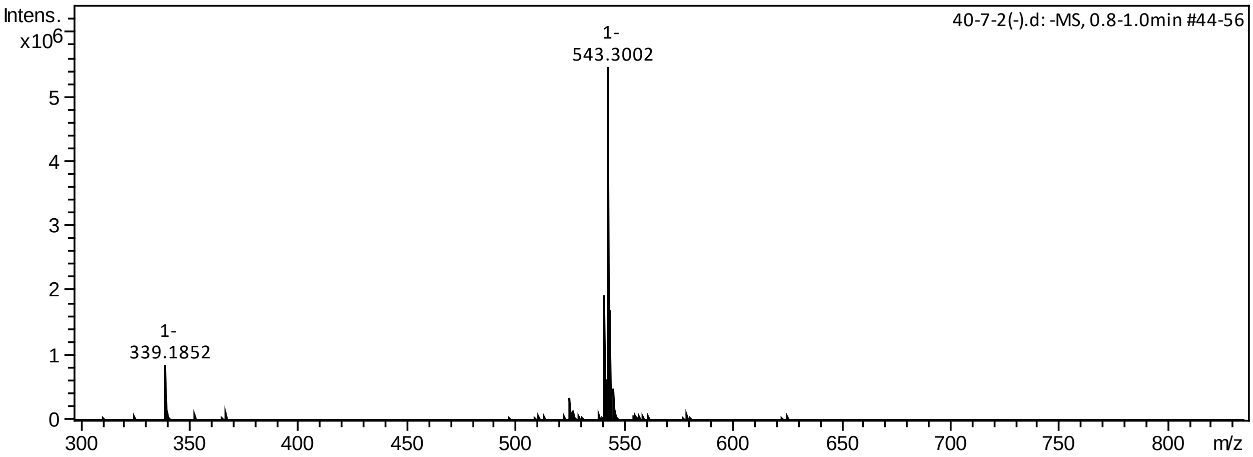

Figure S19. The ^{1}H (700 MHz, CD_3OD) spectrum of compound **3**

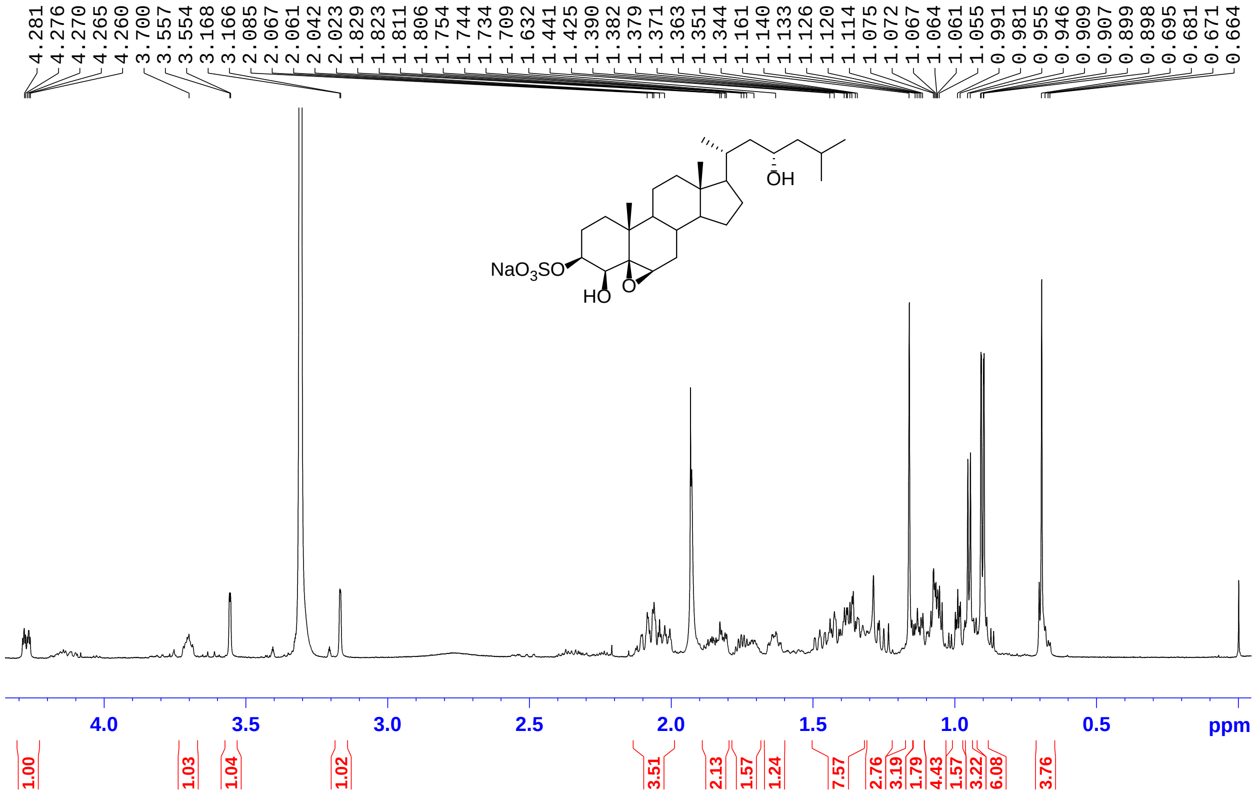

Figure S20. The ^{13}C (175 MHz, CD_3OD) spectrum of compound **3**

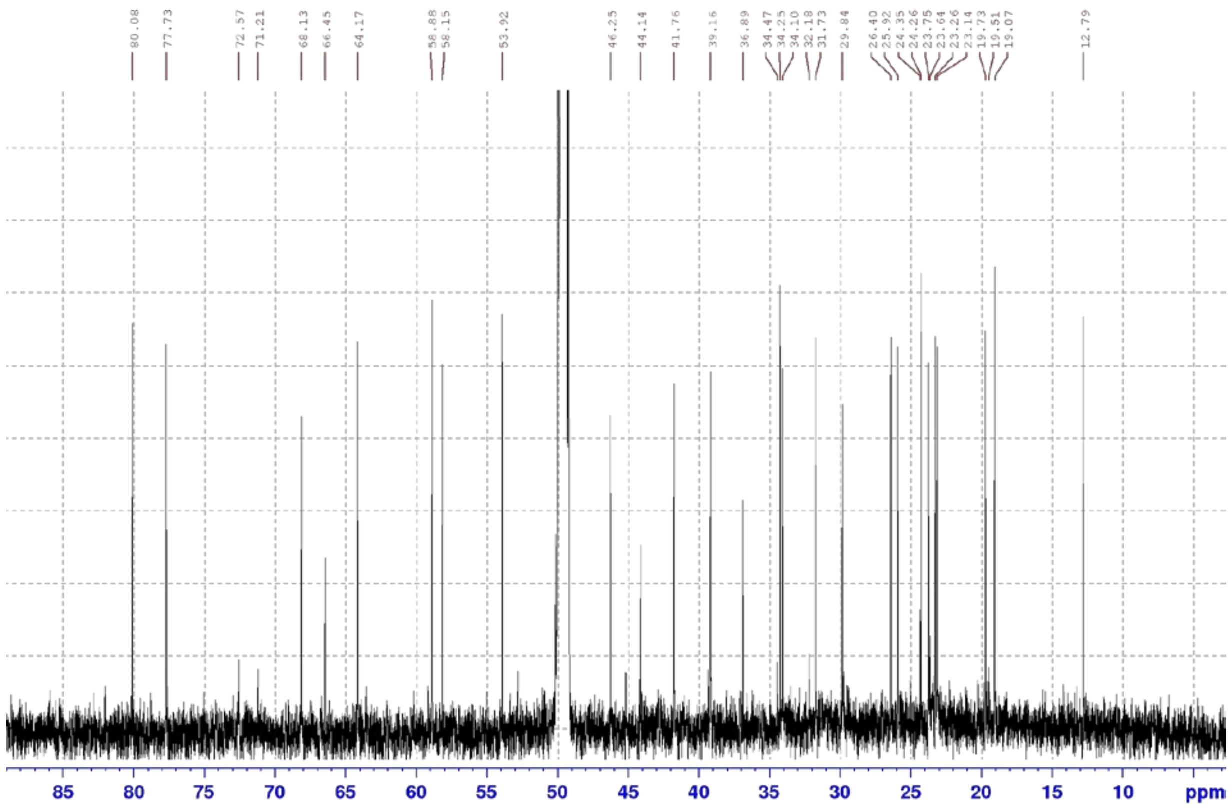

Figure S21. The COSY (700 MHz, CD_3OD) spectrum of compound **3**

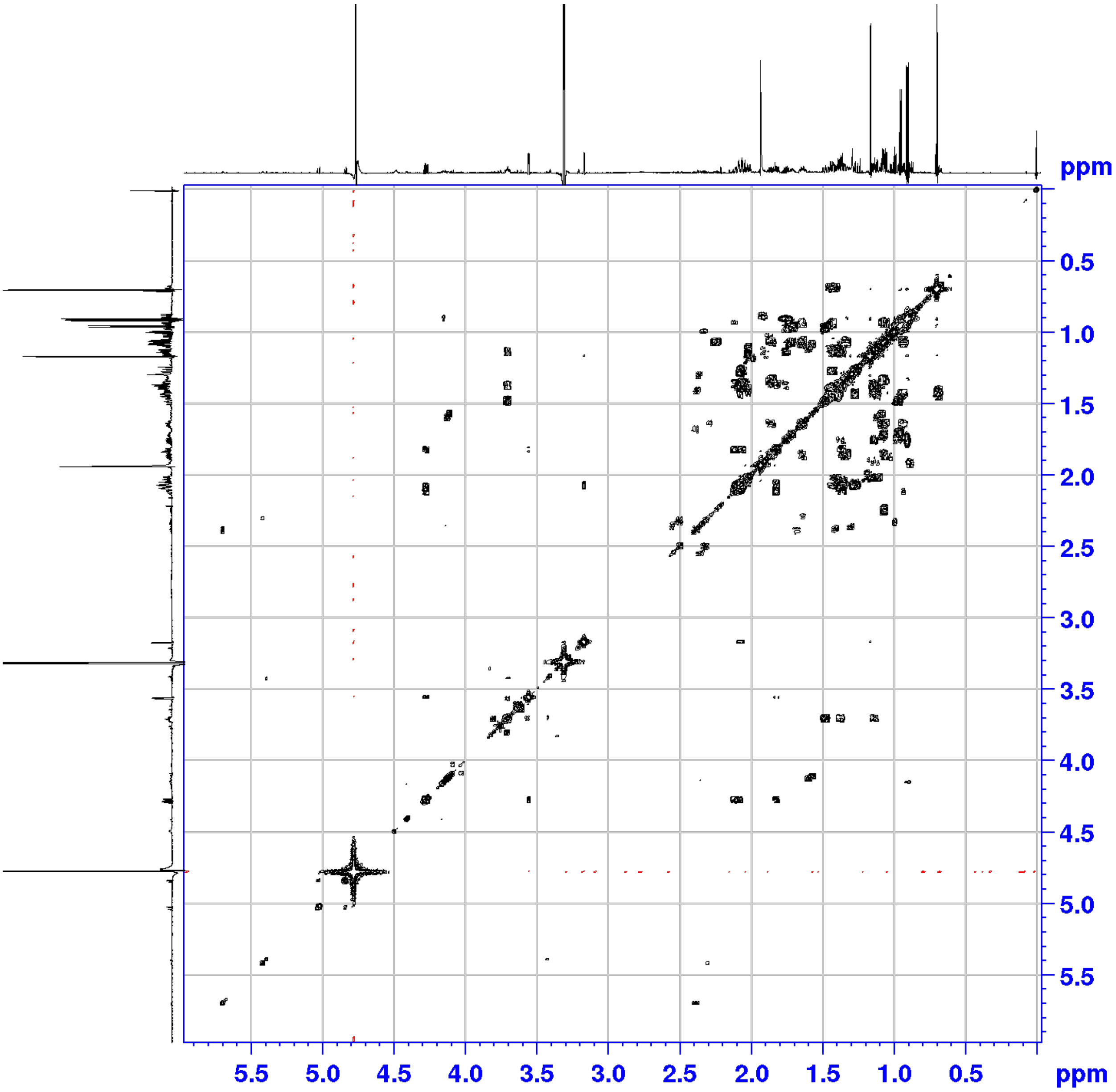

Figure S22. The HSQC (700/175 MHz, CD_3OD) spectrum of compound **3**

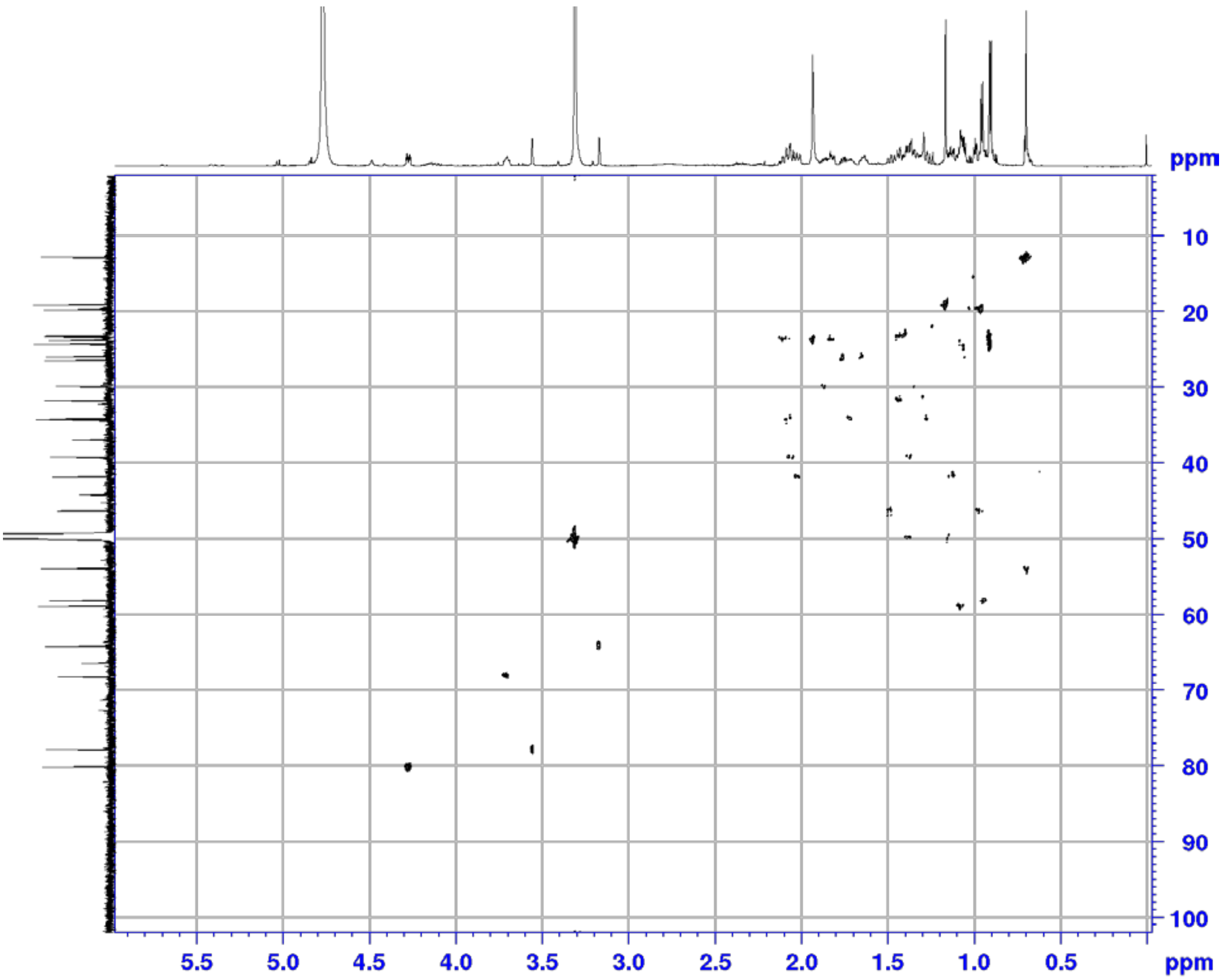

Figure S23. The HMBC (700/175 MHz, CD_3OD) spectrum of compound **3**

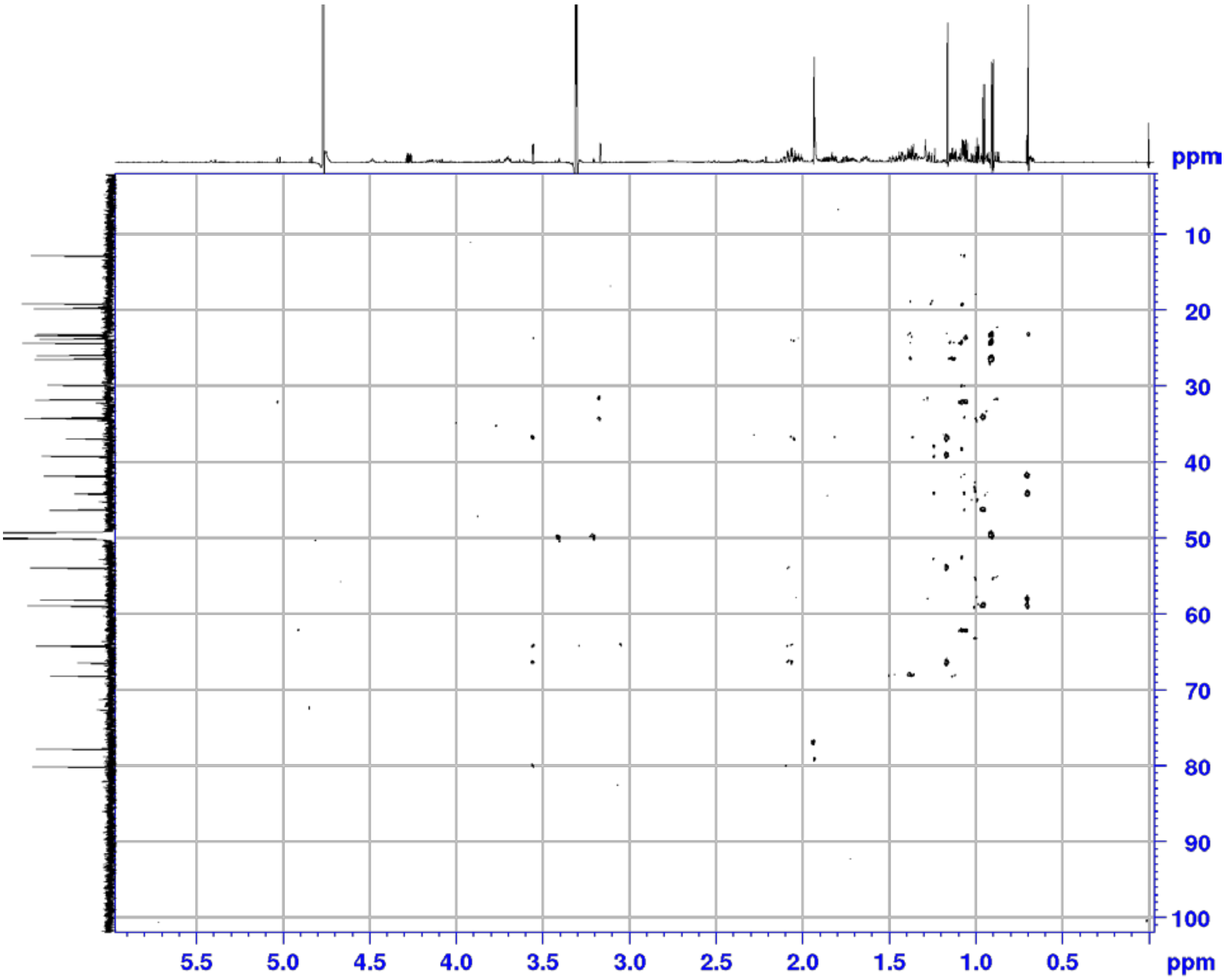

Figure S24. The NOESY (700 MHz, CD_3OD) spectrum of compound **3**

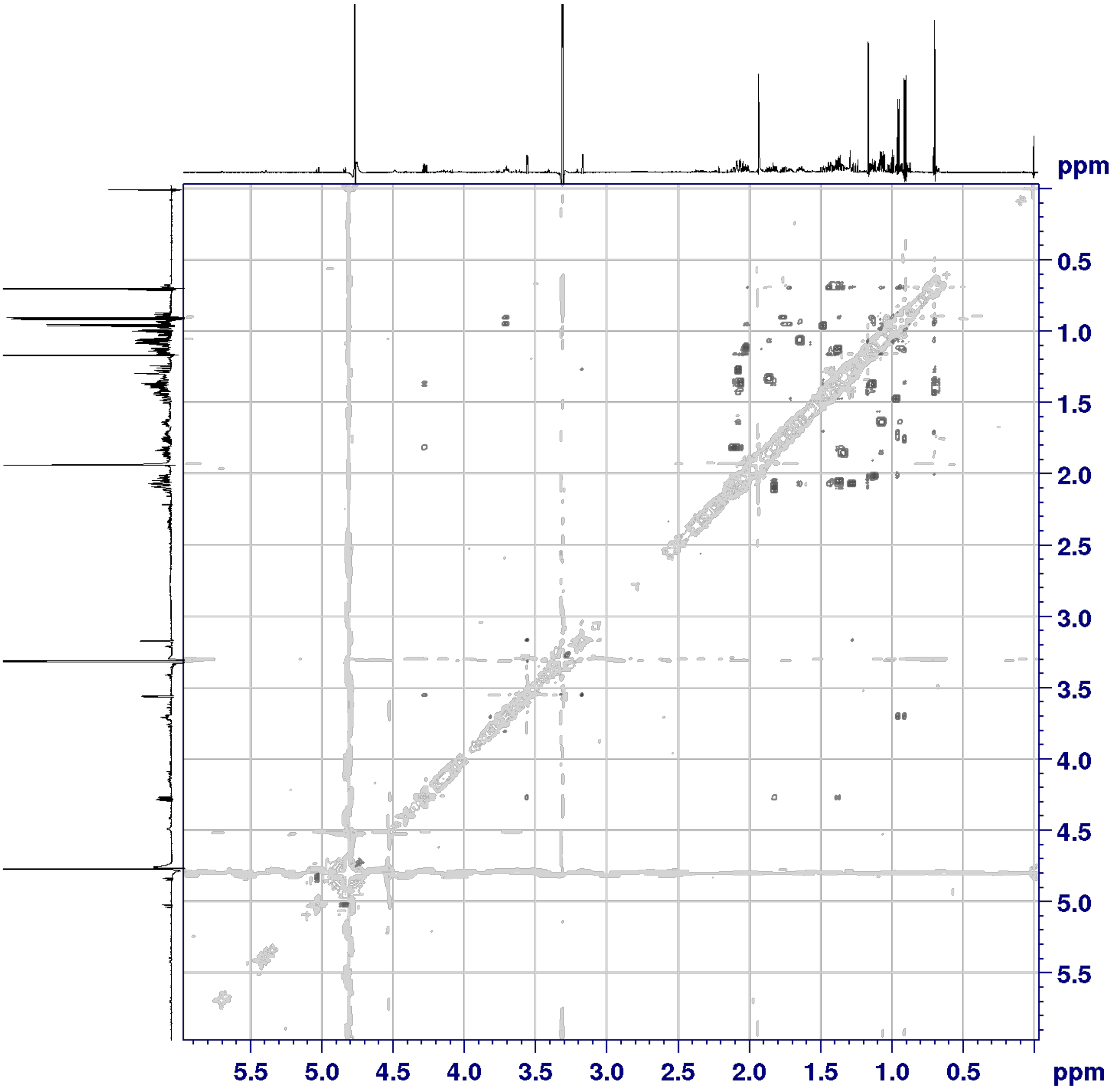

Figure S25. The HRESIMS spectrum of compound **3**

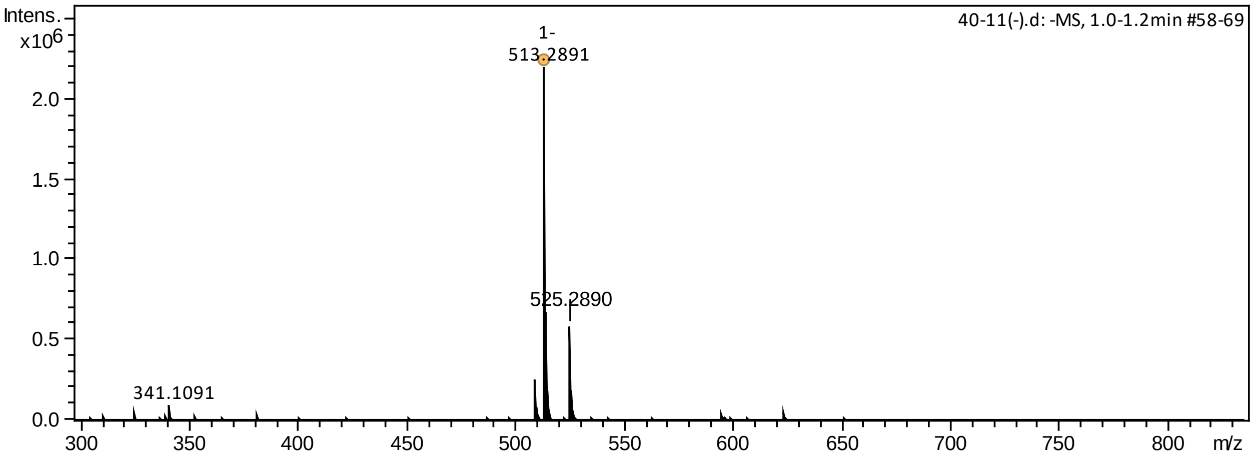

Figure S26. ^{1}H NMR chemical shift differences between **3S** (red) and **3R** (blue)

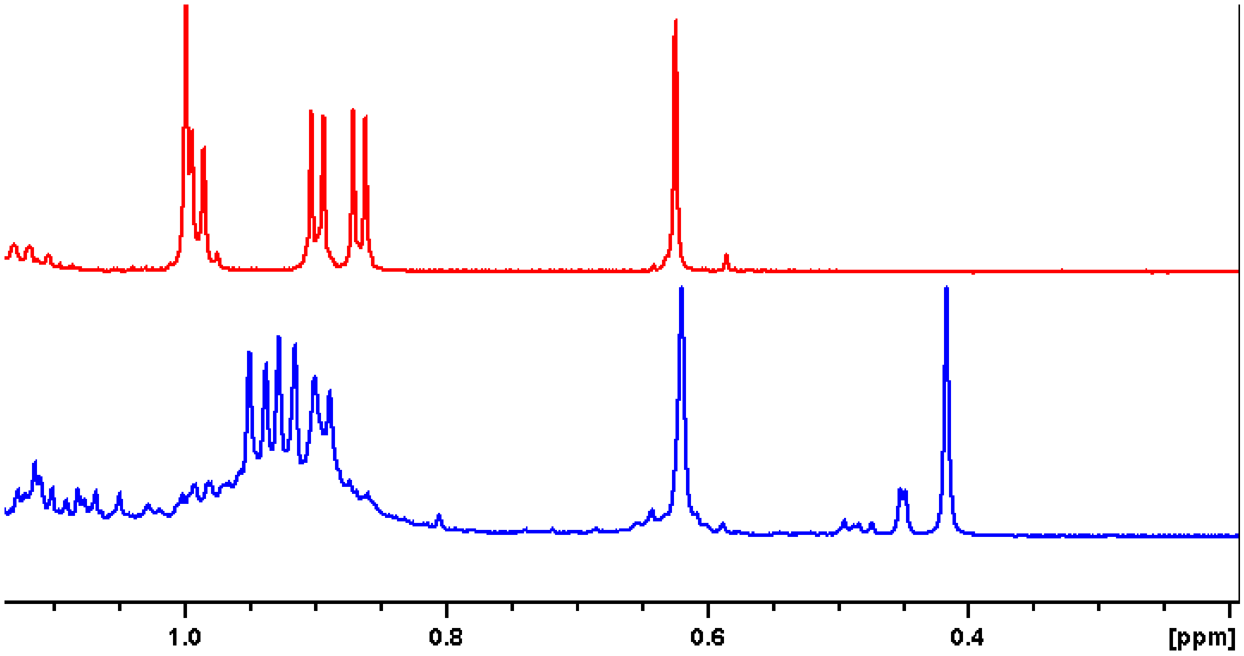

Figure S27. The HRESIMS spectrum of compound **3R** (**3S**)

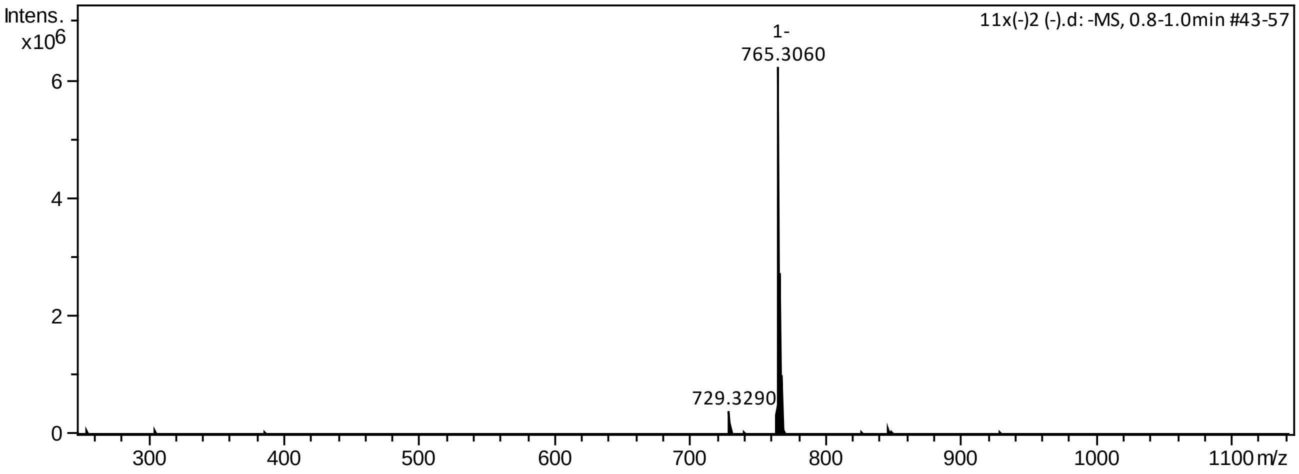

Figure S28. The ^{1}H (700 MHz, CD_3OD) spectrum of compound **4**

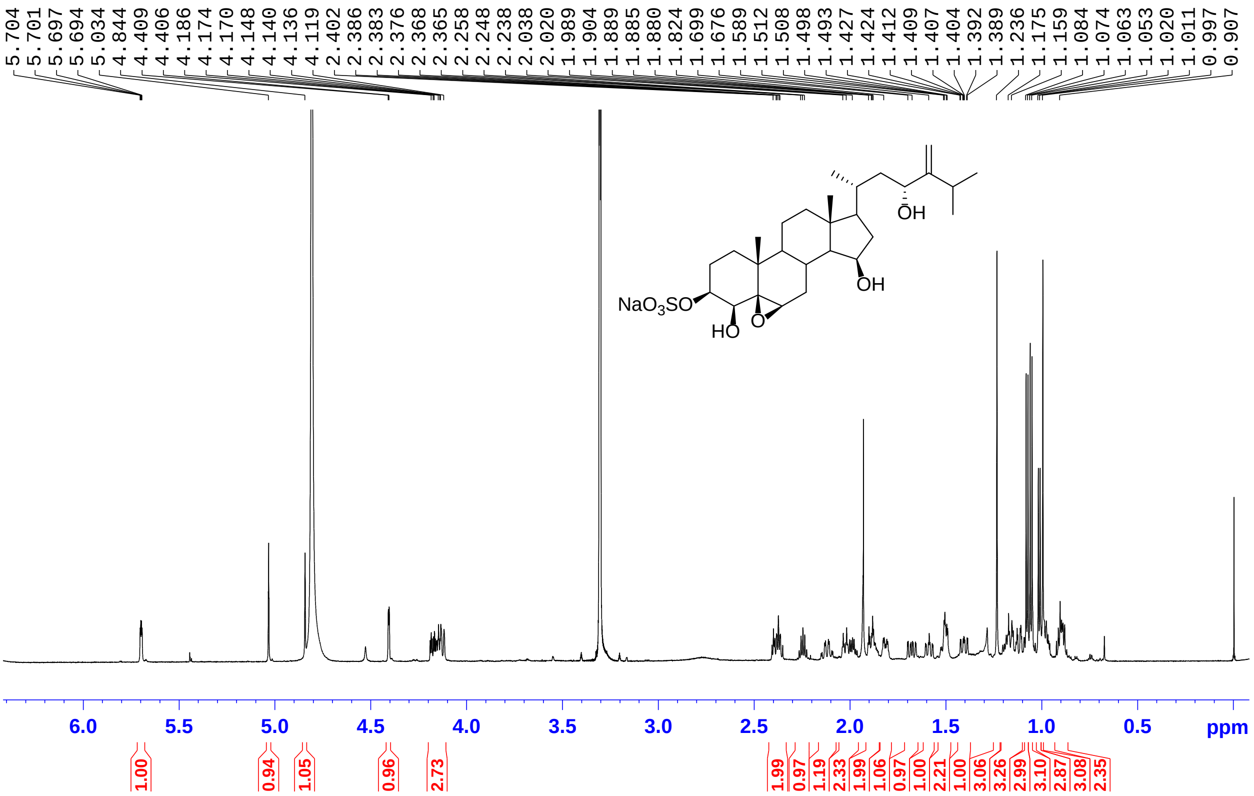

Figure S29. The ^{13}C (175 MHz, CD_3OD) spectrum of compound **4**

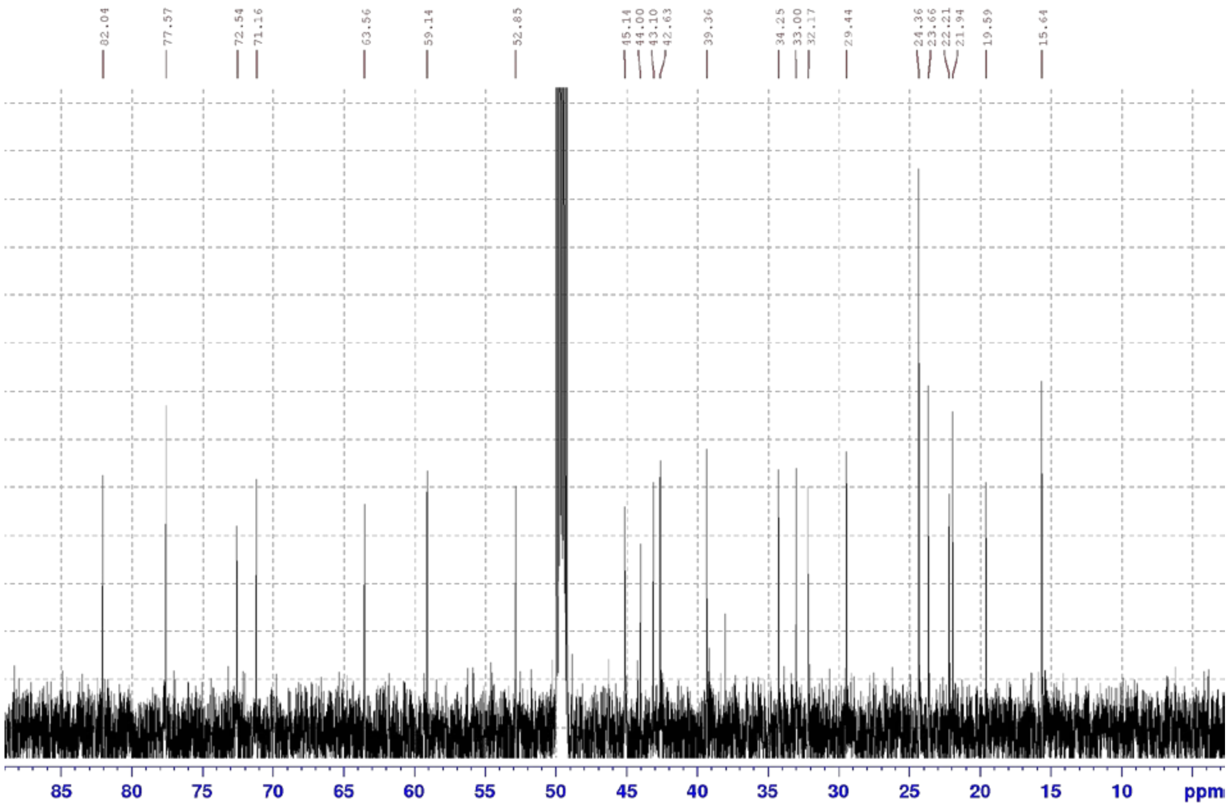

Figure S30. The COSY (700 MHz, CD_3OD) spectrum of compound **4**

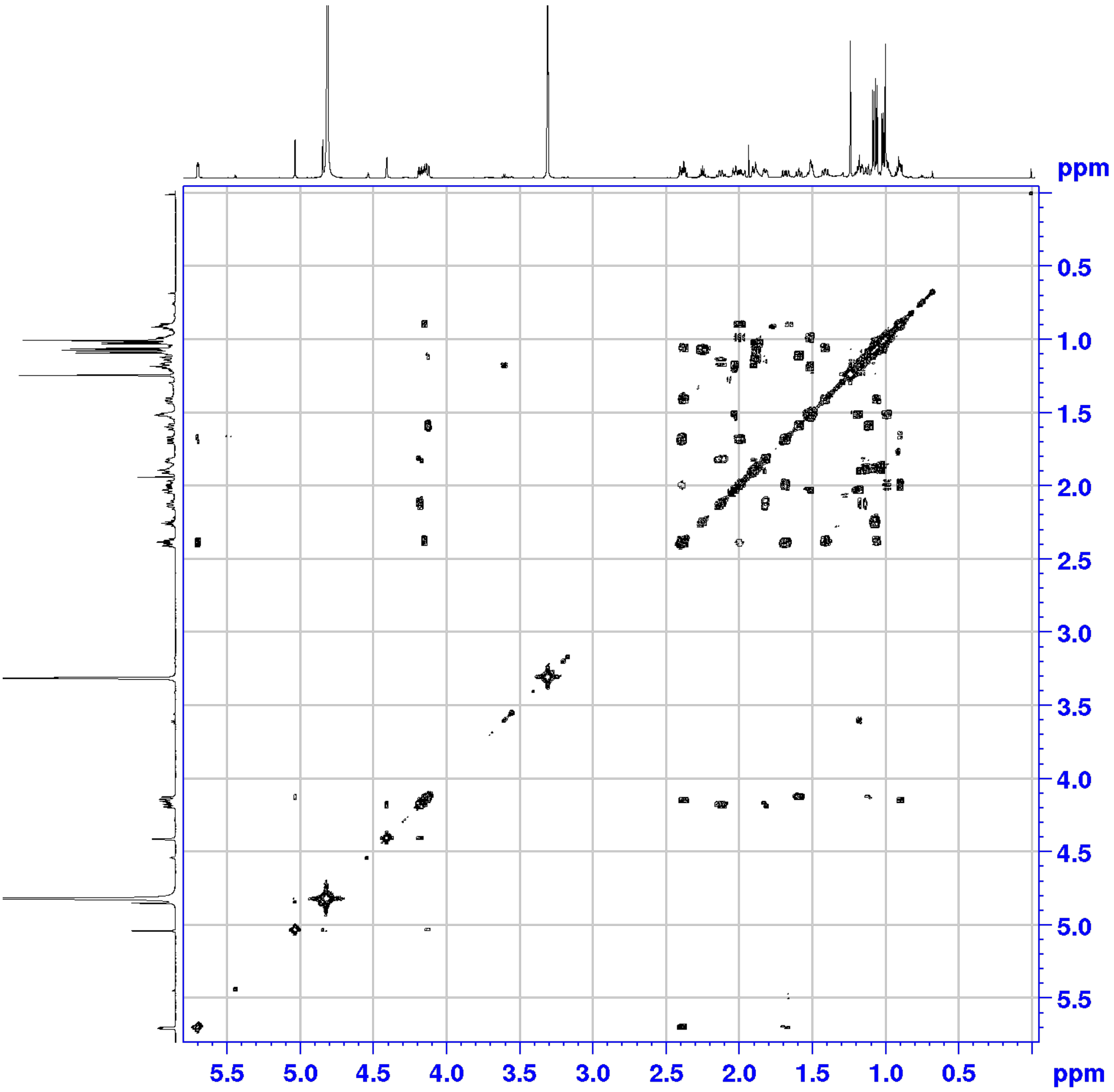

Figure S31. The HSQC (700/175 MHz, CD_3OD) spectrum of compound **4**

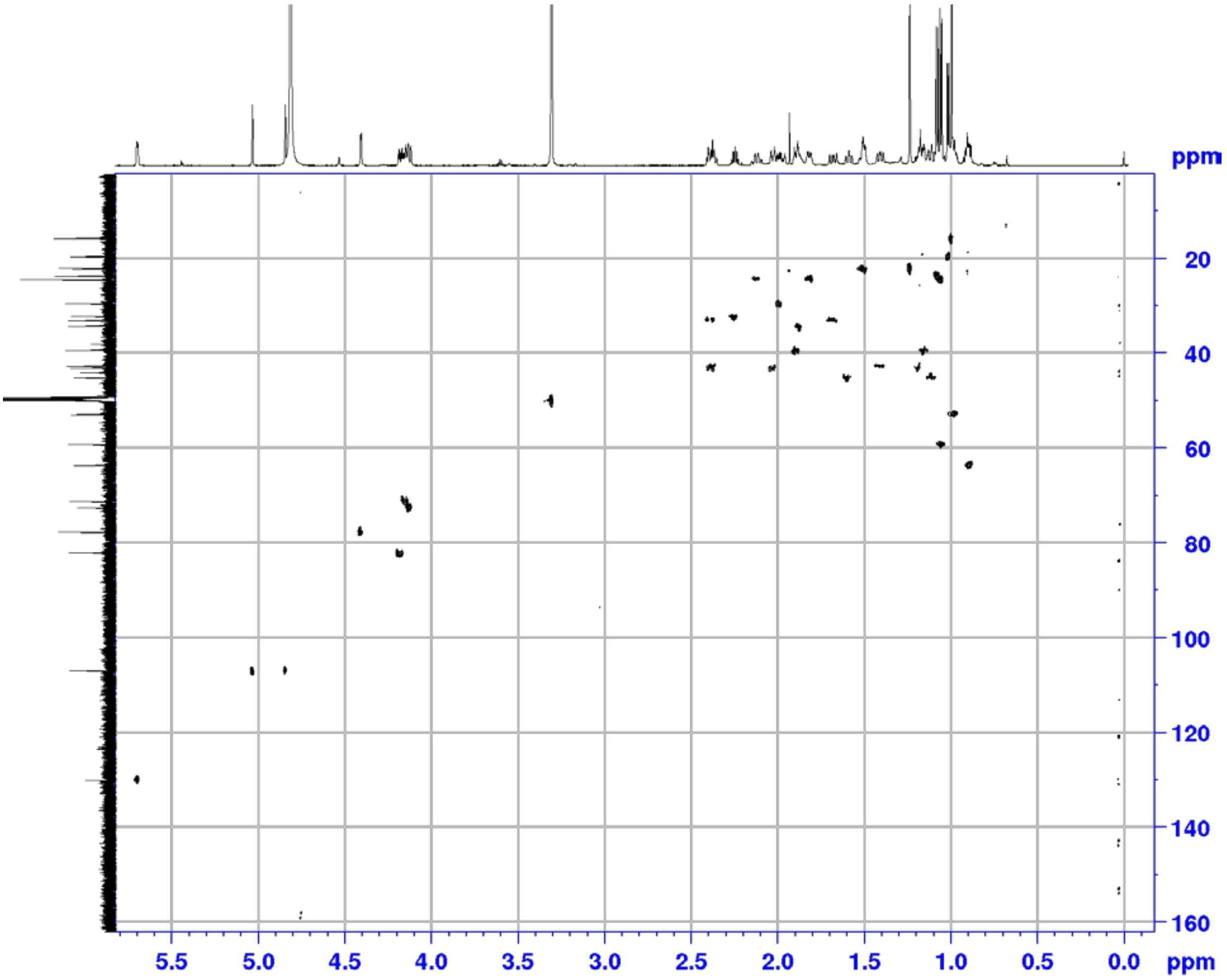

Figure S32. The HMBC (700/175 MHz, CD_3OD) spectrum of compound **4**

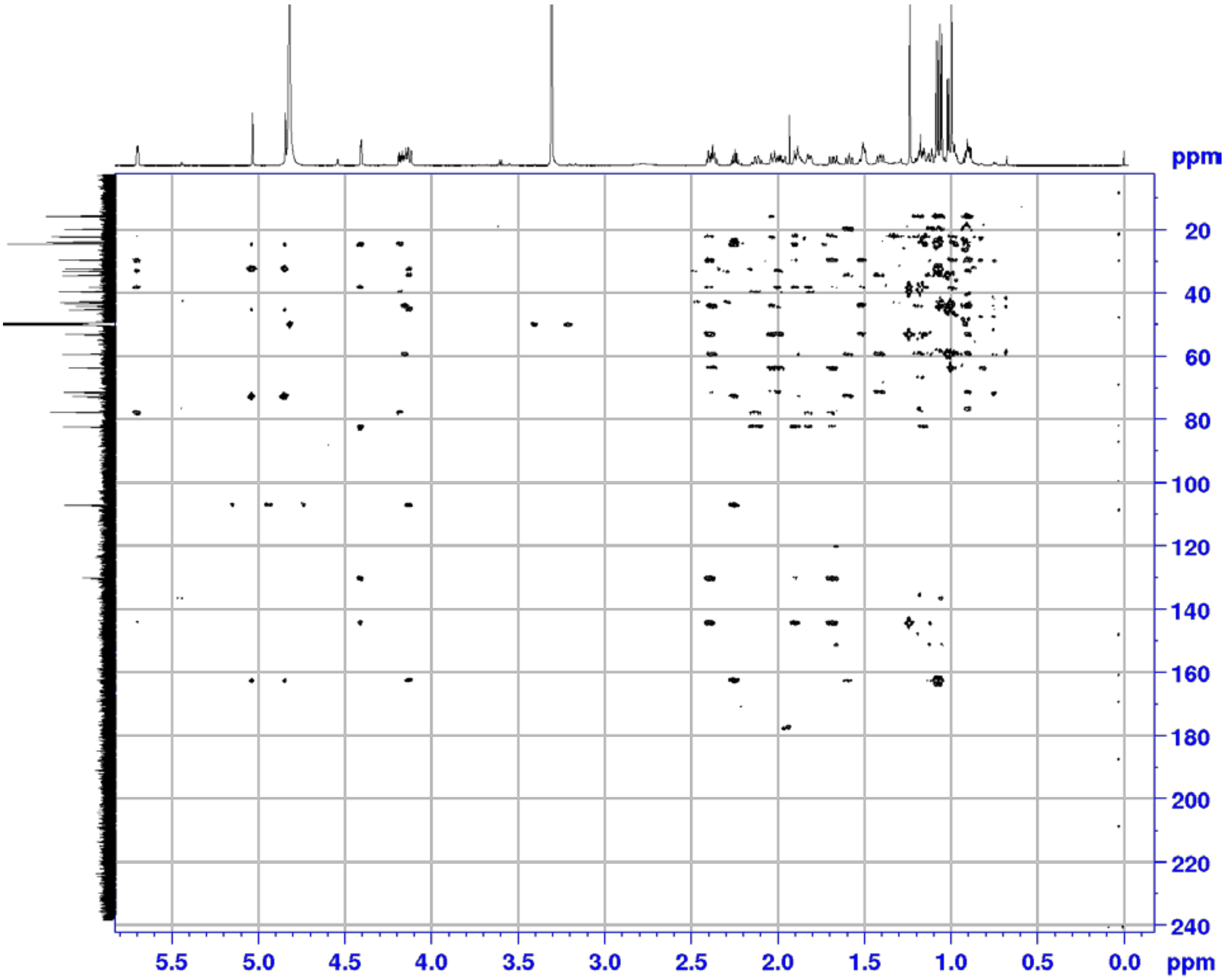

Figure S33. The NOESY (700 MHz, CD_3OD) spectrum of compound **4**

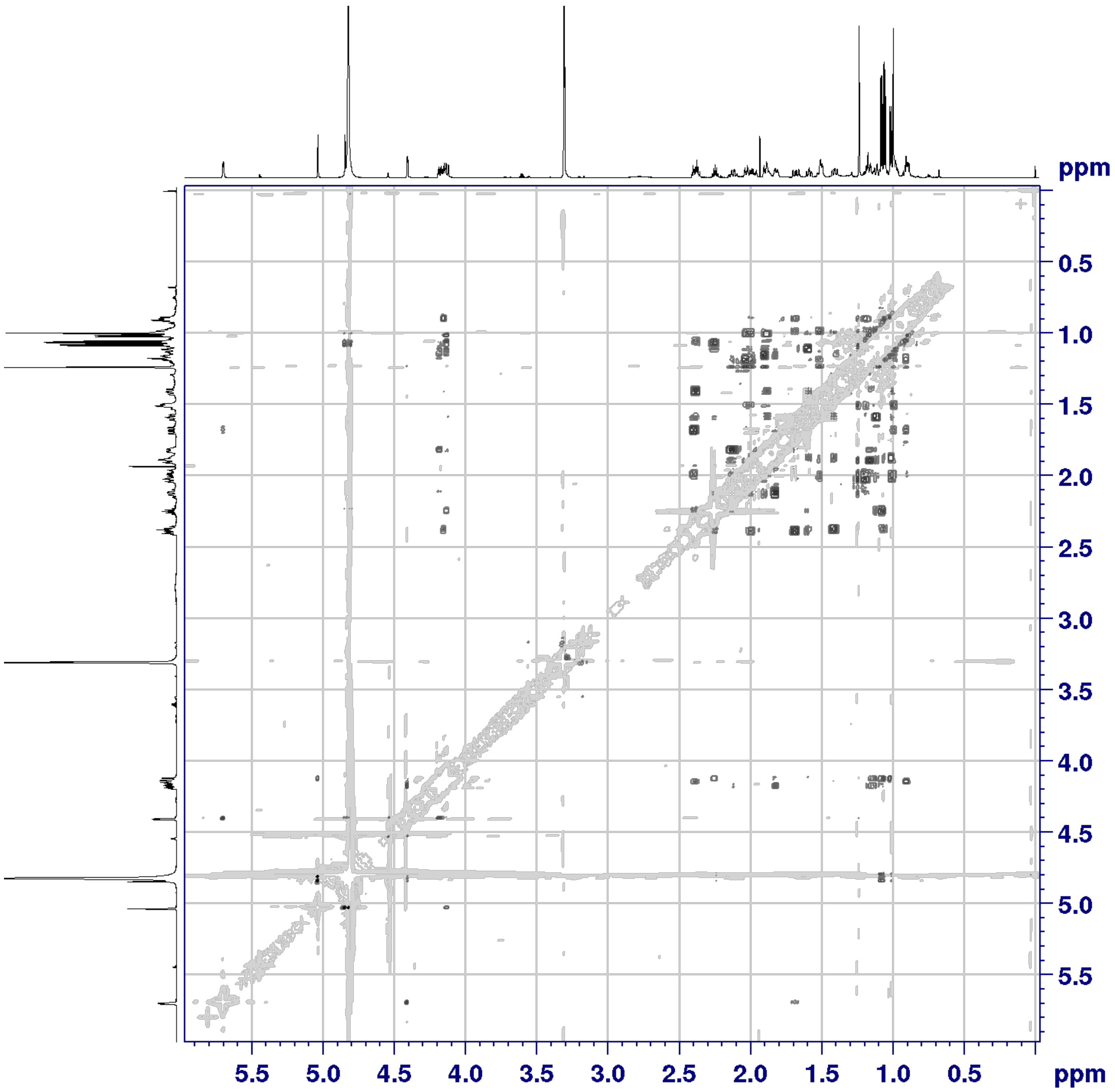

Figure S34. The HRESIMS spectrum of compound **4**

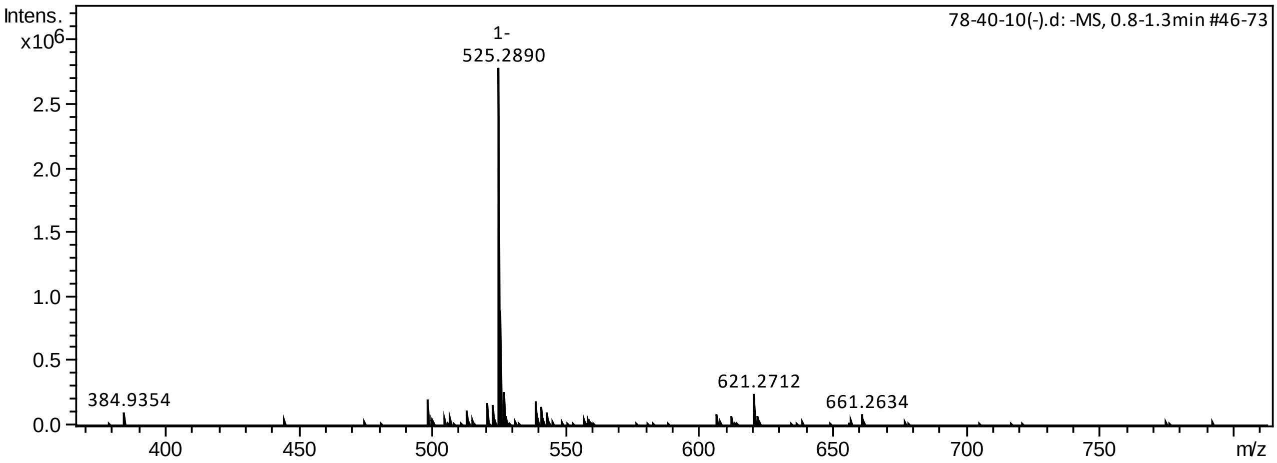

Figure S35. ^{1}H NMR chemical shift differences between **4S** (red) and **4R** (blue)

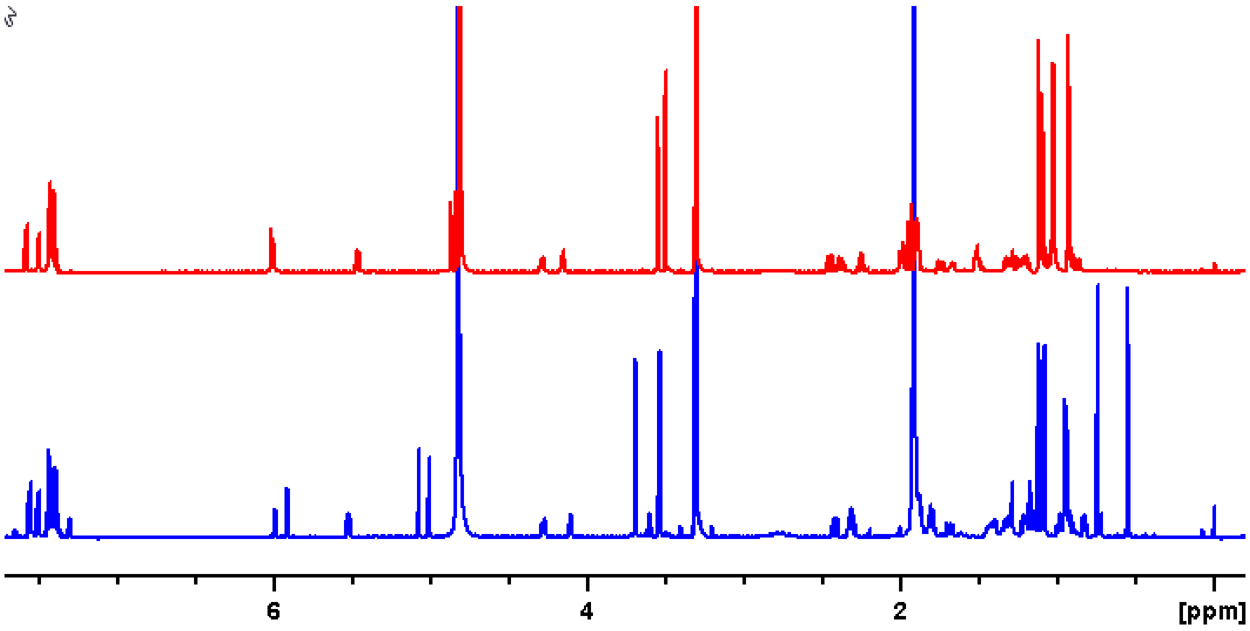

Figure S36. The HRESIMS spectrum of compound **4R** (**4S**)

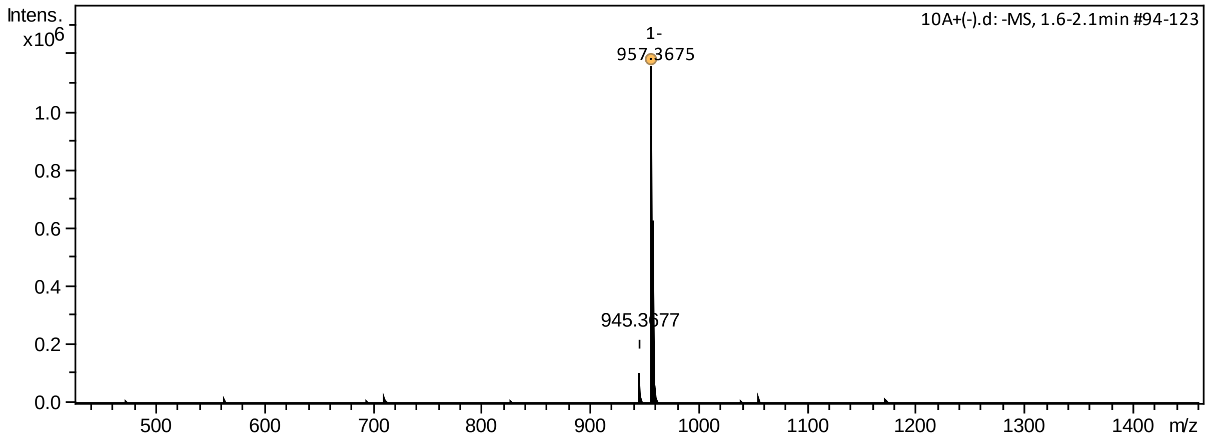

Figure S37. The ^{1}H NMR (500 MHz, CD_3OD) spectrum of compound **5**

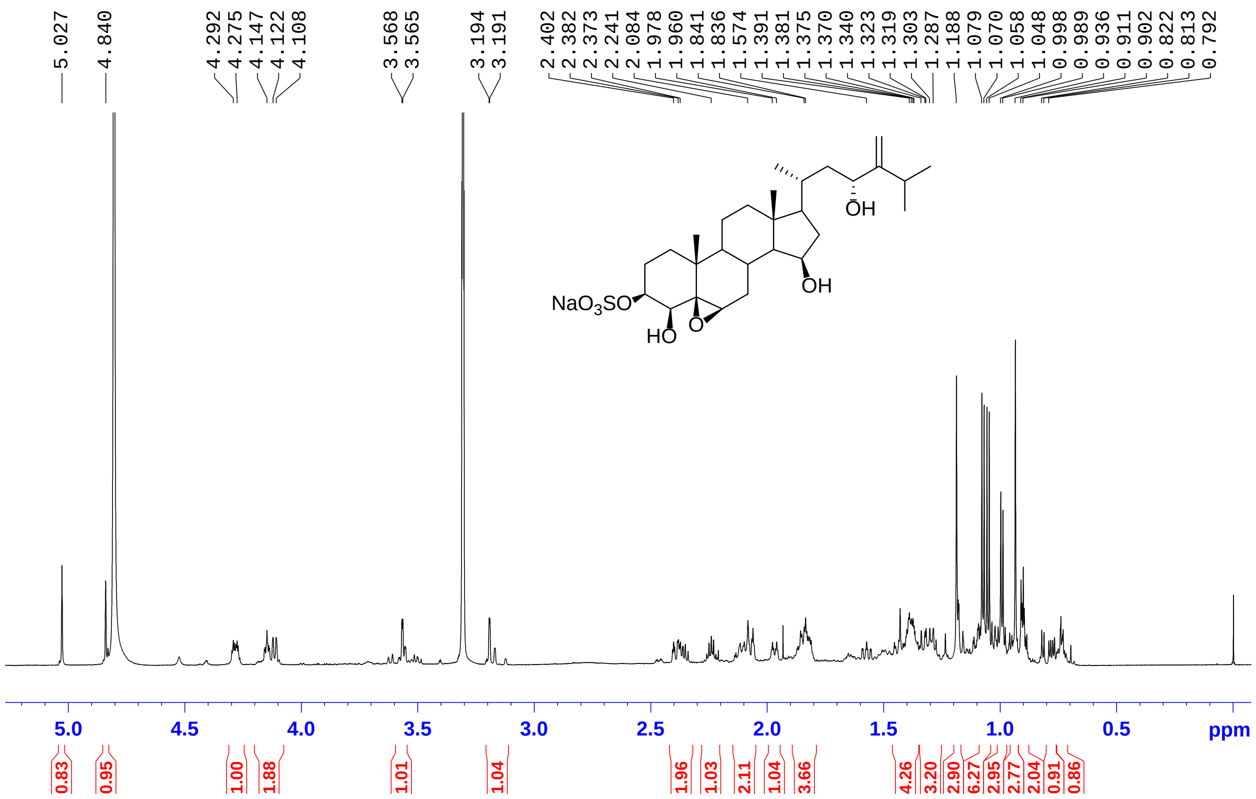

Figure S38. The ^{13}C NMR (125 MHz, CD_3OD) spectrum of compound **5**

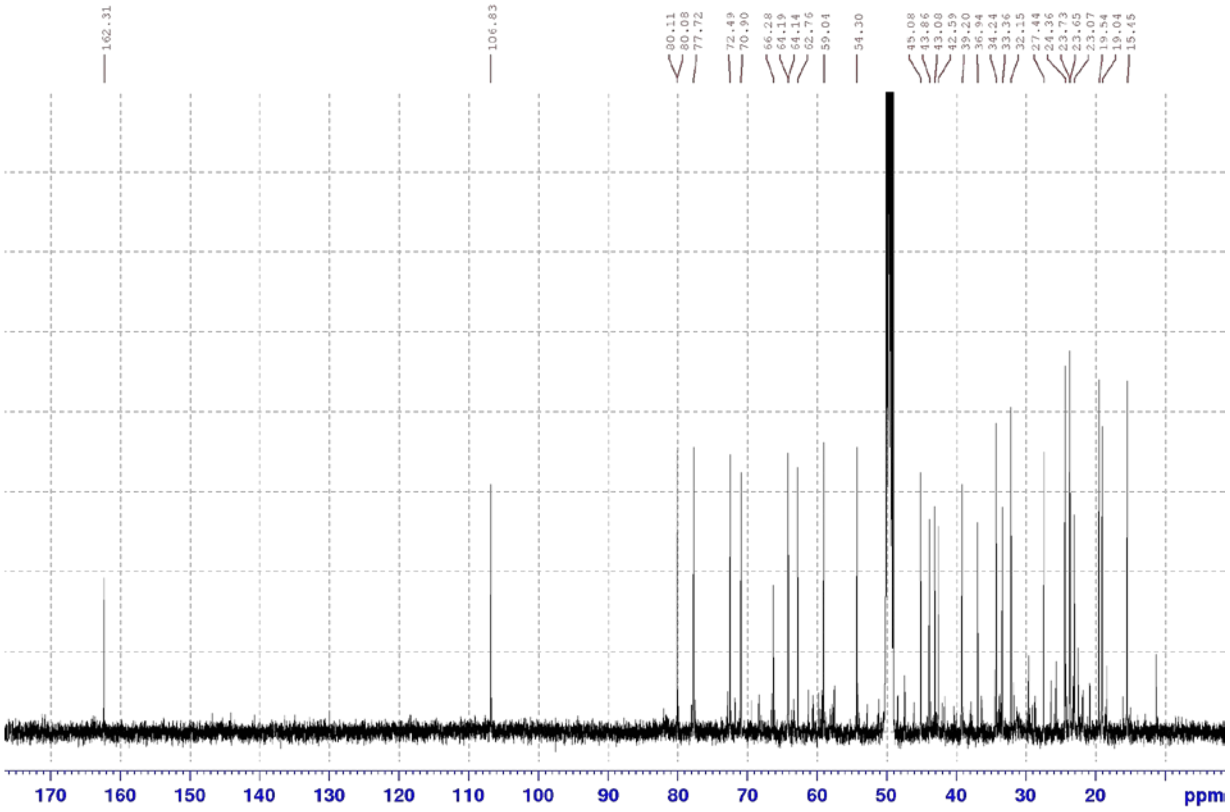

Figure S39. The COSY (500 MHz, CD_3OD) spectrum of compound **5**

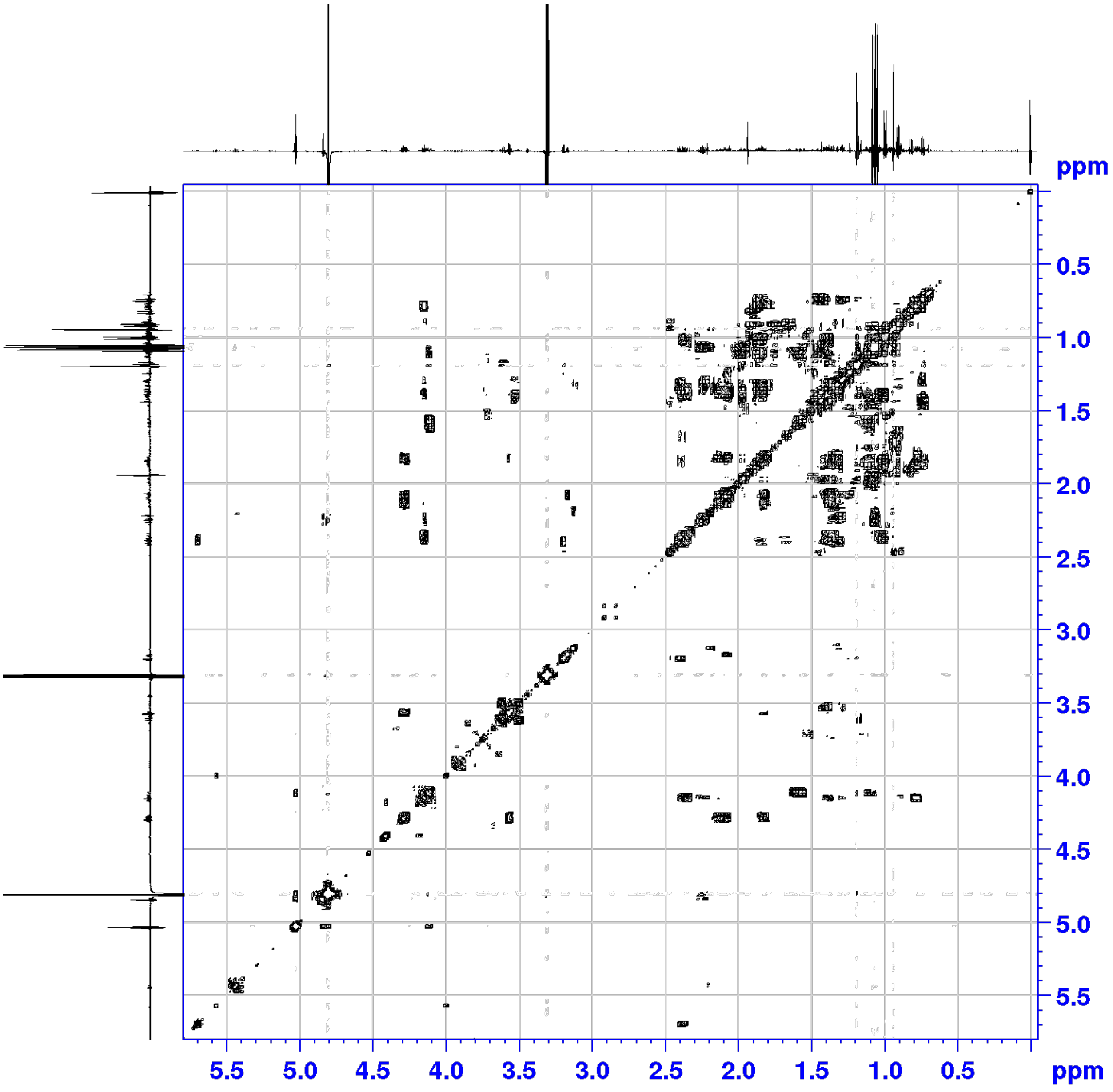

Figure S40. The HSQC (500/125 MHz, CD_3OD) spectrum of compound **5**

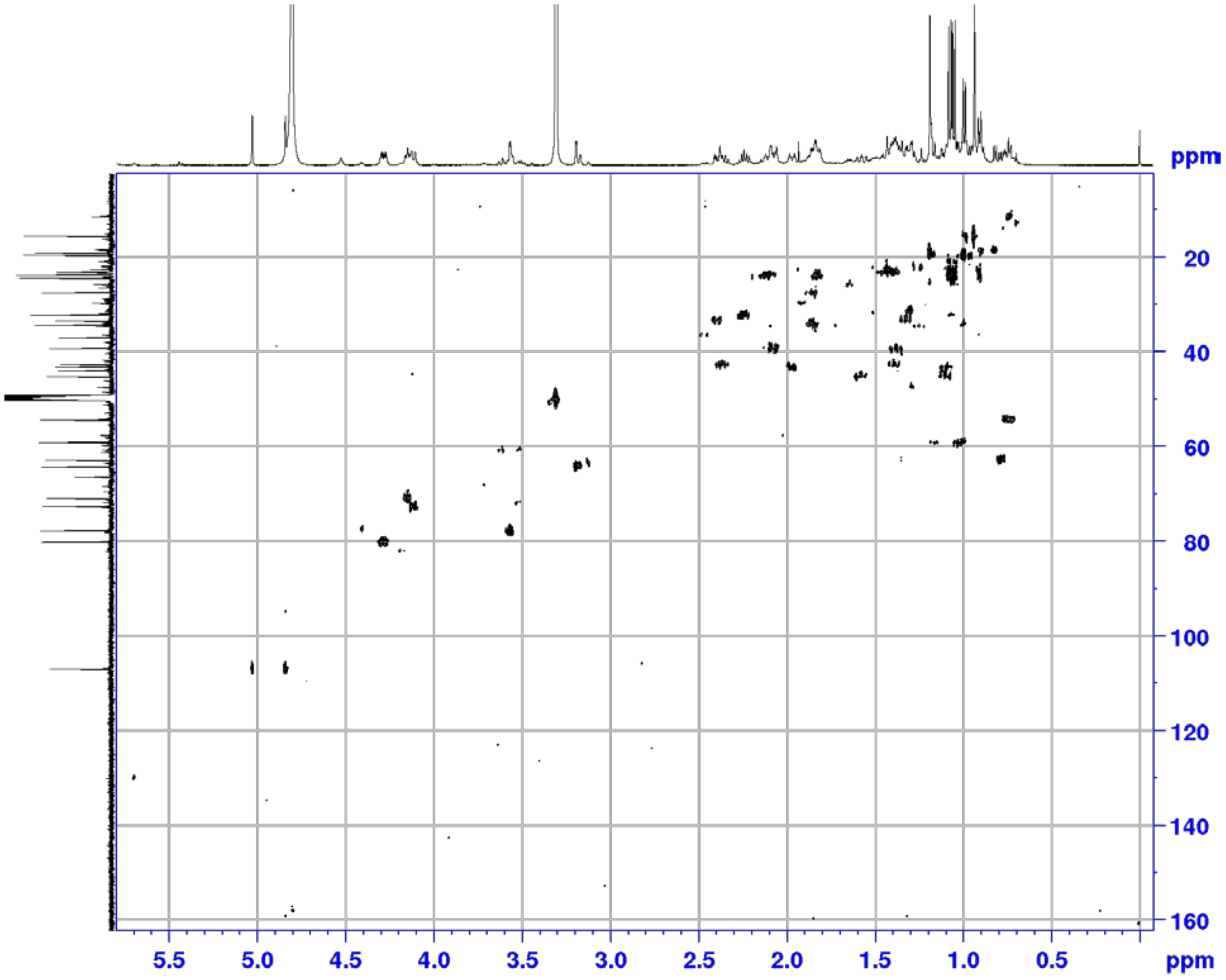

Figure S41. The HMBC (500/125 MHz, CD_3OD) spectrum of compound **5**

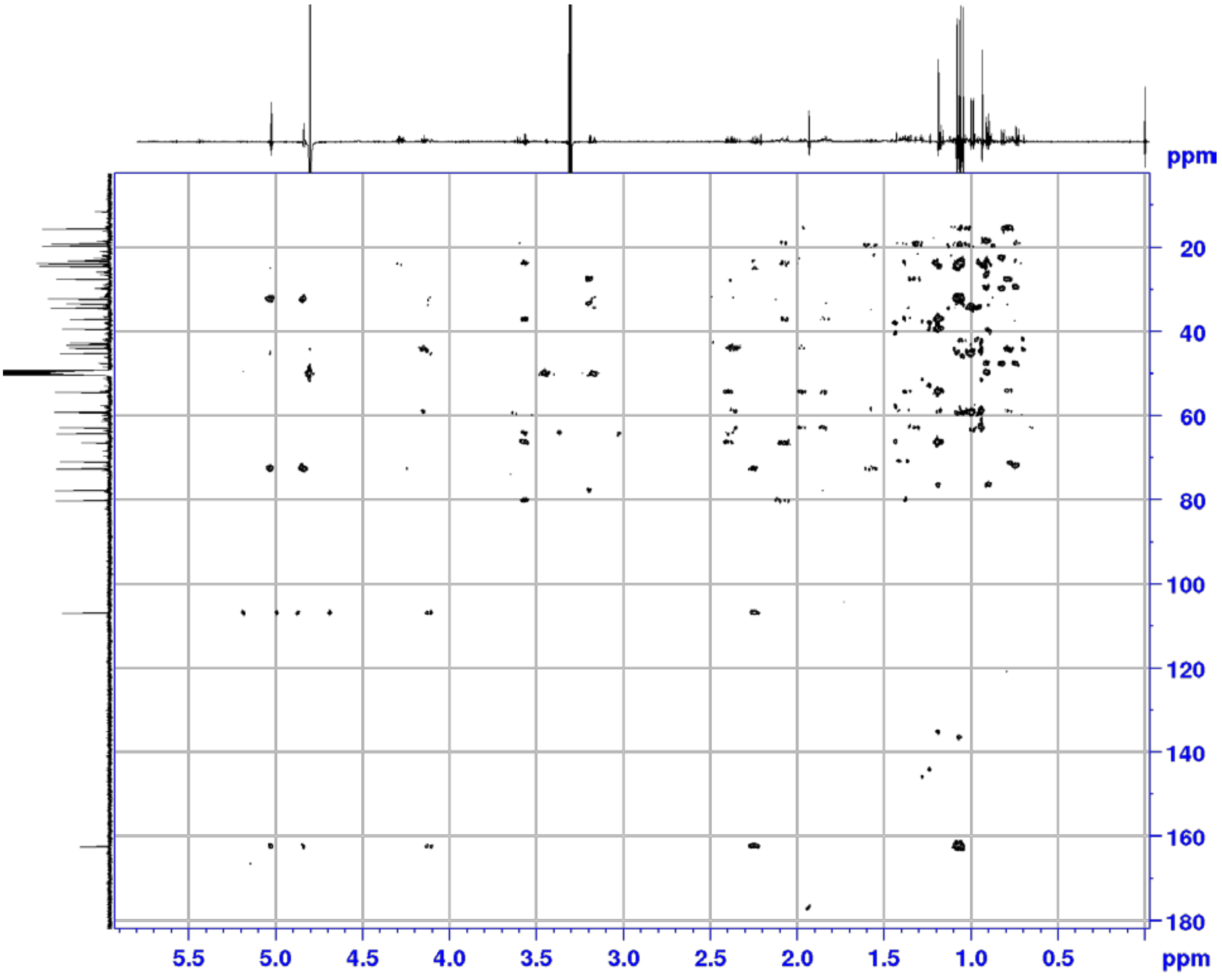

Figure S42. The NOESY (500 MHz, CD_3OD) spectrum of compound **5**

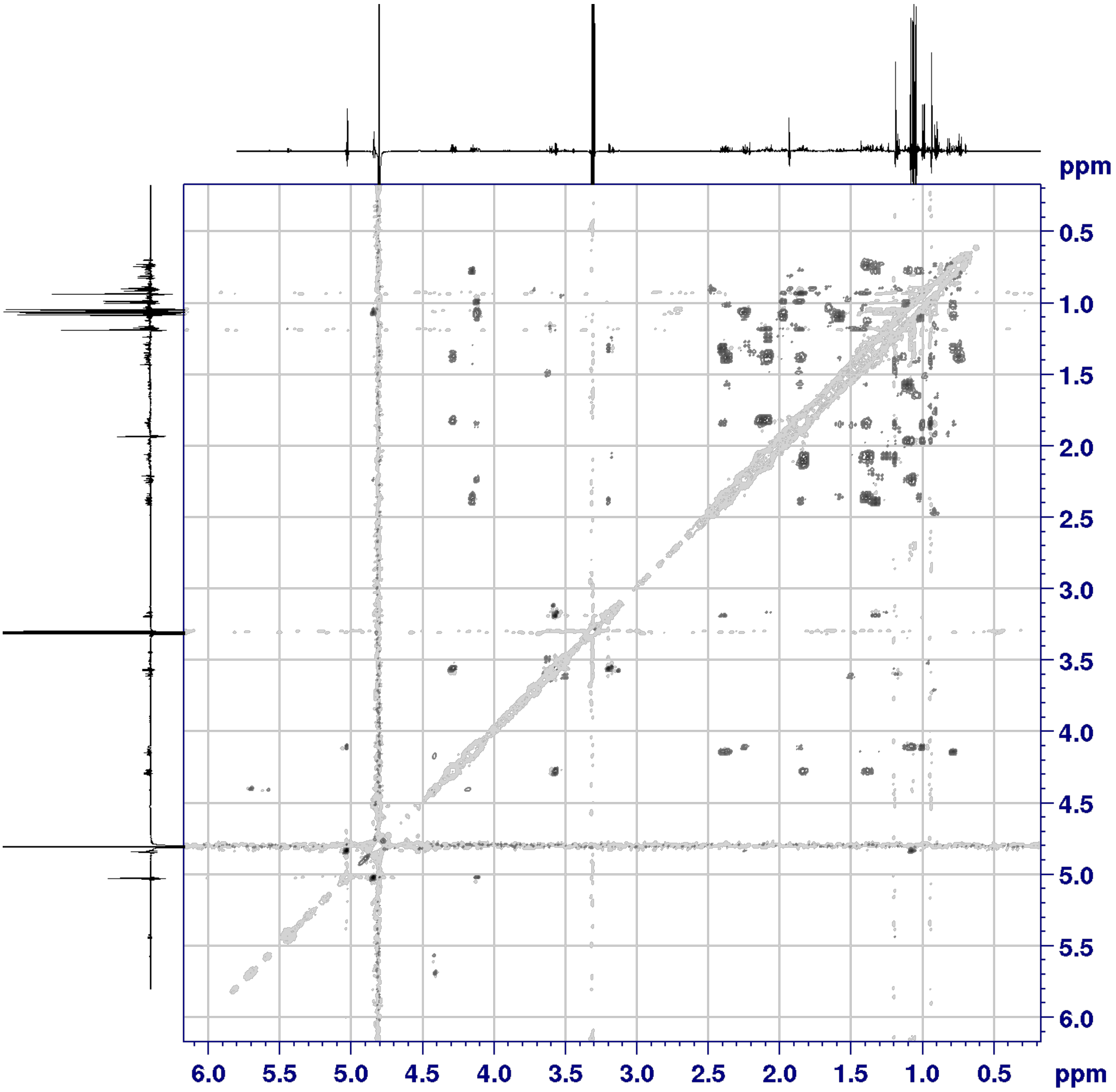

Figure S43. The HRESIMS spectrum of compound **5**

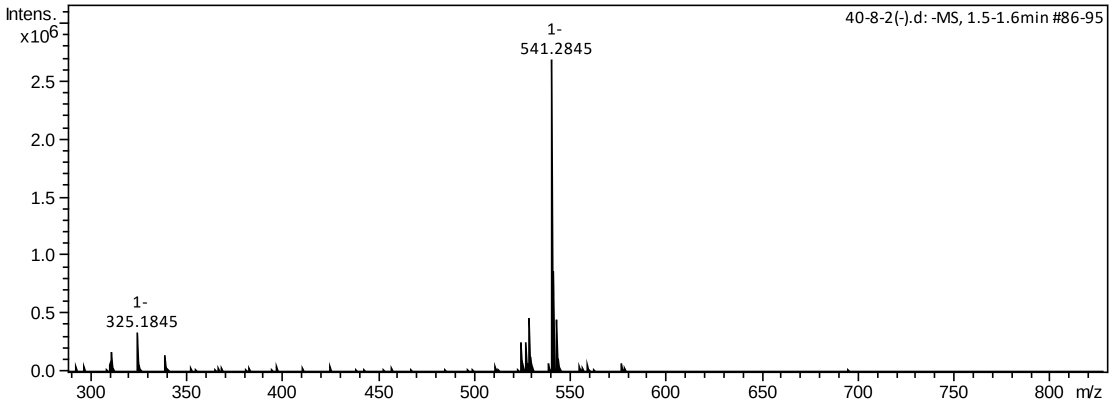

Figure S44. The ^{1}H NMR (700 MHz, CD_3OD) spectrum of compound **6**

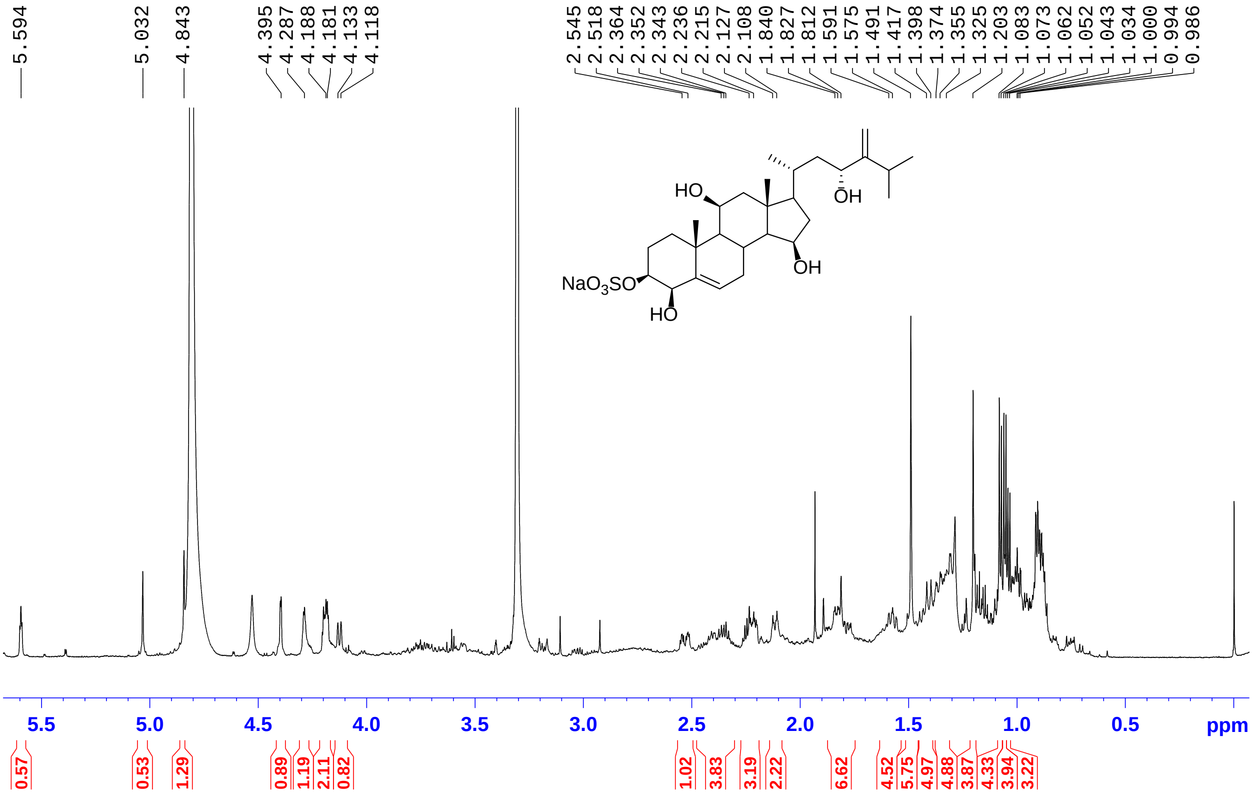

Figure S45. The ^{13}C NMR (175 MHz, CD_3OD) spectrum of compound **6**

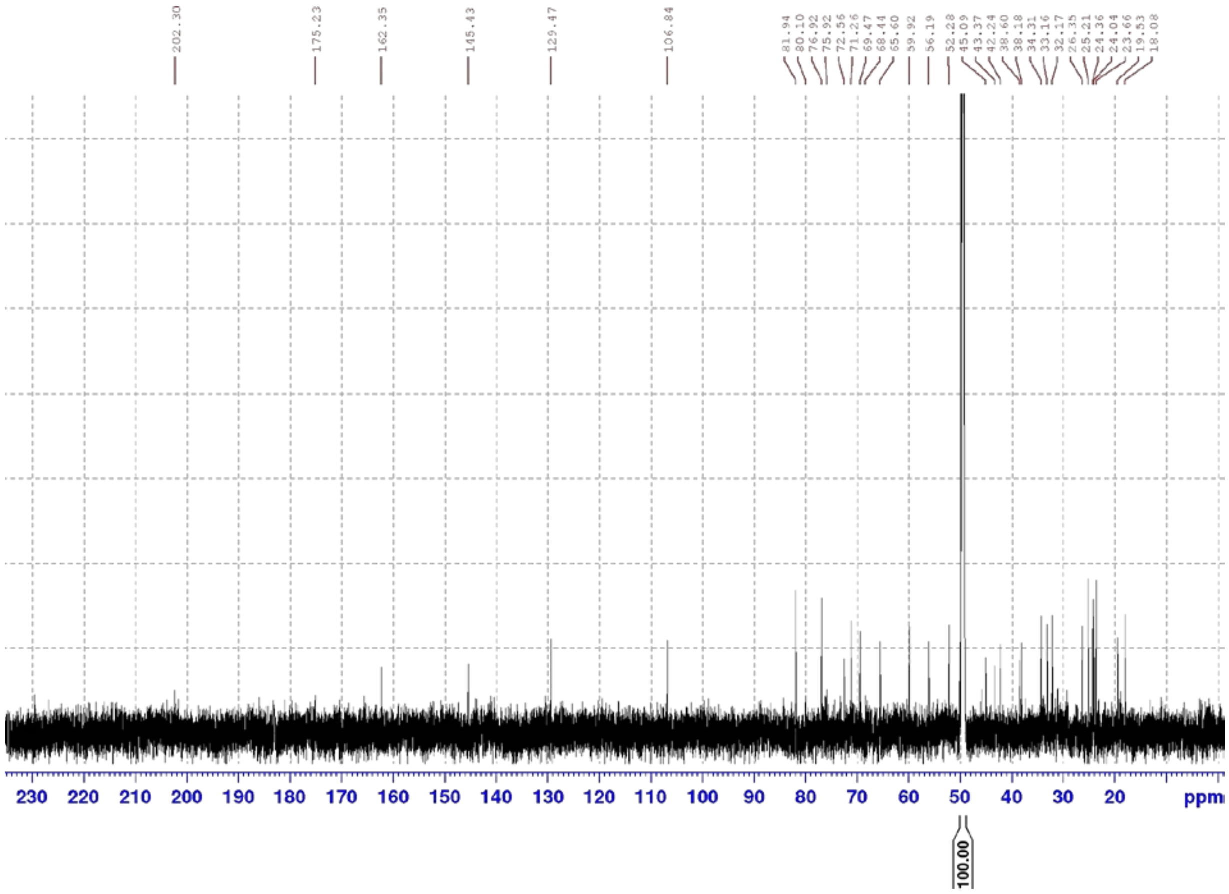

Figure S46. The COSY (700 MHz, CD_3OD) spectrum of compound **6**

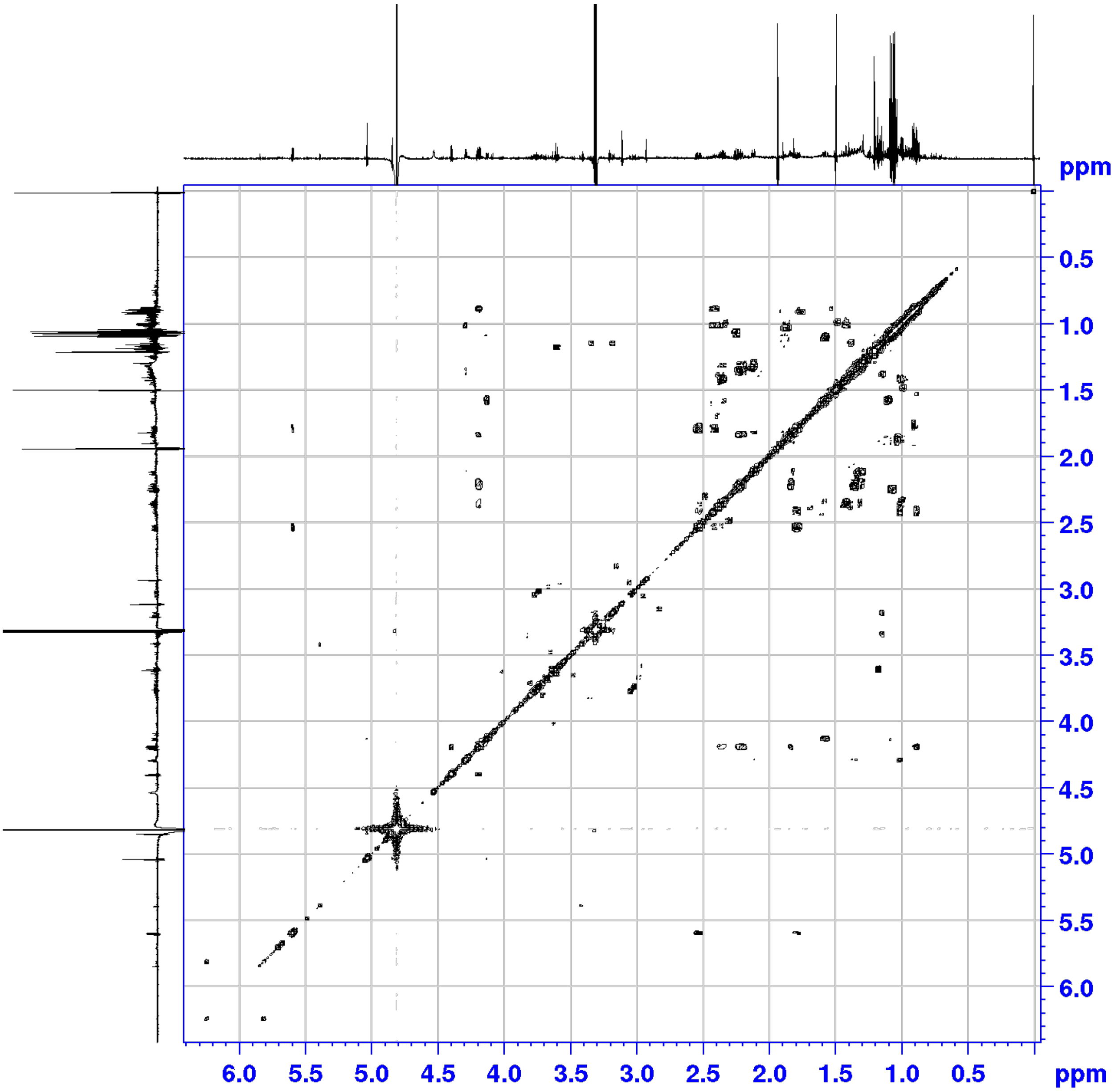

Figure S47. The HSQC (700/175 MHz, CD_3OD) spectrum of compound **6**

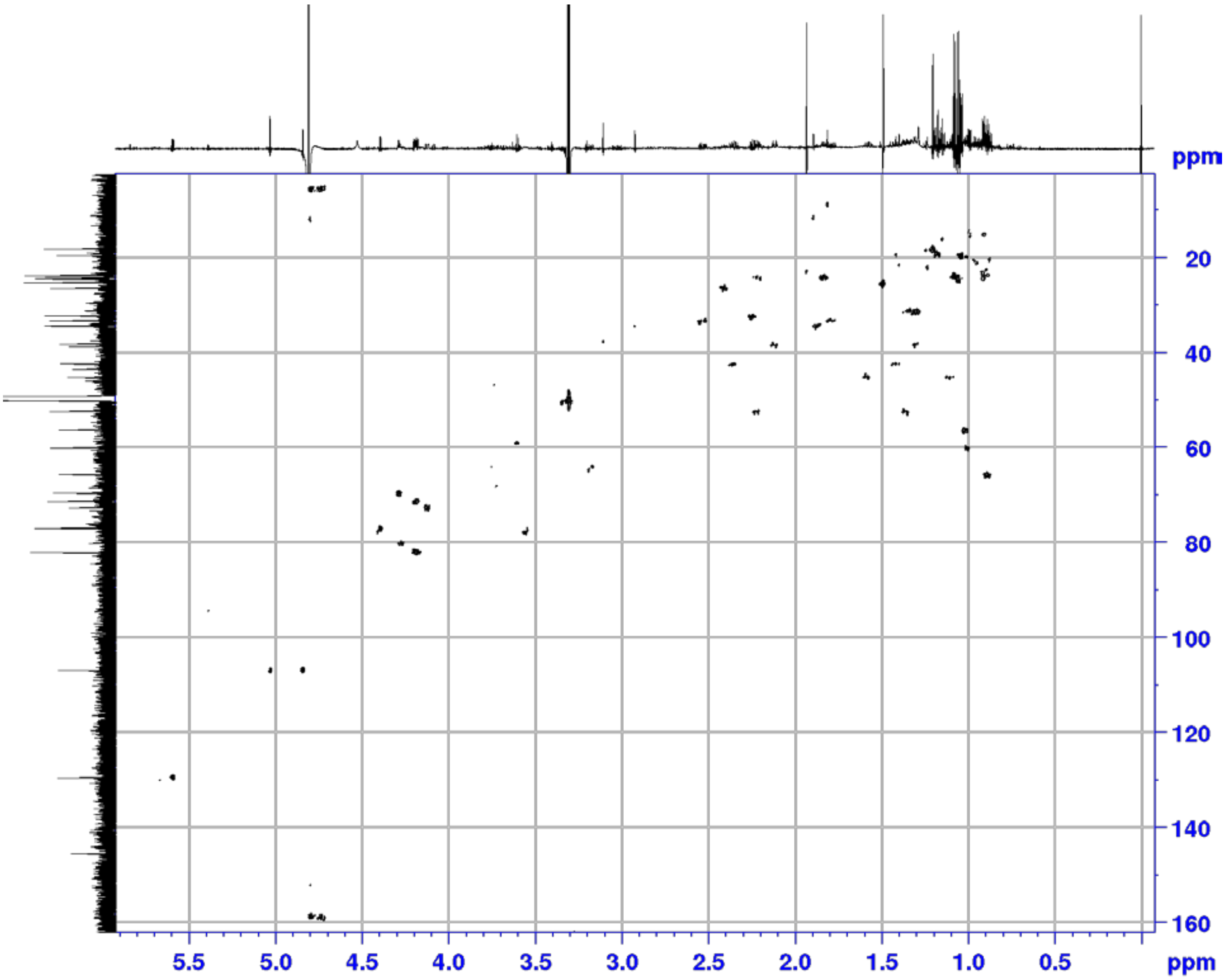

Figure S48. The HMBC (700/175 MHz, CD_3OD) spectrum of compound **6**

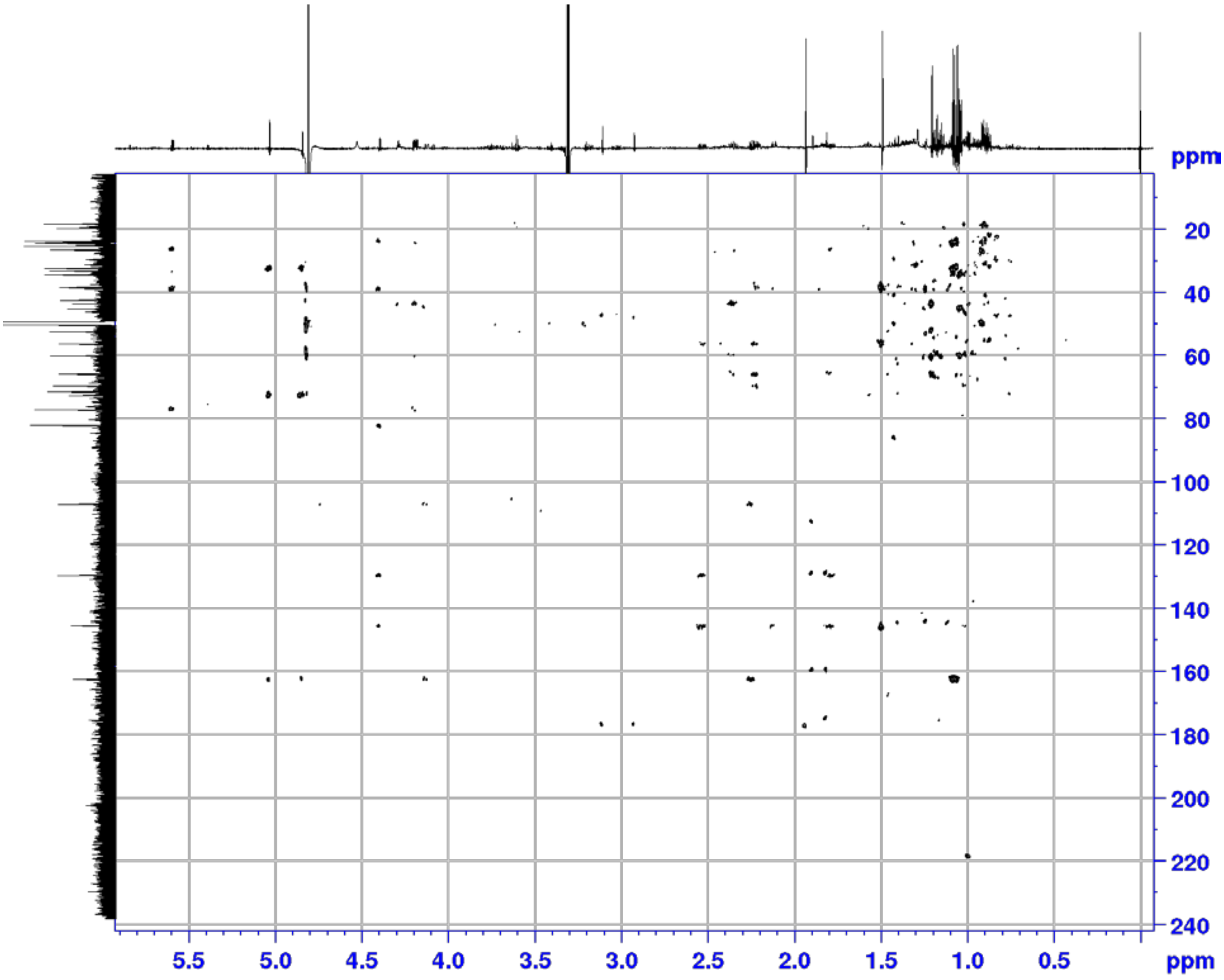

Figure S49. The NOESY (700 MHz, CD_3OD) spectrum of compound **6**

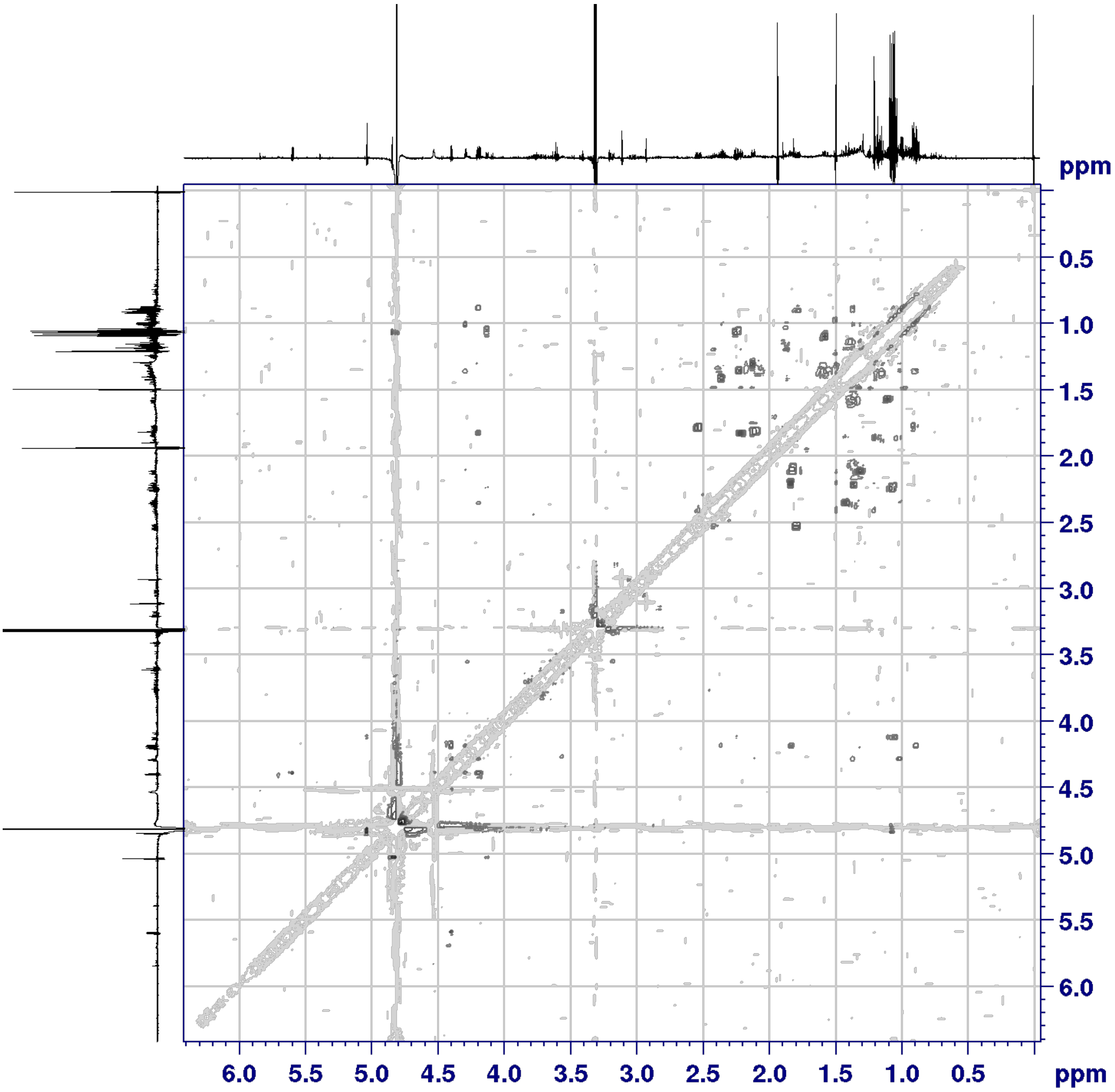

Figure S50. The HRESIMS spectrum of compound **6**

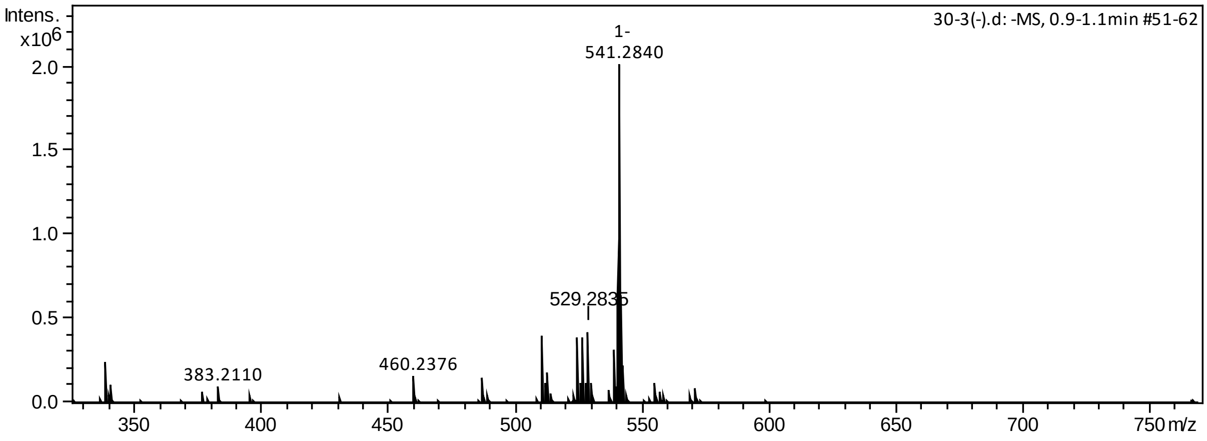

Figure S51. The ^{1}H NMR (700 MHz, CD_3OD) spectrum of compound **7**

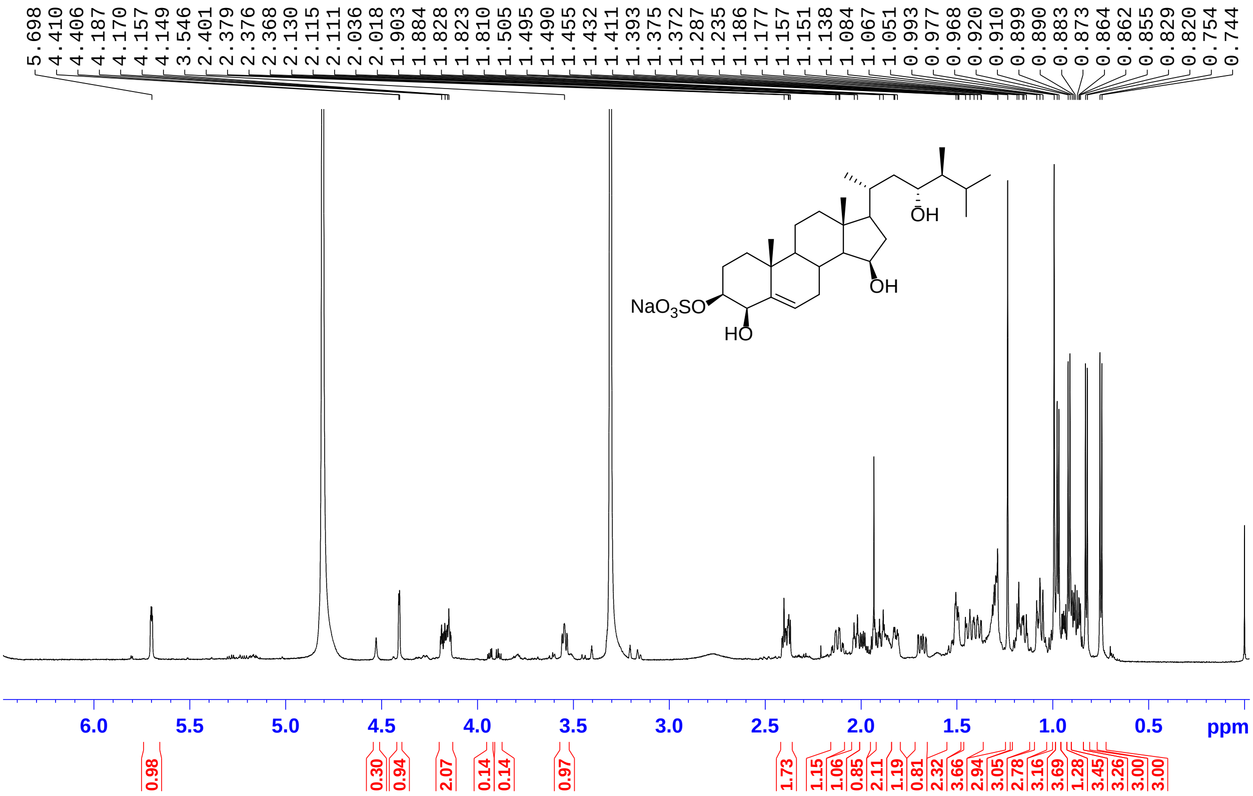

Figure S52. The ^{13}C NMR (175 MHz, CD_3OD) spectrum of compound **7**

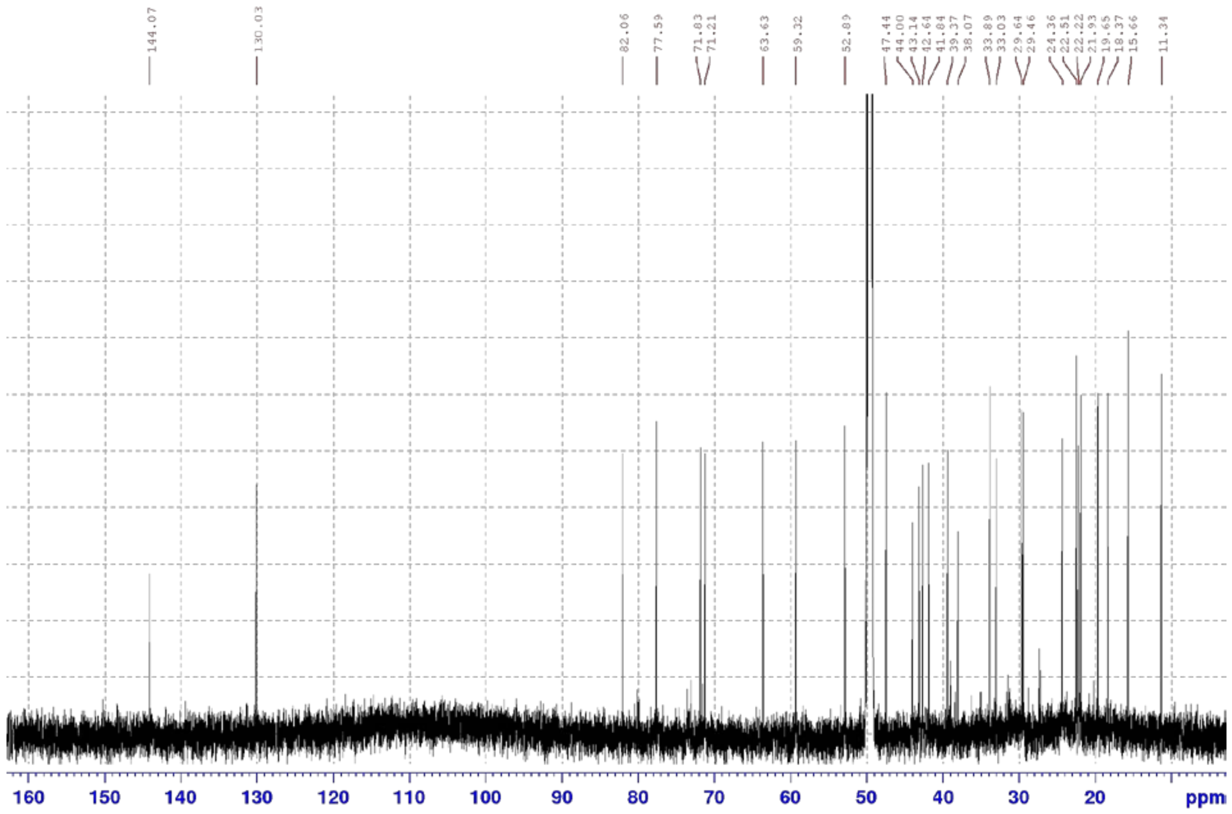

Figure S53. The COSY (700 MHz, CD_3OD) spectrum of compound **7**

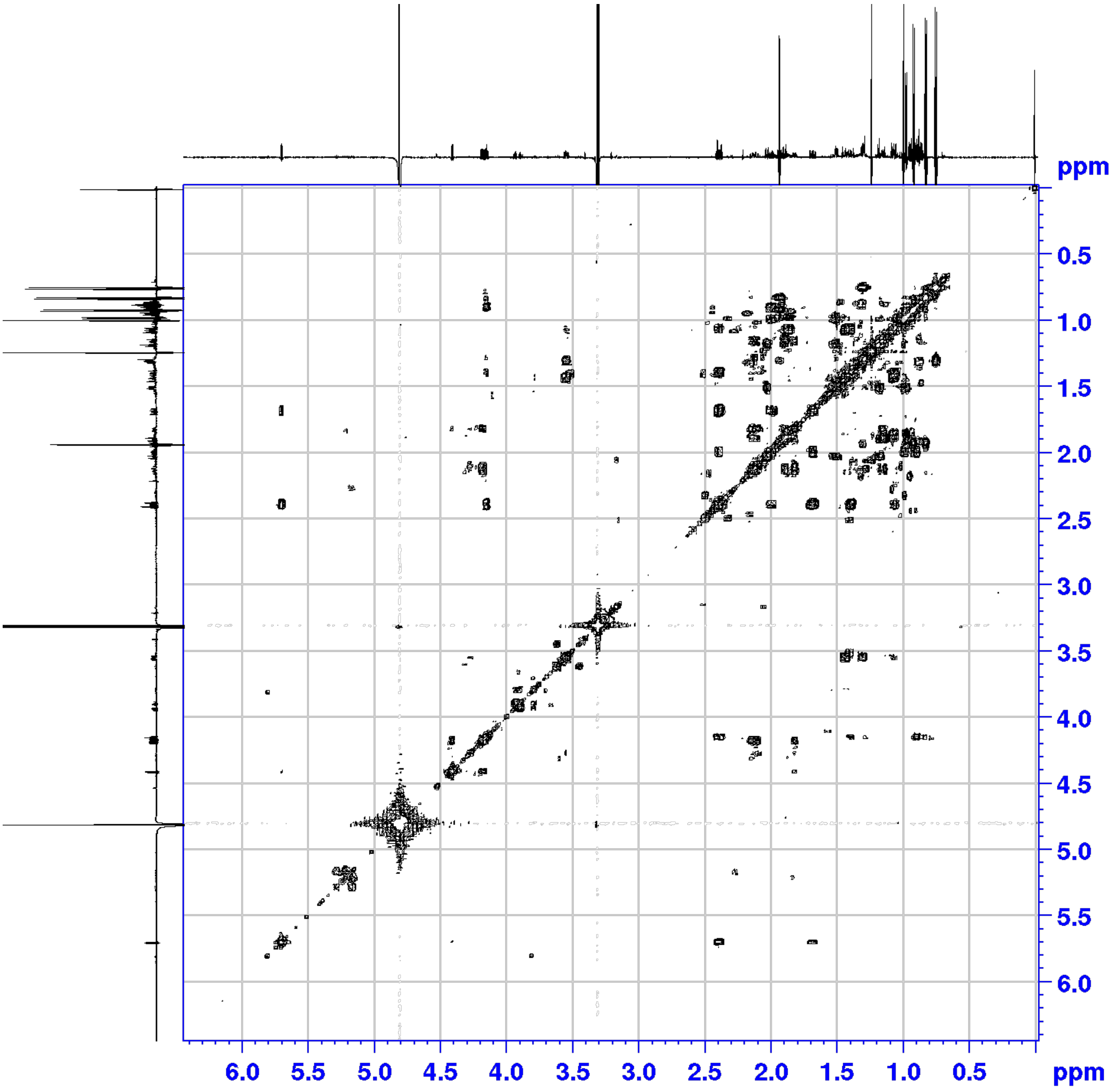

Figure S54. The HSQC (700/175 MHz, CD_3OD) spectrum of compound **7**

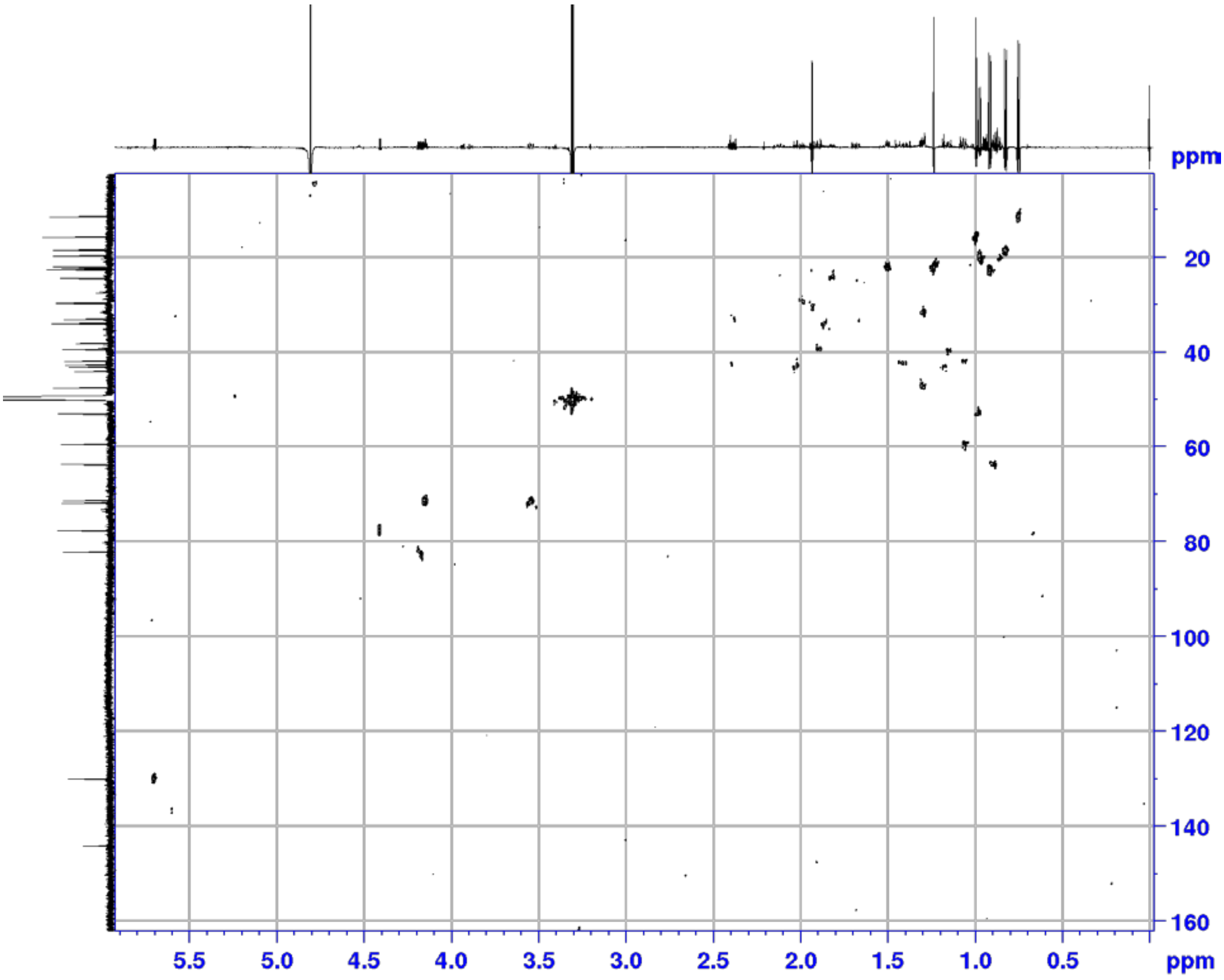

Figure S55. The HMBC (700/175 MHz, CD_3OD) spectrum of compound **7**

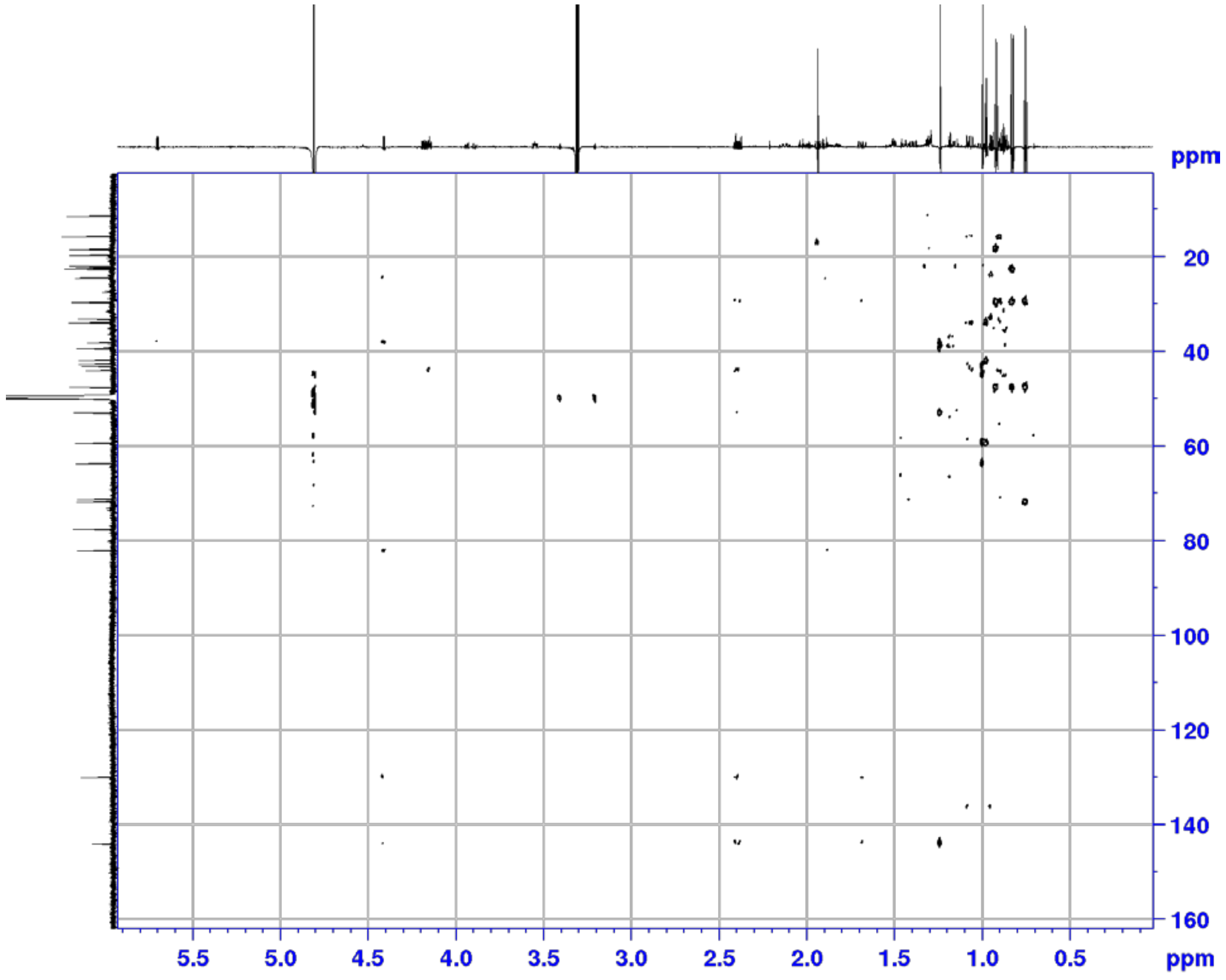

Figure S56. The NOESY (700 MHz, CD_3OD) spectrum of compound **7**

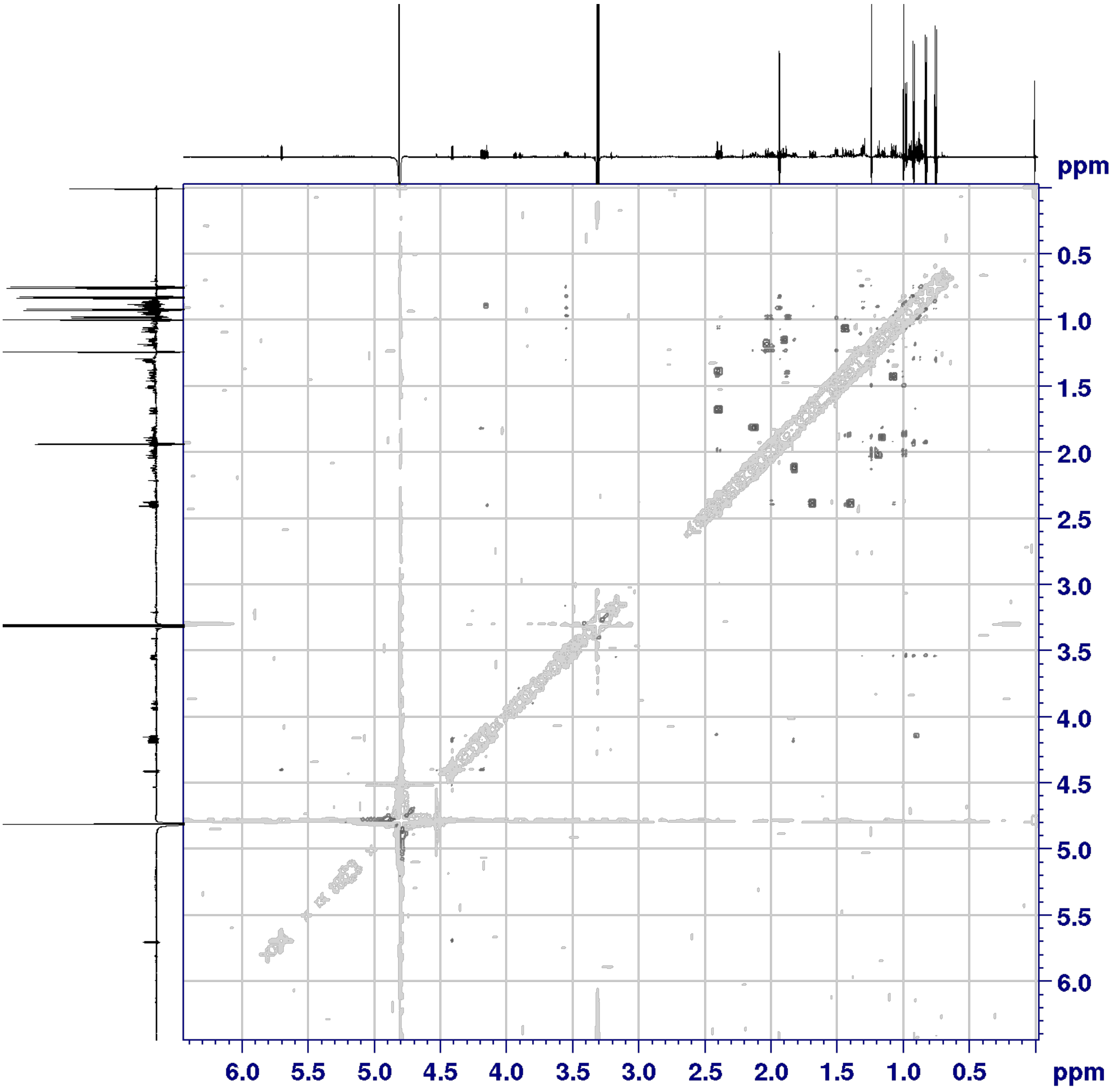

Figure S57. The HRESIMS spectrum of compound **7**

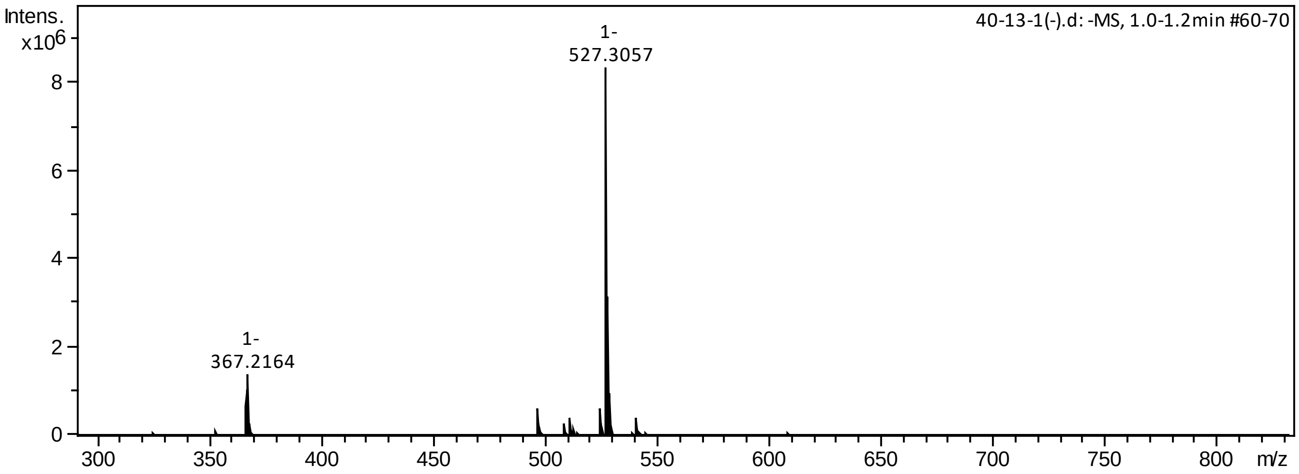

Figure S58. Photo of the sponge *Haliclona gracilis*.

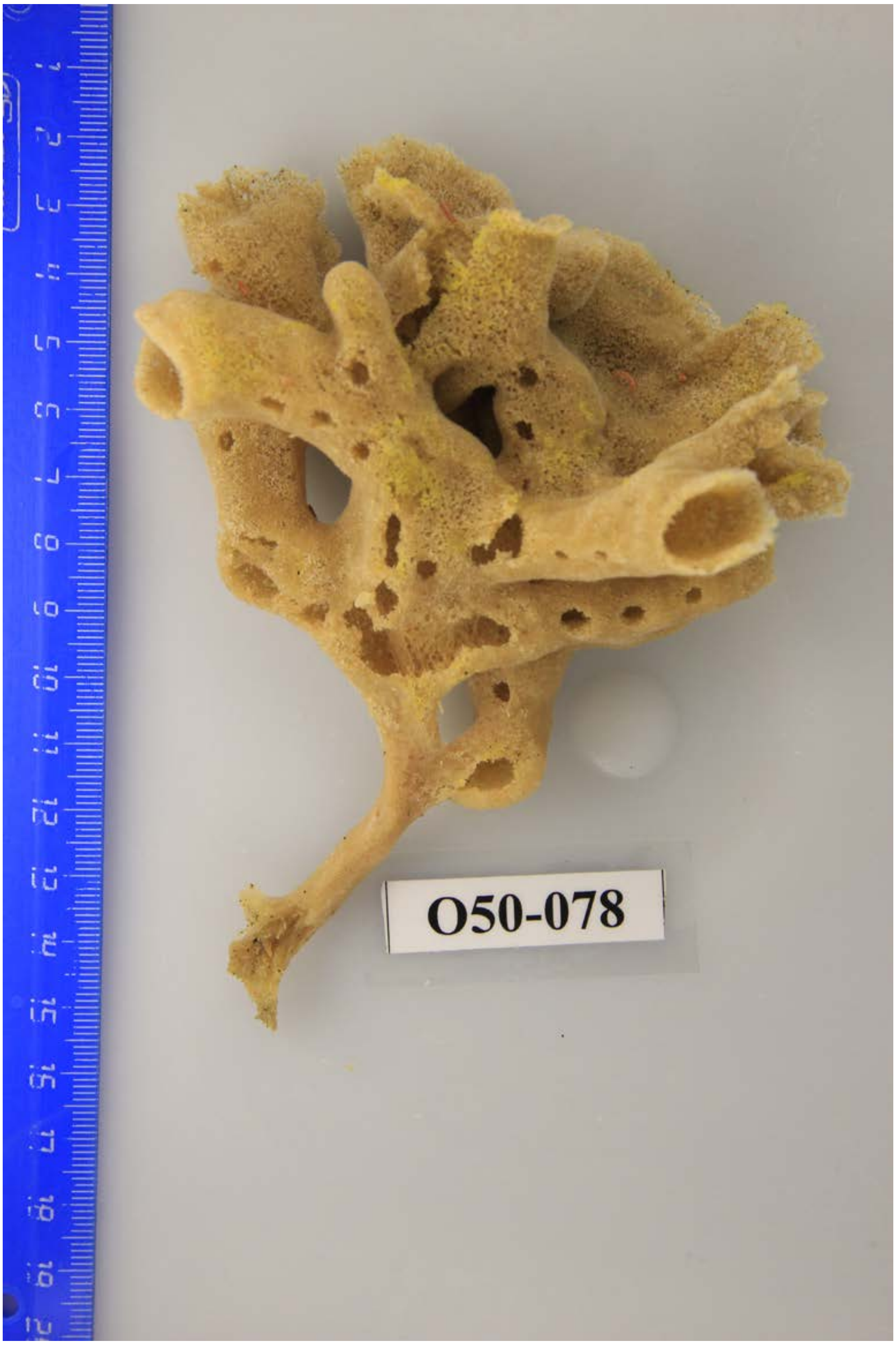

Supplement: Supplementary file 1 [file marinedrugs-18-00454-s001.pdf]
